# Supplementary material for: Adolescent weight management counseling: The effectiveness of an online training program for primary healthcare professionals in Indonesia
Source: PLoS One. 2025 Feb 10;20(2):e0315770. doi: 10.1371/journal.pone.0315770 (PMC11809861; doi:10.1371/journal.pone.0315770)
Supplement: S2 File — (PDF) [file pone.0315770.s002.pdf]

Knowledge Assessment

Group	Pre test	Post test
kontrol	72.34	68.09
kontrol	70.21	65.96
kontrol	31.91	51.06
kontrol	72.34	72.34
kontrol	87.23	65.96
kontrol	63.83	61.7
kontrol	59.57	59.57
kontrol	51.06	65.96
kontrol	51.06	72.34
kontrol	63.83	80.85
kontrol	68.09	76.6
kontrol	68.09	55.32
kontrol	59.57	78.72
kontrol	61.7	57.45
kontrol	74.47	61.7
kontrol	76.6	61.7
kontrol	57.45	76.6
kontrol	46.81	65.96
kontrol	51.06	57.45
kontrol	70.21	61.7
kontrol	65.96	61.7
kontrol	63.83	61.7
kontrol	42.55	74.47
kontrol	61.7	55.32
kontrol	70.21	76.6
kontrol	72.34	89.36
kontrol	57.45	80.85
kontrol	68.09	51.06
kontrol	72.34	80.85
kontrol	76.6	
kontrol	65.96	
intervensi	48.94	82.98
intervensi	68.09	91.49
intervensi	76.6	78.72
intervensi	57.45	91.49
intervensi	63.83	91.49

intervensi	57.45	89.36
intervensi	74.47	68.09
intervensi	65.96	89.36
intervensi	57.45	80.85
intervensi	65.96	55.32
intervensi	74.47	85.11
intervensi	46.81	72.34
intervensi	76.6	68.09
intervensi	70.21	85.11
intervensi	82.98	51.06
intervensi	57.45	85.11
intervensi	46.81	70.21
intervensi	72.34	85.11
intervensi	48.94	89.36
intervensi	59.57	85.11
intervensi	65.96	76.6
intervensi	70.21	48.94
intervensi	55.32	85.11
intervensi	78.72	76.6
intervensi	65.96	91.49
intervensi	80.85	82.98
intervensi	68.09	
intervensi	65.96	
intervensi	48.94	
intervensi	51.06	
intervensi	55.32	
intervensi	70.21	

Pre test A dan B

Group Statistics					
	Nama Kelompok	N	Mean	Std. Deviation	Std. Error Mean
Nilai	kontol	31	63.6923	11.31229	2.03175
	intervensi	32	64.0306	10.49774	1.85576

Independent Samples Test

		Levene's Test for Equality of Variances		t-test for Equality of Means						
									95% Confidence Interval of the Difference	
		F	Sig.	t	df	Sig. (2-tailed)	Mean Difference	Std. Error Difference	Lower	Upper
Nilai	Equal variances assumed	.029	.865	-.123	61	.902	-.33837	2.74839	-5.83411	5.15737
	Equal variances not assumed			-.123	60.313	.903	-.33837	2.75170	-5.84199	5.16526

Post test A dan post test B

Group Statistics

	Kel_post	N	Mean	Std. Deviation	Std. Error Mean
Postest	kontrol	29	67.2048	9.98851	1.85482
	intervensi	26	79.1338	12.36266	2.42452

Independent Samples Test

		Levene's Test for Equality of Variances		t-test for Equality of Means						
									95% Confidence Interval of the Difference	
		F	Sig.	t	df	Sig. (2-tailed)	Mean Difference	Std. Error Difference	Lower	Upper
Postest	Equal variances assumed	.552	.461	-3.954	53	.000	-11.92902	3.01720	-17.98076	-5.87727
	Equal variances not assumed			-3.908	48.112	.000	-11.92902	3.05264	-18.06640	-5.79164

Pre dan post A dan B

Paired Samples Statistics

		Mean	N	Std. Deviation	Std. Error Mean
Pair 1	PreA	63.1690	29	11.43201	2.12287
	postA	67.2048	29	9.98851	1.85482
Pair 2	preB	64.9769	26	10.70098	2.09863
	postB	79.1338	26	12.36266	2.42452

Paired Samples Correlations				
		N	Correlation	Sig.
Pair 1	PreA & postA	29	.145	.454
Pair 2	preB & postB	26	-.297	.141

Paired Samples Test									
		Paired Differences					t	df	Sig. (2-tailed)
		Mean	Std. Deviation	Std. Error Mean	95% Confidence Interval of the Difference				
					Lower	Upper			
Pair 1	PreA - postA	-4.03586	14.04942	2.60891	-9.37998	1.30825	-1.547	28	.133
Pair 2	preB - postB	-14.15692	18.59563	3.64690	-21.66786	-6.64599	-3.882	25	.001

Below is the result of **Counseling skill assessment** which was divided into 2 types of assessment

1. Quantitative using ABC-CAT tool and
2. Qualitative using narrative commentary from the trained assesor

No	Kode	Introduction	Bridging	Asking adolescent to speak on their own	Psychosocial Screening	Open ended questions	Affirmation
1	B1R1	1	2	2	2	9	9
2	B3R1	3	1	2	2	9	0
3	B5R1	2	1	0	2	9	0
4	B7R1	0	1	2	2	9	6
5	B9R1						
6	B11R1	2	2	0	2	9	6
7	B13R1	1	1	2	1	9	0
8	B15R1	2	1	0	1	3	0
9	B17R1	3	1	2	3	9	6
10	B19R1	1	1	2	1	6	0
11	B21R1	1	2	2	2	6	0
12	B23R1	1	1	2	2	6	0
13	B25R1	2	1	0	3	9	0
14	B27R1	0	1	2	1	3	6
15	B29R1	2	1	0	3	9	9
16	B31R1	2	1	0	2	6	0
17	A1R1	2	1	0	2	6	0
18	A3R1	3	1	0	1	3	0
19	A5R1	3	2	2	2	6	3
20	A7R1						
21	A9R1	2	1	0	2	9	9
22	A11R1	0	1	0	1	9	9
23	A13R1	2	2	0	3	6	0
24	A15R1	0	1	0	1	6	9
25	A17R1	0	1	0	2	6	9
26	A19R1	2	2	0	1	9	0
27	A21R1	3	2	0	1	9	0
28	A23R1	1	1	2	1	6	0
29	A25R1	3	1	0	3	9	3
30	A27R1	2	1	0	2	6	0
31	A29R1	1	1	2	3	9	6
32	A31R1	2	3	0	3	9	0
33	A33R1	0	0	0	1	0	0

Reflective Listening	Summary	Engaging	Focusing	Evoking	Planning	Providing Information
9	3	9	9	9	9	4
6	3	6	6	0	0	2
6	0	6	6	0	0	2
6	0	6	6	0	0	2
6	0	6	6	9	6	2
6	0	6	3	0	3	4
6	3	0	0	0	3	2
6	0	9	6	9	0	0
0	3	0	3	0	0	2
6	6	0	0	0	0	4
0	6	3	0	0	0	2
9	3	6	6	0	0	4
6	3	3	0	0	0	2
6	6	6	3	9	0	2
6	3	6	3	0	0	2
0	3	3	0	0	0	2
0	3	0	3	0	3	2
9	0	0	6	0	0	2
6	6	6	3	3	0	2
9	0	6	0	9	0	4
6	0	0	6	0	0	2
6	0	6	6	0	0	2
9	0	6	6	0	0	2
6	6	3	0	0	0	2
6	0	3	6	9	3	2
6	0	3	3	0	0	2
6	0	6	3	6	0	2
6	6	0	3	0	3	2
6	6	9	9	9	0	2
6	6	3	0	3	6	2
0	0	0	0	0	0	0

Parental Involvement	total
0	77
6	46
2	37
0	40
6	62
0	36
6	27
3	57
0	19
0	29
0	23
6	49
6	33
6	62
3	34
3	22
3	22
6	41
3	52
3	51
6	33
6	43
6	47
6	37
3	47
0	25
3	45
6	37
0	63
3	46
0	1

No	Kode	Introduction	Bridging	Asking adolescent to speak on their own	Psychosocial Screening	Open ended questions
1	B1R2	1	3	0	1	9
2	B3R2	1	2	0	2	9
3	B5R2	3	3	0	2	9
4	B7R2	0	2	2	2	9
5	B9R2	1	3	1	2	9
6	B11R2	3	3	3	3	9
7	B13R2	1	2	2	2	9
8	B15R2	3	3	0	1	9
9	B17R2	1	2	0	3	9
10	B19R2	0	2	0	0	9
11	B21R2	3	3	2	3	9
12	B23R2	1	1	0	1	6
13	B25R2	3	2	0	2	9
14	B27R2	1	2	1	2	3
15	B29R2	1	2	2	2	9
16	B31R2	2	2	1	2	6
17	A1R2	3	0	0	0	0
18	A3R2	1	2	0	2	9
19	A5R2	3	3	1	3	9
20	A7R2	2	3	1	0	9
21	A9R2	2	3	0	3	9
22	A11R2	1	1	0	0	0
23	A13R2	1	3	1	3	6
24	A15R2	0	2	0	3	9
25	A17R2	dialihkan				
26	A19R2	2	2	0	2	6
27	A21R2	3	2	0	2	9
28	A23R2	1	2	0	1	3
29	A25R2	1	3	0	2	9
30	A27R2	1	1	0	2	9
31	A29R2	3	2	0	3	9
32	A31R2	3	3	0	3	9
33	A33R2	0	0	0	0	0

Affirmation	Reflective Listening	Summary	Engaging	Focusing	Evoking	Planning
9	9	9	6	6	6	6
9	9	9	9	9	6	6
6	9	6	9	9	9	9
6	9	9	9	9	6	3
9	9	6	6	9	9	9
9	9	9	9	9	6	9
6	9	9	9	9	9	6
9	9	9	6	6	6	6
9	9	9	9	9	9	9
9	9	6	6	6	6	6
9	9	6	9	9	9	9
9	9	6	6	6	9	9
9	9	6	9	6	6	3
3	3	3	3	3	6	6
9	9	6	9	9	9	9
3	6	6	3	3	3	3
0	0	0	0	0	0	0
9	9	6	9	3	3	0
9	9	6	9	9	9	3
9	9	9	9	9	9	9
9	9	9	6	6	6	3
0	0	0	0	0	0	0
9	6	6	9	9	3	6
6	6	6	6	3	3	3
3	3	6	3	3	3	3
9	9	9	9	9	9	9
0	3	6	0	0	0	0
9	9	9	9	6	9	6
6	9	6	3	0	0	0
9	9	9	9	6	6	9
9	9	9	9	9	9	3
0	0	0	0	0	0	0

Providing Information	Parental Involvement	total
6	0	71
4	0	75
6	3	83
6	0	72
6	0	79
6	9	96
6	0	79
0	6	73
4	3	85
2	3	64
6	9	95
4	3	70
6	3	73
4	0	40
4	3	83
4	3	47
0	0	3
9	0	57
6	3	82
6	9	93
4	3	72
2	3	7
6	3	71
6	3	56
4	6	46
6	3	88
2	0	18
6	6	84
2	0	39
6	9	89
6	3	84
0	0	0

No	Kode	Introduction	Bridging	Asking adolescent to speak on their own	Psychosocial Screening	Open ended questions
1	B1R3	1	2	2	0	9
2	B3R3	3	1	2	1	3
3	B5R3	2	1	0	1	6
4	B7R3	1	2	1	2	9
5	B9R3	GUGUR				
6	B11R3	3	1	0	1	9
7	B13R3	2	0	0	0	3
8	B15R3	2	1	0	0	3
9	B17R3	1	1	2	3	6
10	B19R3	3	1	0	0	3
11	B21R3	3	1	2	0	6
12	B23R3	1	1	0	0	3
13	B25R3	3	2	0	3	9
14	B27R3	1	1	3	1	9
15	B29R3	2	1	0	2	9
16	B31R3	2	1	0	0	6
17	A1R3	2	0	0	0	0
18	A3R3	3	1	0	0	3
19	A5R3	0	1	2	0	6
20	A7R3	3	1	6	0	9
21	A9R3	3	1	0	1	9
22	A11R3	0	0	0	0	3
23	A13R3	1	2	0	3	6
24	A15R3	0	0	0	0	6
25	A17R3	0	0	0	0	9
26	A19R3	2	1	0	0	6
27	A21R3	3	1	2	1	6
28	A23R3	1	2	0	0	9
29	A25R3	3	0	0	0	0
30	A27R3	2	0	1	0	6
31	A29R3	3	1	0	3	9
32	A31R3	2	1	0	1	9
33	A33R3	0	0	0	0	0

Affirmation	Reflective Listening	Summary	Engaging	Focusing	Evoking	Planning
3	3	6	9	3	9	9
6	3	0	6	6	0	6
0	3	0	3	3	0	3
9	9	9	9	6	6	0
9	9	6	6	9	3	3
6	3	3	3	3	3	3
0	3	0	3	3	0	3
0	6	3	3	6	3	0
0	3	0	0	0	0	3
3	6	3	6	3	3	6
0	0	0	3	0	0	3
9	9	9	9	9	3	3
6	6	3	0	6	0	0
6	6	6	6	6	6	0
0	3	0	0	0	0	0
3	0	0	0	0	0	3
0	3	0	3	0	0	0
0	6	0	6	3	3	3
6	6	3	6	6	6	0
9	6	3	9	6	0	6
0	3	3	3	3	3	6
9	6	0	3	6	0	6
6	6	0	3	3	0	3
9	9	6	6	6	3	6
9	6	3	6	6	0	6
3	3	6	9	6	9	9
0	6	3	6	6	3	3
0	0	0	3	0	0	3
0	3	0	6	0	0	3
6	6	9	6	6	6	6
9	9	6	9	6	6	9
0	0	0	0	0	0	0

Providing Information	Parental Involvement	total
9	0	62
2	9	48
0	3	25
0	0	63
2	6	67
2	3	34
2	3	23
4	3	41
2	3	18
2	3	47
2	0	13
2	3	73
0	0	36
2	3	55
4	3	19
2	9	19
4	0	17
2	3	35
4	3	53
2	3	58
2	3	29
2	0	44
2	3	32
2	6	62
2	3	50
3	0	60
2	0	41
2	0	11
2	3	26
4	6	71
4	6	77
0	0	0

No	Kode	Introduction	Bridging	Asking adolescent to speak on their own	Psychosocial Screening	Open ended questions	Affirmation
1	B1R4	1	3	2	2	9	0
2	B3R4	1	2	2	3	9	3
3	B5R4	3	3	2	3	9	9
4	B7R4	belum dinilai					
5	B9R4						
6	B11R4	2	2	0	3	9	9
7	B13R4	belum dinilai					
8	B15R4	2	3	0	3	9	9
9	B17R4	3	3	2	3	9	9
10	B19R4	2	2	0	1	9	9
11	B21R4	3	2	2	3	9	6
12	B23R4	3	2	2	2	9	9
13	B25R4	3	3	0	3	9	9
14	B27R4	1	2	2	3	9	9
15	B29R4	2	3	0	3	6	9
16	B31R4	1	1	0	3	9	3
17	A1R4	1	1	0	1	3	6
18	A3R4	3	1	2	2	9	6
19	A5R4	3	1	2	3	9	3
20	A7R4	3	0	0	3	6	0
21	A9R4	0	2	0	3	9	9
22	A11R4	1	1	0	1	3	3
23	A13R4	3	2	1	3	9	9
24	A15R4	0	0	1	3	9	6
25	A17R4	1	2	1	3	9	9
26	A19R4	2	2	0	2	9	9
27	A21R4	3	1	1	2	9	6
28	A23R4	dialihkan B32R4					
29	A25R4	3	1	0	2	3	0
30	A27R4	GUGUR					
31	A29R4	3	2	1	3	9	6
32	A31R4	2	1	1	3	9	6
33	A33R4	0	0	0	0	0	0

Reflective Listening	Summary	Engaging	Focusing	Evoking	Planning	Providing Information
3	3	3	3	3	3	6
9	6	6	6	6	3	4
9	9	9	9	9	9	6
9	9	9	9	9	6	6
9	9	9	9	9	9	6
9	9	9	9	9	9	6
9	6	9	9	9	9	4
6	6	6	6	6	6	4
6	6	0	0	0	6	0
9	9	3	3	0	3	4
9	9	9	9	9	9	6
6	6	6	6	9	9	6
6	6	6	6	3	3	4
6	0	0	0	0	0	2
6	6	6	3	0	0	4
3	6	3	3	3	3	2
0	3	3	3	3	3	2
9	9	6	6	3	6	4
3	0	3	3	0	0	2
6	6	6	6	6	9	4
6	0	3	3	0	0	4
6	6	3	3	3	3	4
9	9	3	3	0	0	4
3	6	3	3	3	0	2
0	0	0	0	0	0	2
6	6	6	6	3	0	4
9	9	3	3	0	0	4
0	0	0	0	0	0	0

Parental Involvement	total
0	41
0	66
0	89
4	85
3	89
6	95
3	81
6	71
6	51
3	61
0	86
3	74
0	51
0	20
0	48
3	47
3	32
3	69
6	26
3	73
3	38
3	56
3	55
3	45
0	11
6	61
6	56
0	0

No	Kode	Introduction	Bridging	Asking adolescent to speak on their own	Psychosocial Screening	Open ended questions	Affirmation
1	B2R1	3	2	0	0	3	6
2	B4R1	3	2	2	0	6	9
3	B6R1	1	1	2	0	6	6
4	B8R1	1	2	3	1	9	9
5	B10R1	2	3	2	2	9	9
6	B12R1	1	2	1	3	9	3
7	B14R1	3	2	0	0	3	3
8	B16R1	1	1	1	0	0	0
9	B18R1	2	2	1	0	6	3
10	B20R1	1	2	1	1	6	9
11	B22R1	1	2	2	3	9	6
12	B24R1	2	2	1	3	6	9
13	B26R1	2	2	1	1	6	6
14	B28R1	1	1	1	0	6	0
15	B30R1	1	2	2	1	9	9
16	B32R1	2	2	2	3	9	6
17	A2R1	1	0	0	0	0	3
18	A4R1						
19	A6R1	3	2	1	0	6	6
20	A8R1	3	2	2	0	9	9
21	A10R1	3	2	1	0	6	6
22	A12R1	belum dinilai					
23	A14R1	1	1	0	1	6	3
24	A16R1	3	1	2	0	3	3
25	A18R1	belum dinilai					
26	A20R1	belum dinilai					
27	A22R1	1	1	0	0	3	0
28	A24R1	1	1	0	0	6	0
29	A26R1	belum dinilai					
30	A28R1	belum dinilai					
31	A30R1	1	1	1	3	9	9
32	A32R1						
33	A34R1	belum dinilai					

Reflective Listening	Summary	Engaging	Focusing	Evoking	Planning	Providing Information
6	3	6	3	0	3	2
6	6	6	0	0	0	0
6	6	9	6	0	9	2
9	0	9	9	9	9	6
6	6	9	6	6	6	4
6	3	6	3	3	3	4
3	3	3	0	0	3	2
0	0	0	0	0	0	2
3	0	3	6	3	6	4
9	6	6	6	3	3	2
3	3	6	6	3	3	2
6	6	9	6	3	3	4
9	6	6	6	6	3	4
3	0	0	0	0	0	0
6	0	6	6	6	6	2
6	9	6	6	9	9	6
0	0	0	0	0	0	2
6	0	3	0	3	3	2
6	3	6	6	0	6	4
6	3	6	6	0	3	2
3	0	3	3	0	3	2
0	0	0	0	0	3	2
0	0	0	0	0	0	2
0	0	3	0	0	0	0
9	6	6	6	9	6	2

Parental Involvement	total
3	40
3	43
3	57
0	76
6	76
3	50
6	31
6	11
3	42
9	64
3	52
9	69
6	64
0	12
0	56
3	78
0	6
6	41
0	56
6	50
3	29
6	23
3	10
3	14
0	68

No	Kode	Introduction	Bridging	Asking adolescent to speak on their own	Psychosocial Screening	Open ended questions	Affirmation
1	B2R2	3	2	0	2	6	9
2	B4R2	3	2	2	2	6	9
3	B6R2	3	2	0	2	3	9
4	B8R2	1	2	0	3	9	9
5	B10R2	1	3	0	2	9	9
6	B12R2	1	2	2	2	6	9
7	B14R2	3	2	0	2	6	6
8	B16R2	1	2	2	2	3	0
9	B18R2	1	2	3	2	9	9
10	B20R2	1	3	0	2	9	9
11	B22R2	1	2	2	2	6	9
12	B24R2	2	2	0	3	6	9
13	B26R2	2	2	0	3	9	9
14	B28R2	GUGUR					
15	B30R2	1	3	0	2	9	6
16	B32R2	1	2	2	2	6	9
17	A2R2	1	1	0	1	6	0
18	A4R2						
19	A6R2	3	3	0	2	6	9
20	A8R2	1	2	0	2	6	9
21	A10R2	3	2	0	3	9	9
22	A12R2	2	2	0	2	9	6
23	A14R2	3	3	0	2	9	3
24	A16R2	3	1	2	2	0	3
25	A18R2	1	2	0	2	3	0
26	A20R2	3	3	2	3	9	9
27	A22R2	2	2	0	2	3	3
28	A24R2	2	1	0	1	3	0
29	A26R2	1	2	0	2	6	6
30	A28R2	1	3	0	2	9	9
31	A30R2	1	2	0	2	9	6
32	A32R2						
33	A34R2	1	3	0	3	9	9

Reflective Listening	Summary	Engaging	Focusing	Evoking	Planning	Providing Information
9	9	9	9	6	9	4
9	6	9	9	9	9	4
3	6	3	3	3	9	2
9	6	9	9	9	9	2
9	6	9	9	6	9	2
6	9	3	6	3	6	2
3	3	0	3	0	3	2
3	6	3	3	3	3	2
9	9	9	9	9	9	6
9	9	9	6	9	9	2
9	6	9	9	6	9	4
6	6	6	6	3	6	2
9	9	9	9	6	9	2
9	9	9	9	3	9	2
3	6	3	6	6	6	2
3	0	3	0	0	0	0
9	6	9	9	6	9	2
9	9	3	3	9	9	2
9	9	9	6	3	9	4
9	6	9	9	6	9	2
6	6	3	6	3	9	3
0	3	3	0	0	3	2
3	0	0	3	0	3	2
9	6	9	9	9	9	2
3	6	3	3	3	3	2
0	0	0	0	0	0	2
9	6	3	3	3	3	4
9	6	9	9	6	9	4
6	6	9	9	6	6	6
9	9	9	6	6	6	2

Parental Involvement	total
6	83
6	85
3	51
0	77
0	74
3	60
3	36
0	33
6	92
0	77
0	74
3	60
6	84
0	71
3	57
0	15
6	79
0	64
6	81
6	77
6	61
0	22
3	22
6	88
3	38
3	12
0	48
0	76
0	66
0	72

No	Kode	Introduction	Bridging	Asking adolescent to speak on their own	Psychosocial Screening	Open ended questions	Affirmation
1	B2R3	1	2	2	2	3	0
2	B4R3	3	2	2	2	3	3
3	B6R3	3	2	2	3	6	3
4	B8R3	belum dinilai					
5	B10R3	3	2	2	3	6	9
6	B12R3	1	1	2	1	3	0
7	B14R3	1	1	0	0	0	0
8	B16R3	GUGUR					
9	B18R3	2	2	2	2	9	6
10	B20R3	3	2	2	3	6	0
11	B22R3	3	2	2	3	9	9
12	B24R3	3	2	2	3	6	6
13	B26R3	3	2	3	2	9	6
14	B28R3	GUGUR					
15	B30R3	2	3	2	2	6	6
16	B32R3	2	3	2	3	6	3
17	A2R3	1	0	2	3	3	0
18	A4R3						
19	A6R3	3	3	3	3	3	6
20	A8R3	3	2	2	2	6	0
21	A10R3	2	2	2	2	9	9
22	A12R3	2	1	2	3	6	3
23	A14R3	3	2	2	3	3	0
24	A16R3	0	2	2	3	3	3
25	A18R3	3	2	2	3	6	3
26	A20R3	3	3	2	3	9	3
27	A22R3	2	2	0	0	0	0
28	A24R3	2	3	3	2	6	3
29	A26R3	3	2	3	2	9	3
30	A28R3	3	2	2	3	6	0
31	A30R3	0	0	0	3	6	3
32	A32R3						
33	A34R3	1	2	2	2	9	9

Reflective Listening	Summary	Engaging	Focusing	Evoking	Planning	Providing Information
2	0	3	3	3	3	2
6	3	6	9	3	6	2
6	0	3	6	3	0	2
9	6	9	9	6	9	6
3	3	3	0	0	3	4
0	0	0	0	3	0	2
9	0	9	9	9	6	4
3	0	6	6	0	3	2
9	3	9	6	9	6	4
6	0	9	9	6	0	4
6	0	9	6	9	6	4
6	0	6	6	6	3	2
3	0	6	6	0	3	4
0	0	3	3	0	3	2
6	0	0	0	3	3	2
3	0	6	6	6	3	2
6	9	6	9	9	3	4
3	0	6	6	6	6	4
3	0	3	0	3	6	2
0	3	0	3	0	0	2
3	0	6	6	9	3	2
6	0	6	9	3	3	4
0	0	0	0	0	0	2
6	0	6	6	3	3	4
6	0	6	0	3	6	2
3	0	3	6	0	6	2
9	0	6	9	9	3	2
9	6	9	9	9	9	6

Parental Involvement	total
3	30
0	50
3	42
3	82
0	24
3	10
6	75
3	39
3	77
6	62
6	71
0	50
3	44
3	23
3	38
0	41
3	75
3	51
3	33
3	24
3	51
3	57
3	9
3	50
0	45
0	36
0	50
0	82

No	Kode	Introduction	Bridging	Asking adolescent to speak on their own	Psychosocial Screening	Open ended questions	Affirmation	Reflective Listening	Summary	Engaging
1	B2R4	1	2	0	2	9	6	9	9	9
2	B4R4	1	1	2	2	6	3	6	6	9
3	B6R4	1	1	2	1	6	9	6	0	6
4	B8R4	1	3	2	2	9	9	9	9	9
5	B10R4	1	1	0	2	6	6	3	0	6
6	B12R4	3	2	2	3	9	6	6	0	9
7	B14R4	2	2	0	2	9	6	6	0	6
8	B16R4	belum dinilai								
9	B18R4	2	3	2	1	9	6	6	6	9
10	B20R4	1	3	2	2	9	9	9	9	9
11	B22R4	3	3	3	2	6	6	6	3	6
12	B24R4	2	3	0	3	9	0	9	0	9
13	B26R4	1	2	2	2	9	6	9	9	9
14	B28R4	belum dinilai								
15	B30R4	1	3	2	1	3	6	6	9	6
16	B32R4	belum dinilai								
17	A2R4	1	1	0	1	3	6	3	0	0
18	A4R4									
19	A6R4	3	2	2	2	6	9	9	0	6
20	A8R4	1	0	2	0	6	9	6	3	6
21	A10R4	3	1	2	1	9	6	6	0	6
22	A12R4	3	0	2	2	6	9	6	0	9
23	A14R4	1	0	0	0	6	6	6	0	6
24	A16R4	3	2	2	0	3	9	6	6	3
25	A18R4	0	1	0	0	3	3	3	0	3
26	A20R4	1	2	2	0	9	9	9	0	9

27	A22R4	1	2	2	0	3	6	3	0	3
28	A24R4	1	1	0	0	3	0	3	0	0
29	A26R4	1	2	2	0	9	6	9	6	9
30	A28R4	3	3	2	2	6	6	6	6	9
31	A30R4	1	1	2	2	6	3	6	0	6
32	A32R4									
33	A34R4	3	2	2	3	6	0	6	6	6

Focusing	Evoking	Planning	Providing Information	Parental Involvement	total
9	6	9	2	6	79
6	6	6	2	0	56
6	0	6	0	6	50
9	9	9	2	0	82
6	6	6	0	3	46
6	9	9	0	3	67
6	6	3	2	3	53
9	6	9	6	6	80
9	9	9	0	0	80
6	6	6	2	3	61
9	6	9	2	6	67
9	9	9	2	0	78
6	6	3	0	0	52
6	3	6	0	3	33
6	6	3	2	3	59
0	0	6	2	0	41
6	0	6	2	2	51
6	3	6	2	3	57
6	0	3	2	3	39
3	0	0	0	3	40
0	3	3	0	3	22
9	6	6	0	6	68

0	0	0	0	3	23
0	0	0	4	3	15
9	6	6	2	3	70
6	3	3	2	6	63
6	6	0	0	0	39
6	6	6	2	0	54

DAFTAR KOMENTAR KELOMPOK A - Unggah Audio Kasus 1 - Konseling ke-1

NO	NAMA DEPAN	KOMENTAR
1	A1R1	Kekuatan konseling: Nakes sudah mencoba untuk melakukan teknik reflektif dan bertanya terbuka, sehingga mampu mendorong remaja untuk lebih banyak bercerita. Nakes juga sudah mencoba untuk memfokuskan area masalah remaja, yaitu berkaitan dengan pola makan remaja yang masih kurang sehat.<br><br>Ruang perbaikan: Saat melakukan konseling dengan orang tua, nakes cenderung banyak tertawa sehingga tidak sesuai dengan konteks pembicaraan yang sedang berlangsung.<br><br>Komentar: Nakes sudah cukup baik dalam melakukan konseling dengan remaja, namun terkesan canggung saat berkomunikasi dengan orang tua. Nakes sebaiknya lebih percaya diri dalam bertanya dan menggali informasi dari orang tua, sehingga konseling dapat berjalan lebih baik.
2	A2R1	-
3	A3R1	Kekuatan: Nakes sudah berusaha melakukan konseling dan bertemu orangtua. Sudah menggunakan pertanyaan terbuka dan ada probing yang dilakukan.<br><br>Ruang untuk perbaikan: Ada baiknya apabila Nakes meminta sesi berbicara sendiri dengan remaja, tanpa diinterupsi oleh orangtua, nanti setelah sesi berbicara dengan remaja, Nakes bisa ada sesi berbicara dengan orangtuanya. Selain itu, dalam melakukan asesmen/ screening, Nakes juga bisa menanyakan kepada remaja mengenai situasi di rumah, sekolah, dan stress yang dialaminya, sehingga tidak hanya sebatas menanyakan pola makan dan aktivitas saja. Apabila ada isu mengenai berat badan, situasi remaja di rumah, sekolah, dan stress bisa mempengaruhi isu berat badannya. Misalnya saja, seorang remaja yang jadwal sekolahnya terlalu padat dan memiliki banyak tugas, mungkin makannya tidak akan teratur ataupun sekali makan langsung banyak dan sedikit aktivitas. Contoh lain terkait dengan stress, apabila remaja merasa tertekan, emosinya tak stabil, ia berusaha melampiaskan emosi dengan banyak makan atau sebaliknya remaja mengalami stress, dia sering mengurung diri di kamar, sering bersedih, tidak tidur dan tidak mau makan. Penting bagi Nakes untuk mengetahui situasi remaja ini secara menyeluruh, tidak hanya sebatas pola makan dna aktivitasnya, tetapi juga situasi di rumah, sekolah, dan stressnya. Dengan demikian Nakes akan bisa mengajak remaja untuk mendiskusikan hal-hal yang bisa dilakukan untuk mengatasi penyebab terganggunya pola makan atau terhambatnya aktivitas remaja yang menjadi sumber isu berat badannya.<br><br>Komentar secara keseluruhan: Konseling yang dilakukan Nakes bisa ditingkatkan lagi dan sepertinya reakaman yang di-summit terputus/ terhenti di awal-awal Nakes melakukan konseling. Mungkin untuk selanjutnya, apabila diminta summit rekaman konseling lagi, Nakes bisa mengecek apakah rekamannya sudah sesuai dari awal konseling hingga selesai atau ternyata terputus di awal maupun tengah sesi konseling.

4	A4R1	Kekuatan konseling: Nakes sudah bertanya mengenai kondisi badan, pola aktivitas, dan pola makan remaja.<br><br>Ruang perbaikan: Rekaman berjalan terlalu singkat, sehingga informasi yang didapatkan tidak lengkap dan nakes tidak melakukan teknik konseling dalam rekaman tersebut.<br><br>Komentar: Berdasarkan hasil rekaman, nakes tidak melakukan sesi konseling sama sekali, melainkan melakukan monolog seolah-olah sedang melakukan sesi konseling dengan remaja. Hal ini membuat data/informasi dari rekaman menjadi kurang valid untuk dinilai sebagai sesi konseling secara utuh.
5	A5R1	Kekuatan: Nakes sudah lancar dalam melakukan konseling, sudah menggunakan pertanyaan terbuka dan probing untuk membuat remaja bercerita, alur konseling juga terjaga secara konsisten dan fokus kepada isu kelebihan berat badan atau obesitas remaja. Sudah ada afirmasi dan mendengarkan reflekstif yang dilakukan oleh Nakes.<br><br>Ruang perbaikan: Ada baiknya apabila di awal konseling Nakes meminta sesi untuk berbicara sendiri dengan remaja terlebih dahulu dan barulah setelahnya orangtua bisa ikut sesi konseling. Hal ini untuk membuat remaja merasa lebih nyaman sehingga akan bisa lebih terbuka mengenai isu/ permasalahan yang dihadapinya yang mungkin terkait dengan isu kelebihan berat badan atau obesitas. Hal ini dikarenakan pada beberapa remaja, ada hal-hal yang masih belum bisa diungkapkannya secara terbuka di hadapan orangtuanya dan hal ini dikhawatrirkan akan berpengaruh terhadap sesi konseling. Selain itu, Nakes juga bisa melakukan skrining atau mengeksplorasi sedikit mengenai situasi remaja di rumah, sekolah/ kelas online, serta stress yang mungkin dialami oleh remaja, hal ini karena faktor-faktor psikosial itu mungkin saja mempengaruhi isu kelebihan berat badan atau obesitas pada remaja. Saat melakukan konseling, Nakes juga bisa lebih menggunakan teknik evoking, yaitu membantu remaja mnenyadari bahwa isu kelebihan berat badan atau obesitas penting untuk diatasi dan remajajuga perlu berupaya untuk mengatasinya secara aktif, sementara Nakes dan orangtua adalah support system yang bisa membantu remaja, namun remaja tetap harus berusaha menerapkan perubahan-perubahan yang telah direncanakan atau dibahas saat sesi konseling. Terakhir, ketika akan melibatkan orangtua untuk memberikan dukungan atau tuntutan kepada remaja untuk menjalankan rencana perubahan yang sudah disepatinya dalam konseling, Nakes bisa mendiskusikan dengan orangtua kira-kira bentuk konkrit dari dukungan dan tuntutan yang bisa diberikan orangtua kepada remaja di rumah apa saja., misalnya orangtua bisa mendukung dengan lebih menyediakan buah-buahan yang disukai remaja dan mengurangi memasak gorengan, sementara tuntutan yang bisa diberikan orangtua kepada remaja di rumah seperti mendorong remaja untuk aktif secara fisik, seperti mengawasi dan mengingatkan remaja berolahraga secara teratur minimal 2 kali seminggu selama 30 menit atau membantu orangtua dalam aktivitas fisik/ aktivitas yang memerlukan banyak gerakkan.<br><br>Komentar secara keseluruhan: Konseling remaja yang dilakukan Nakes masih bisa ditingkatkan lagi. Nakes memiliki potensi yang baik ketika berbicara dengan remaja,

		terbukti di dalam rekaman sesi konseling terasa lancar serta Nakes maupun remaja juga bisa engage dalam konseling.
6	A6R1	Kekuatan konseling: Selama proses skrining dan penggalian data, nakes secara aktif bertanya secara bergantian antara remaja dengan orang tua, sehingga informasi yang didapatkan menjadi kaya dan dapat dikonfirmasi terlebih dahulu kebenarannya. Selain itu, nakes juga sudah berusaha melakukan teknik planning, dengan bertanya kepada remaja mengenai rencana perubahan perilaku yang akan dilakukan setelah sesi konseling.<br><br>Ruang perbaikan: Karena nakes tidak melakukan teknik evoking dan langsung melakukan teknik planning, sehingga remaja merasa tidak perlu melakukan perubahan perilaku. Akan lebih baik jika nakes terlebih dahulu melakukan teknik evoking (bertanya mengenai niat dan motivasi remaja untuk berubah), sebelum bertanya rencana perubahan perubahan perilaku yang akan dilakukan.<br><br>Komentar: Secara keseluruhan, sesi konseling yang dilakukan sudah cukup baik. Untuk kedepannya, akan lebih baik jika nakes menggunakan teknik MI seperti engaging, focusing, evoking, dan planning secara lengkap agar remaja lebih termotivasi untuk mulai menatakelola pola hidupnya.
7	A7R1	Kekuatan: Nakes sudah berusaha untuk mempraktekkan konseling dengan remaja. Nakes terdengar ramah dan juga bisa playful saat sesi konseling dengan remaja. Selain itu sudah banyak pertanyaan terbuka yang diberikan.<br><br>Ruang untuk perbaikan: Ada baiknya apabila Nakes meminta sesi remaja untuk berbicara sendiri tanda diinterupsi oleh orangtua, atau saat sesi Nakes boleh untuk meminta/ menjelaskan kepada orangtua bahwa remaja bisa menjawab sendiri, dan nanti akan ada sesi dimana Nakes akan berdiskusi dengan orangtua. Dengan demikian remaja akan bisa lebih terlibat aktif dalam konseling serta bisa terbuka lebih banyak akan isu-isu terkait yang mempengaruhi kelebihan berat badan atau obesitasnya. Dalam pemberian informasi kepada remaja, Nakes bisa menggunakan teknik Tanya-Beri-Tanya (TBT), misal Nakes bisa menanyakan dahulu apa yang remaja ketahui mengenai gizi seimbang, kemudian memberikan wkatu kepada remaja untuk menceritakan yang ia ketahui, baru selanjutnya Nakes melengkapi informasi tersebut dan menanyakan lagi kepada remaja mengenai hal apa yang membuatnya belum bisa menjalankan gizi seimbang tersebut. Dengan cara ini Nakes tidak hanya memberikan informasi tetapi bisa mengajak remaja untuk membangun kesadaran akan kendala atau halangannya untuk bisa menjalankan pola makan bergizi seimbang. Setelah remaja sadar akan kendalanya bisa dilanjut ke penyusunan rencana/ planning untuk mengatasi kendalan-kendala tersebut. Tentunya rencana perubahan perilaku yang papling baik adalah yang lebih banyak muncul dari remaja itu sendiri dan Nakes berperan mengarahkan remaja memunculkan ide-ide terkait perubahan perilaku, sehingga disarankan agar Nakes tidak langsung memberikan saran tindakan, tetapi ajak remaja untuk mendiskusikannya bersama. Pada akhir sesi, Nakes bisa menutup sesi dengan memberikan rangkuman mengenai poin-poin penting yang dibahas selama sesi konseling dan konfirmasi kembali ke remaja, apakah ada hal yang ingin ditambahkannya. Hal ini untuk melihat apakah informasi yang Nakes tangkap dari remaja selama konseling berlangsung sudah tepat, dan apabila belum tepat, remaja

		sendiri yang akan mengoreksi maupun membetulkannya.<br><br>Komentar secara keseluruhan: Konseling yang dilakukan Nakes bisa ditingkatkan lagi, dengan meminta waktu berbicara hanya dengan remaja, mengunakan teknik Tanya-Beri-Tanya (TBT) dalam menyampaikan informasi, serta lebih melibatkan remaja dalam membuat rencana perubahan perilaku, serta memberikan kesimpulan/ rangkuman di akhir sesi.
8	A8R1	-
9	A9R1	Kekuatan: Nakes terdengar semangat dan antusias dalam mempraktikkan konseling remaja, bahkan hingga mengirim 2 rekaman dengan remaja yang berbeda.<br><br>Ruang perbaikan: Saat melakukan konseling, perlu dibedakan dengan melakukan sosialisasi. Konseling pada dasarnya memiliki struktur dan tujuan/ goal yang ingin dicapai dalam setiap sesinya, sehingga membatasi ruang lingkup pembahasan dalam konseling akan lebih membantu remaja dalam memetakan permasalahan yang dihadapinya, menimbulkan kesadaran remaja untuk melakukan perubahan, dan memotivasi remaja untuk membahas atau menyusun rencana tindakkan yang akan dilakukannya dalam rangka berusaha mengatasi permasalahan yang sedang dihadapi. Tentunya dalam melakukan konseling remaja, Nakes bisa lebih banyak mengajak remaja untuk terbuka dan bercerita tentang dirinya, kehidupannya, dan permasalahan yang menjadi fokus konseling. Nakes bisa lebih menggunakan teknik-teknik mendnegarkan aktif dalam konseling, seperti memberikan pertanyaan terbuka, probing, memberikan afirmasi tentang upaya-upaya positif dan kekuatan yang dimiliki remaja untuk mengatasi masalahnya, melakukan refleksi/ memparafrasekan pernyataan atau hal yang disampaikan remaja kemudian menayakan kembali ke remaja mengenai hal apa yang mungkin dilakukannya untuk mengatasi kesulitan/ masalah yang dialami. Dalam konseling, saran-saran pun tidak harus selalu diberikan oleh Nakes secara langsung, namun Nakes mengarahkan remaja dengan teknik-teknik konseling ataupun MI untuk menemukan saran-saran atau rencana perubahan yang paling pas dengan diri dan kondisinya saat ini. Saran yang terbaik adalah saran yang lebih banyak muncul dari remaja itu sendiri dan Nakes membantu mengarahkan remaja hingga remaja bisa menemukan saran bagi dirinya sendiri.<br><br>Komentar secara keseluruhan: Konseling remaja yang dilakukan oleh Nakes masih bisa ditingkatkan lagi. Nakes memiliki semngat untuk belajar dan mempraktikkan konseling, sehingga dengan terus berlatih dan meminta feedback kepada kolega atau remaja mengenai bagaimana cara agar sesi konseling bisa membantu remaja, maka Nakes pun akan semakin lancar dan terstruktur dalam melakukan konseling.
10	A10R1	-
11	A11R1	Kekuatan: Nakes sudah menggunakan pertanyaan terbuka dan probing dengan baik sehingga remaja bisa terbuka serta bercerita tentang diri dan situasi terkait dengan kelebihan berat badan. Selain itu, Nakes juga meminta sesi remaja berbicara sendiri tanpa orangtua. Nakes pun sudah tepat dalam memberikan afirmasi atas hal-hal positif yang dilakukan remaja demi menurunkan berat badannya.<br><br>Ruang perbaikan: Dalam hal fokusing atau pemetaan masalah, ada baiknya Nakes mendiskusikan dan memberikan opsi kepada remaja, misalkan setelah remaja

		bercerita diketahui bahwa komposisi gizi dalam makanan remaja kurang seimbang dan aktivitas fisiknya juga tidak begitu banyak. Disini Nakes bisa mengundang remaja, dari kedua hal itu yang mana dulu yang ingin dikelola, ataukan ingin mengelola dua-duanya sekaligus. Kemudian dalam perencanaan/ planning perubahan Nakes bisa lebih melibatkan remaja, sehingga rencana perubahan yang muncul akan lebih banyak diungkapkan atau datang dari remaja itu sendiri. Penting juga bagi Nakes untuk membuat remaja sadar bahwa masalah berat badannya hanya bisa diselesaikan oleh dirinya dan Nakes hanya mendampingi prosesnya. Kemudian untuk skrining psikososial, Nakes bisa lebih menggali cerita remaja terkait seperti apa situasi di rumah, sekolah/ pendidikan, dan stress yang mungkin dialami remaja, yang mungkin berpengaruh terhadap masalah berat badan remaja. Terakhir, Nakes perlu melibatkan orangtua, jadi setelah selesai konseling dengan remaja, Nakes bisa mengajak ornagtua untuk membahas bentuk-bentuk dukungan dan tuntutan yang bisa diberikan orangtua ke anaknya demi membantu anak menurunkan berat badan, misal orangtua bisa menyediakan buah-buahan di rumah, dalam memasak tidak melulu harus digoreng, dan orangtua juga bisa memantau atau mengingatkan anaknya untuk meningkatkan aktivitas fisik/ olahraga.<br><br>Komentar secara keseluruhan: Nakes sudah melakukan konseling remaja dengan cukup baik dan masih ada beberapa hal yang bisa ditingkatkan lagi. Nakes sudah sangat lancar dalam memberikan konseling kepada remaja dan sudah bisa membuat remaja nyaman untuk bercerita/ sharing tentang kondisinya, sehingga sesi konseling pun terasa engaging.
12	A12R1	Kekuatan konseling: Nakes sudah memastikan setiap bagian informed consent agar terisi dan terjawab dengan baik. Nakes juga melakukan skrining terhadap remaja maupun orang tua secara terstruktur dan menyeluruh, sehingga area masalah dapat lebih terpetakan dengan baik.<br><br>Ruang perbaikan: Nakes tidak menyediakan waktu untuk remaja bicara sendiri, sehingga beberapa pertanyaan cenderung dijawab oleh orang tua meskipun sesungguhnya remaja yang sedang ditanyakan nakes. Remaja juga jadi berpotensi kurang bebas/jujur dalam menjawab karena keberadaan orang tua saat ditanya hal-hal sensitif.<br><br>Komentar: Secara keseluruhan, sesi terkesan hanya berfokus untuk melakukan skrining awal, sehingga belum melakukan teknik-teknik konseling yang dimaksudkan untuk melakukan tata kelola obesitas. Meskipun begitu, skrining yang dilakukan menyeluruh menjadi langkah awal yang baik untuk mengidentifikasi langkah selanjutnya dalam penanganan masalah obesitas remaja.
13	A13R1	Kekuatan konseling: Nakes sangat informatif dalam memberikan informasi, saran, dan masukan kepada remaja serta orang tua remaja, dan memastikan pemahaman orang tua serta remaja terhadap informasi tersebut. Nakes juga secara konsisten memberikan motivasi kepada remaja untuk mau berubah dan mulai mengatur pola aktivitas dan makannya.<br><br>Ruang perbaikan: Sebaiknya rencana perubahan perilaku disampaikan oleh remaja, dan bukan dari nakes. Hal ini dimaksudkan agar remaja sendiri yang menentukan

		kadar kemampuannya dalam mulai melakukan perubahan pola aktivitas dan pola makan.<br><br>Komentar: Secara umum, sesi konseling berjalan cukup terstruktur dengan adanya proses skrining yang menyeluruh dan pemberian informasi yang lengkap. Hanya saja, sesi konseling berpotensi dapat berjalan lebih baik jika nakes menggunakan teknik-teknik MI selama sesi konseling berlangsung
14	A14R1	Kekuatan: Nakes sudah berusaha bertemu dengan orangtua dan memberikan pernyataan terkait informed consent dan menjelaskan sesi yang akan dilakukan.<br><br>Ruang untuk perbaikan: Sepertinya sesi yang dioberikan baru hanya meminta kesediaan orangtua agar anaknya bisa terlibat dalam konseling, meminta pengisian informed consent, dan perkenalan dari Nakes yang akan memberikan sesi konseling. Mungkin baiknya di sesi konseling, Nakes bisa meminta sesi untuk berbicara langsung dengan remaja tanpa diinteruspsi oleh orangtuanya. Coba perbanyak penggunaan pertanyaan terbuka, probing, afirmasi, evoking dan dalam memberikan informasi bisa menggunakan teknik Tanya-Beri-Tanya (TBT). Dalam hal planning ketika sesi konseling, Nakes bisa memberikan ruang yang lebih besar pada keterlibatan remaja dalam mendiskusikan dan menyusun rencana perubahan perilaku, karena dengan demikian remaja akan merasa ikut memiliki tanggung jawab atas masalah kelebihan berat badan atau obesitasnya.<br><br>Komentar secara keseluruhan: Konseling yang dilakukan Nakes bisa ditingkatkan lagi dan sepertinya rekaman yang di-summit baru sesi perkenalan dan informed consent.
15	A15R1	Kekuatan konseling: Nakes sangat baik dalam melakukan evoking, seperti bertanya cita-cita yang dimiliki remaja. Hal ini membuat remaja lebih termotivasi untuk mulai melakukan perubahan pola hidup. Selain itu, nakes juga memiliki pengetahuan yang luas sehingga mampu memberikan informasi yang tepat guna untuk remaja.<br><br>Ruang perbaikan: Sebaiknya rencana perubahan datang dari remaja, bukan disampaikan oleh nakes. Hal ini untuk mencegah agar remaja tidak merasa sedang disuruh dan menjadi kurang termotivasi untuk berubah.<br><br>Komentar: Secara umum, nakes sudah baik dalam membangun motivasi remaja selama sesi konseling, serta dalam melibatkan orang tua selama konseling berlangsung. Akan lebih baik jika nakes juga mendiskusikan peran orang tua secara lebih mendalam, misalnya peran dukungan (seperti peran orang tua dalam memasak menu sehat di rumah), maupun peran tuntutan (dalam mengingatkan remaja terkait cita-citanya menjadi polisi).
16	A16R1	Kekuatan konseling: Di bagian awal sesi konseling, nakes sudah sangat baik dalam melakukan perkenalan dan pemaparan mengenai tujuan sesi konseling. Selain itu, nakes juga sudah secara terstruktur melakukan skrining terhadap berbagai area masalah yang dihadapi remaja.<br><br>Ruang perbaikan: Saat memberikan informasi, akan lebih baik jika nakes bertanya terlebih dahulu mengenai pemahaman remaja dan orang tua terhadap informasi yang akan disampaikan (menggunakan teknik TBT, tanya-beri-tanya), karena saat

		sesi, nakes cenderung langsung memberikan informasi tanpa konfirmasi terlebih dahulu.<br><br>Komentar: Secara keseluruhan, konseling berjalan sudah cukup baik. Nakes juga sudah melakukan teknik evoking untuk memastikan kesiapan remaja dalam melakukan remaja. Namun, konseling yang berjalan ringkas dan cukup padat dengan proses skrining membuat tahapan-tahapan MI maupun teknik komunikasi dasar lainnya menjadi belum dilakukan oleh nakes kepada remaja dan orang tua.
17	A17R1	-
18	A18R1	Kekuatan: Nakes sudah berusaha untuk mempraktekkan konseling dengan remaja dan melibatkan peran orangtua dalam mengajak remaja berolahraga/ meningkatkan aktivitas bersama. Informasi yang diberikan pun jelas.<br><br>Ruang untuk perbaikan: Sesi konseling akan lebih baik apab ila dibiarkan mengalir, Nakes bisa memberikan pertanyaan satu per satu kepada remaja, setiap satu pertanyaan remaja langsung merespon sehingga Nakes pun nantinya bisa merespon balik jawaban dari remaja dengan mengeksplorasi jawaban itu menggunakan probing serta menanyakan pertanyaan terbuka lainnya yang bertujuan memberikan kesempatan remaja untuk terbuka dan cerita lebih banyak mengenai dirinya. Konseling akan jadi lebih baik apabila antara Nakes dan remaja terjadi komunikasi 2 arah yang timbal balik dan bertujuan mengarahkan remaja ke perubahan perilaku terkait isu berat badan yang dialami, entah itu mengubah pola makan maupun meningkatkan aktivitas fisik.<br><br>Komentar secara keseluruhan: Konseling yang dilakukan Nakes bisa ditingkatkan lagi, dengan menggunakan pola interaksi dua arah dengan remaja, jadi satu pertanyaan dari Nakes bisa langsung direspon oleh remaja, tidak harus menunggu pertanyaan yang diberikan banyak baru direspon.
19	A19R1	-
20	A20R1	Kekuatan konseling: Nakes meminta consent (kesediaan) dari remaja maupun orang tua terlebih dahulu, agar memastikan komitmen dari remaja dalam mengikuti konseling. Nakes juga sudah baik dalam konseling karena berfokus pada remaja, sehingga lebih banyak berkomunikasi dengan remaja dibandingkan orang tua.<br><br>Ruang perbaikan: Nakes cenderung bertanya dan menjawab pertanyaan tersebut sendiri, sehingga kurang memberikan kesempatan bagi remaja untuk menjawab maupun mengelaborasi jawabannya.<br><br>Komentar: Sesi konseling yang berjalan sangat singkat membuat tahapan pada MI menjadi tidak dilakukan oleh nakes. Di kemudian hari, akan lebih baik jika nakes mengalokasikan waktu lebih banyak untuk melaksanakan konseling.
21	A21R1	-
22	A22R1	Kekuatan konseling: Nakes sudah cukup baik dalam memberikan informasi, karena informasi yang diberikan cukup lengkap dan menyeluruh. Nakes juga memastikan terlebih dahulu pemahaman remaja terhadap informasi yang diberikan, sebelum menutup sesi konseling.

		Ruang perbaikan: Sesi konseling berjalan sangat kaku, sehingga sebaiknya nakes lebih berusaha untuk membangun rapport dengan remaja.<br><br>Komentar: Secara keseluruhan, kesan yang terdengar dari sesi konseling ini cenderung seperti tidak alami (scripted), hal ini membuat percakapan yang terjadi menjadi sangat kaku dan sangat terpaku oleh daftar pertanyaan dan informasi yang harus disampaikan oleh nakes kepada remaja. Di kemudian hari, akan lebih baik jika nakes lebih percaya diri untuk bertanya dan memberikan informasi secara spontan saja, dan tidak terpaku oleh aturan baku atau informasi yang tertulis pada leaflet.
23	A23R1	Kekuatan konseling: Nakes sudah sangat baik dalam melakukan skrining, berbagai pertanyaan yang diberikan sudah cukup spesifik dan sesuai untuk menggali permasalahan yang dihadapi remaja. Selain itu, nakes juga sudah sangat baik dalam membangun rapport dengan remaja, sehingga remaja nyaman untuk bercerita sepanjang sesi berlangsung.<br><br>Ruang perbaikan: Saat memberikan informasi, akan lebih baik jika nakes menggunakan teknik TBT (tanya-beri-tanya), yaitu bertanya pengetahuan remaja terlebih dahulu terhadap info yang akan disampaikan, kemudian bertanya kembali pendapat remaja atas info tersebut.<br><br>Komentar: Di dalam sesi konseling, nakes sudah melakukan beberapa teknik MI seperti engaging, focusing, dan evoking. Hal ini terlihat dari usaha nakes untuk bertanya perihal kepercayaan diri remaja, dan masalah body image yang mungkin mendasari remaja untuk ingin mengelola berat badannya. Akan lebih baik jika nakes juga melakukan teknik planning, yaitu membiarkan remaja untuk mengungkapkan rencana perubahannya sendiri kepada nakes.
24	A24R1	Kekuatan: Nakes menjalankan sesi konseling remaja dengan sangat lancar dan mengalir. Penggunaan pertanyaan terbuka dan probing yang baik sehingga berhasil membuat remaja bercerita lebih banyak tentang kondisinya, ada upaya evoking yang dilakukan konsisten serta mengarahkan pada perubahan perilaku. Nakes juga lancar dalam menerapkan teknik refleksi dan afirmasi, serta melakukan skrining psikososial remaja secara lengkap (situasi di rumah, sekolah, dan potensi stress).<br><br>Ruang perbaikan: Oleh karena ini sesi dengan remaja dan terkait pola makan, perlu ada sesi juga dengan orangtua, untuk membahas mengenai dukungan dan tuntutan yang bisa diberikan oleh orangtua kepada remaja dalam upaya menurunkan berat badan berlebih dari remaja. Selain itu, untuk planning juga bisa diringkatkan lagi, misalnya dengan menayakan ke remaja, kira-kira ada hal apa saja yang bisa dilakukan remaja agar kondisi tubuhnya fit atau tidak kelebihan berat badan. Kemudian Nakes juga bisa memberikan informasi atau edukasi ke remaja dengan menggunakan teknik Tanya-Beri-Tanya (TBT).<br><br>Komentar secara keseluruhan: Konseling yang dilakukan Nakes sudah baik dan masih bisa ditingkatkan lagi. Nakes dengan jelas, lugas, dan playful sudah bisa membangun komunikasi yang baik serta intens dengan remaja, sehingga sepanjang sesi konseling, terasa sangat mengalir dan remaja pun cukup bisa untuk bercerita
25	A25R1	Kekuatan konseling: Nakes sudah baik dalam melakukan skrining terhadap pola makan dan jenis makanan yang dikonsumsi oleh remaja. Nakes juga secara aktif

		bertanya kepada orang tua untuk memastikan keterlibatan orang tua dalam mengatur makanan remaja di rumah.<br><br>Ruang perbaikan: Sesi konseling masih terkesan interogatif, sehingga percakapan berjalan satu arah dan cenderung monoton. Akan lebih baik jika remaja lebih aktif dilibatkan untuk menentukan sendiri perubahan pola makan dan aktivitasnya menggunakan teknik MI.<br><br>Komentar: Sesi konseling belum menggunakan teknik MI, sehingga perubahan aktivitas dan pola makan belum direncanakan secara maksimal. Penggunaan teknik MI disarankan agar remaja lebih terlibat dan termotivasi untuk mulai menatakelola obesitasnya.
26	A26R1	Kekuatan konseling: Nakes cukup baik dalam membangun rapport dengan remaja maupun orang tua, sehingga sesi konseling memfasilitasi remaja untuk lebih terbuka. Beberapa teknik konseling seperti teknik reflektif dan bertanya terbuka juga sudah baik dilakukan oleh nakes selama sesi.<br><br>Ruang perbaikan: Terdapat beberapa momen yang bisa dimanfaatkan oleh nakes untuk mengeksplorasi area masalah remaja lebih lanjut, seperti saat membahas makanan kesukaan atau alasan mengapa remaja menyukai makanan viral namun tinggi kandungan gula/karbohidrat.<br><br>Komentar: Nakes secara umum sudah baik dalam melakukan konseling, namun akan lebih baik jika nakes lebih aktif menggali informasi perihal kekuatan remaja (seperti saat membahas remaja yang menyukai sayur), maupun masalah pola makan dan aktivitas yang dihadapi oleh remaja.
27	A27R1	Kekuatan konseling: Nakes sudah sangat baik dalam melakukan skrining terhadap area masalah yang dihadapi remaja, sehingga gaya hidup remaja dapat tergambarkan dengan baik. Nakes juga sudah baik dalam melakukan teknik bertanya terbuka dan teknik reflektif, sehingga baik orang tua maupun remaja sangat terbuka dalam konseling.<br><br>Ruang perbaikan: Nakes cenderung kesulitan dalam mengelola jalannya konseling agar tidak didominasi oleh orang tua remaja. Akan lebih baik jika nakes lebih banyak memberikan kesempatan untuk remaja menjawab, atau membagi sesi konseling menjadi 2 sesi di mana salah satunya remaja diminta untuk berbicara sendiri.<br><br>Komentar: Secara umum, nakes sudah mampu dalam melakukan beberapa teknik komunikasi dasar dalam konseling. Akan lebih baik jika nakes melakukan teknik MI seperti evoking, khususnya saat remaja menyampaikan bahwa ia bercita-cita menjadi polwan, agar remaja lebih termotivasi untuk memulai perubahan dalam gaya hidup.
28	A28R1	Kekuatan: Sudah mencoba melakukan konseling pada remaja hingga sesi kedua.<br><br>Ruang untuk perbaikan: Perlu memberikan waktu bagi anak untuk bercerita tentang kondisinya saat sesi berlangsung, Nakes bisa lebih banyak menggunakan pertanyaan terbuka dan probing (pertanyaan lanjutan untuk memperdalam cerita), memberikan afirmasi, serta merefleksikan cerita dari remaja.

		Komentar secara keseluruhan: Konseling sangat bisa untuk ditingkatkan lagi. Nakes sudah memiliki kemauan untuk mencoba menggunakan metode atau teknik konseling, hal ini perlu dilatih terus - menerus dan izinkan remaja untuk lebih banyak bercerita tentang kondisi dan permasalahannya, Nakes bisa menggali atau memunculkan cerita dari remaja dengan memperbanyak penggunaan pertanyaan terbuka, memberikan afirmasi atas hal yang disampaikan remaja, serta bisa juga merefleksikan kembali cerita sambil memberikan probing (pertanyaan untuk memperdalam cerita).
29	A29R1	-
30	A30R1	Kekuatan konseling: Nakes menggali pola hidup dari remaja secara menyeluruh, sehingga tergambarkan kegiatan remaja sehari-hari dari pagi hingga malam hari. Nakes juga memberikan informasi yang tepat dan lengkap kepada remaja, sehingga informasi tersebut bermanfaat untuk memotivasi remaja dalam mengubah pola makan dan aktivitasnya.<br><br>Ruang perbaikan: Proses konseling berjalan seperti scripted, sehingga tidak natural dan kurang menggambarkan dinamika komunikasi dalam konseling yang sesungguhnya. Untuk kemudian hari, sebaiknya nakes melakukan konseling apa adanya sesuai realita (tidak dibuat skrip) agar konseling yang dilakukan bermanfaat untuk remaja.<br><br>Komentar: Secara umum, proses konseling belum menggunakan teknik-teknik dalam MI. Meskipun begitu, nakes tetap mampu menggunakan beberapa teknik komunikasi dasar dalam konseling seperti bertanya terbuka maupun memberikan rangkuman di dalam percakapan.
31	A31R1	-
32	A32R1	Kekuatan konseling: Nakes sudah berusaha untuk lebih aktif bertanya kepada remaja dan memberikan kesempatan remaja untuk menjawab, karena percakapan cenderung didominasi oleh jawaban dari orang tua. Selama sesi konseling, nakes juga secara aktif mendengarkan dan melakukan teknik engaging dan focusing untuk melihat akar masalah yang dihadapi remaja.<br><br>Ruang perbaikan: Sebaiknya nakes lebih membahas motivasi remaja untuk melakukan perubahan melalui teknik evoking, agar remaja lebih terdorong untuk mengurangi konsumsi gula dan karbohidrat.<br><br>Komentar: Secara umum konseling sudah berjalan baik, nakes secara aktif mengidentifikasi area masalah remaja dan memberikan afirmasi terhadap kekuatan yang dimiliki remaja. Untuk kedepannya, akan lebih baik jika remaja lebih dilibatkan dalam perencanaan perubahan perilakunya.
33	A33R1	Kekuatan konseling: Nakes sudah baik karena menyampaikan di awal bahwa proses konseling akan lebih banyak berfokus/bertanya kepada remaja, dibandingkan kepada orang tua. Hal ini membantu agar orang tua tidak mendominasi jawaban selama sesi berjalan. Selain itu, nakes juga sudah cukup informatif dalam memberikan saran terkait gizi, perubahan pola makan, dan saran kepada orang tua untuk melibatkan remaja dalam aktivitas rumah agar remaja lebih aktif.

		Ruang perbaikan: Nakes belum mengeksplorasi atau melakukan skrining terhadap faktor-faktor selain pola makan dan aktivitas, seperti faktor stres. Di kemudian hari, akan lebih baik jika nakes juga menggali informasi terkait faktor stres/psikologis yang mungkin berkontribusi terhadap pola hidup dan aktivitas remaja.<br><br>Komentar: Secara umum, sesi sudah berjalan cukup baik, namun dapat dikembangkan menjadi lebih baik lagi jika nakes melakukan teknik-teknik komunikasi seperti memberikan afirmasi kepada remaja, mendengarkan reflektif, maupun teknik-teknik MI (motivational interviewing).

DAFTAR KOMENTAR **KELOMPOK B - Unggah Audio Kasus 1 - Pra Pelatihan**

No	Kode Peserta	Komentar
1	B1R1	Kekuatan konseling: Nakes melakukan intro dengan baik, berusaha untuk building rapport dengan remaja maupun keluarga yang mendampingi remaja (ibu dan kakak).<br><br>Ruang perbaikan: Sepanjang sesi konseling, nakes cenderung tidak menggunakan teknik MI sehingga terkesan interogatif dan hanya memberikan saran. Nakes juga kurang memaksimalkan potensi keberadaan keluarga remaja yang sesungguhnya dapat berperan penting terhadap perubahan perilaku remaja.<br><br>Komentar: Secara umum, teknik-teknik konseling yang digunakan belum mengarah pada MI sehingga belum terlihat rencana perubahan perilaku yang bermanfaat untuk menangani masalah obesitas pada remaja.<br><br>Tamabahan kekuatan konseling: Nakes juga melibatkan keberadaan orang tua dan kakak remaja, sehingga pemberian informasi kepada remaja juga diterima dan dipahami oleh anggota keluarga remaja. Selama penggalian data terkait area masalah, nakes bertanya secara cukup mendalam sehingga dapat tergambar kebiasaan remaja, khususnya di rumah.
2	B2R1	Kekuatan: Nakes terdengar ramah dan ada upaya untuk mengajak bercerita remaja. Pemberian informasi dari Nakes kepada remaja dan orangtua pun sudah baik, lengkap, dan sesuai dengan konteks permasalahan remaja.<br><br>Ruang untuk perbaikan: Ada baiknya jika untuk sesi konsultasi dengan remaja, Nakes meminta sesi tersendiri dengan remaja, dan orangtrua bisa menunggu di luar sejenak. Namun apabila tidak memungkinkan bagi orangtua untuk menunggu di luar, Nakes bisa mengatakan kepada orangtua bahwa. "Maaf Ibu, ini anaknya bisa menjawab sendiri, tolong izinkan saya mendengar langsung dari anaknya dulu." Selain itu, dalam membuat fokus atau pemetaan masalah dan membuat rencana perubahan, misal meningkatkan aktivitas fisik atau berolahraga, remaja bisa lebih banyak dilibatkan. Sebagai contoh, Nakes bisa mencoba bertanya kepada remaja, dari aktivitasnya sehari-hari menurut remaja tersebut apa yang kurang sehingga dia jadi obesitas, kemudian baru diprobing lagi terkait olahraga, misal olahraga yang disenangi, atau apa saja hambatan yang ditemui remaja dalam berolahraga selama ini. Sehingga fokus dan rencana perubahan dari sesi konsultasi bisa diarahkan ke meningkatkan aktivitas atau olahraga dan mengatasi hambatan-hambatannya. Hal lain yang juga perlu menjadi perhatian sebelum membahas fokus masalah dan rencana perubahan yang akan dilakukan adalah memperkaya informasi mengenai situasi remaja saat melakukan asesmen. Nakes memang telah melakukan asesmen di awal kepada remaja, akan tetapi asesmen yang dilakukan baru sebatas pola makan dan aktivitas saja, sementara ada kemungkinan, remaja sebenarnya sudah mengetahui bahwa dirinya memang kurang aktivitas fisik atau olahraga yang bisa berdampak pada peningkatan berat badan dan/ atau obesitas, akan tetapi hambatan bagi remaja untuk bisa berolahraga secara rutin bisa saja datang dari

		situasi rumah, sekolah, ataupun stress yang sedang dialami remaja. Hal-hal seperti situasi rumah, sekolah, dan stress yang dialami remaja perlu menjadi perhatian agar rencana perubahan yang dibuat semakin tepat sasaran.<br><br>Komentar secara keseluruhan: Secara umum, konsultasi remaja yang diberikan oleh Nakes masih bisa ditingkatkan lagi, terutama dalam hal asesmen dan keterlibatan remaja itu sendiri untuk menyadari dan menyusun rencana perubahan baik pola makan atau aktivitas untuk mengatasi masalah obesitasnya.
3	B3R1	Kekuatan konseling: Nakes memiliki kemampuan yang baik dalam membangun rapport dengan orang tua remaja, sehingga orang tua remaja nyaman untuk berbagi cerita. Selain itu, nakes juga sudah baik dalam meminta kesediaan partisipasi orang tua remaja, dengan memastikan pemahaman orang tua dan menjelaskan kembali poin-poin yang belum dipahami sepenuhnya oleh orang tua.<br><br>Ruang perbaikan: Konseling tidak melibatkan remaja, sehingga informasi hanya didapatkan dari orang tua. Akan lebih baik jika nakes meminta ada sesi remaja berbicara sendiri agar terdapat informasi dari remaja.<br><br><br>Komentar: Sesi konseling secara umum belum berjalan secara maksimal karena belum melibatkan partisipasi remaja di dalam sesi. Meskipun begitu, nakes sudah baik dalam berdiskusi peran-peran orang tua, khususnya mengenai peran dukungan orang tua di rumah (seperti dalam menyiapkan menu makanan maupun dalam mengajak remaja beraktivitas).
4	B4R1	Kekuatan: Proses konseling bisa membuat remaja bisa bercerita lebih dalam terkait keluhan dan halangannya, bisa memberikan insight mengenai fokus dan solusi yang muncul dari klien itu sendiri. Cara komunikasi yang digunakan oleh Nakes juga sudah baik dan bisa engage dalam diskusi atau konseling yang dilakukan. Penggunaan teknik Tanya-Beri-Tanya (TBT) dalam menyamnpaian informasi pun sudah dilakukan dengan baik dan Nakes tidak langsung memberikan informasi yang banyak kepada remaja, tetapi bertanya terlebih dahulu kebutuhan dan melakukan konfirmasi lagi. Hal ini pun membuat remaja merasa ikut terlibat aktif dalam menyelesaikan permasalahannya, sehingga muncul insight/ solusi yang datang dari remaja itu sendiri.<br><br>Ruang untuk perbaikan: Ada baiknya setelah pembahasan yang panjang ataupun di akhir sesi konseling, Nakes bisa memberikan rangkuman dari konseling/ diskusi yang telah dilakukan sepanjang sesi. Hal ini akan membantu remaja untuk meng-highlights poin-poin penting yang diperoleh remaja selama sesi sehingga remaja lebih mudah mengingat hal apa yang didapat dalam konseling dan hal apa yang perlu dilakukannya setelah konseling untuk memulai perubahan perilaku. Selain itu dalam konseling remaja, ada baiknya apabila Nakes melibatkan orangtua, karena orangtua bisa menjadi sumber dukungan dan memonitoring remaja agar melakukan perubahan perilaku di rumah, setelah konseling selesai. Untuk screening yang dilakukan sudah baik, karena Nakes sudah berusaha menanyakan situasi remaja di sekolah, termasuk rasa minder di

		sekolah terkait dengan kelebihan berat badannya, akan tetapi kurang diprobing kembali untuk mengarahkan ke prubahan perilaku. Pada saat screening, Nakes pun bisa melakukan screening mengenai kondisi personal remaja di rumah seperti apa, tidak hanya seputar makanan yang dimasak/ disajikan di rumah atau aktivitas saja. Kondisi remaja di rumah, sekolah, dan stress yang dialami bisa saja mempengaruhi pola makan (misalnya ada istilah emotional eating atau makan untuk menenagkan/ meredakan emosi). Hal lain yang juga penting untuk diperhatikan dalam konseling adalah pernyataan mengenai kerahassikan ada baiknya disampaikan di awal sesi, sehingga remaja akan merasa lebih aman dan nyaman bercerita, dan bisa jadi membuatnya lebih terbuka tentang kondisinya.<br><br>Komentar secara keseluruhan: Sesi konseling yang diberikan Nakes sudah baik dan masih bisa ditingkatkan lagi dalam beberapa hal, yaitu dalam melakukan screening bisa juga menambahkan screening mengenai kondisi di rumah, sekolah, dan stress, melibatkan orang tua untuk mendukung dan memonitoring perubahan perilaku yang akan dilakukan klien, serta memberikan keismpulan di akhir sesi konseling untuk meng- highlights poin-poin penting. Nakes terdengar percaya diri dan playful sehingga bisa membuat remaja merasa nyaman untuk sharing selama sesi konseling berlangsung.
5	B5R1	Kekuatan: Nakes menunjukan sikap ramah dan bisa membuat remaja nyaman. Selain itu sudah banyak menggunakan pertanyaan terbuka, probing yang diberikan sudah baik dan membuat remaja bisa lebih terbuka. Ada evoking yang membuat remaja merasa penting untuk memperhatikan kelebihan berat badan dan solusi yang dilakukan oleh remaja adalah senam. Selain itu juga ada afirmasi yang diberikan untuk menekankah bahwa yang dilakukan remaja seperti senam sudah benar atau baik.<br><br>Ruang untuk perbaikan: Ada planning yang muncul dari remaja namun hanya pada 1 solusi, yaitu senam. Mungkin bisa dieksplorasi lagi cara-cara lain yang menurut remaja juga bisa membantu dalam menurunkan berat badan selain senam. Selain itu, dalam pemberian informasi atau edukasi terkait kelebihan berat badan atau obesitas Nakes bisa lebih menggunakan teknik Tanya-Beri-Tanya (TBT) sehingga remaja lebih terlibat aktif secara 2 arah dalam pemberian informasi dalam konseling dan pemberian informasi tidak terkesan hanya 1 arah saja. Pada beberapa bagian konseling, Nakes terdengar sedang bingung akan menanyakan apa lagi kepada remaja, untuk meminimalisir kebingungan ini Nakes bisa lebih banyak melakukan refleksi terhadap pernyataan remaja dan mengarahkan ke prubahan perilaku, misalnya saat remaja menjawab bahwa dirinya jarang olahraga, maka Nakes bisa merefleksikan dengan menyatakan, “Oh, jarang ya untuk olahraga…” Kemudian, bisa diikuti dengan probing, “Apa yang membuat jarang berolahraga?” atau “Oh jarang ya, jarangnya berapa kali jika dalam seminggu?” setelah itu Nakes juga bisa menyisipkan informasi dengan teknik Tanya-Beri-Tanya (TBT), dengan berkata, “Pernah dengar ndak jika olahraga yang baik ini minimal berapa lama?” lalu nanti bisa mengikuti alur teknik TBT. Terakhir, sebaiknya pada akhir sesi, Nakes bisa menutup sesi dengan memberikan rangkuman mengenai poin-poin penting yang dibahas selama sesi, kemudian kesimpulan tersebut ada baiknya dikonfirmasi kembali ke remaja sebelum mengakhiri sesi.

		Komentar secara keseluruhan: Konseling yang dilakukan Nakes sudah cukup baik dan masih bisa ditingkatkan lagi. Memang pada beberapa bagian, Nakes sempat kebingungan. Terkait hal ini, mungkin Nakes bisa lebih yakin dan percaya diri lagi karena sebenarnya teknik konseling yang digunakan sudah cukup baik.
6	B6R1	Kekuatan: Proses konseling sudah fokus pada perubahan perilaku dan remaja juga terlibat aktif dalam menyusun rencana perubahan.<br><br>Ruang untuk perbaikan: Oleh karena sesi konseling ini untuk anak/ remaja, mungkin Nakes bisa lebih playful dalam memberikan pertanyaan dan merespon/ merefleksikan jawaban yang diberikan anak/remaja dalam sesi. Dengan menjadi lebih playful, seperti nada dan tempo suara dibuat lebih relaks akan membuat anak/ remaja merasa lebih nyaman selama sesi dan terdorong untuk lebih banyak bercerita tentang dirinya. Selain itu, untuk screening aspek psikososial perlu digali lagi terkait kondisi di rumah, sekolah, serta stress yang mungkin dialami remaja sehingga berdampak pada pola makan yang tidak seimbang, karena ada kemungkinan remaja cenderung banyak makan sebagai respon atau cara coping terhadap stress yang dialami atau disebut dengan perilaku emotional eating. Dengan mengetahui kondisi di rumah dan di sekolah, Nakes pun bisa mengetahui hambatan-hambatan yang dialami anak/ remaja dalam menjaga pola makan sesuai gizi seimbang, serta hambatan dalam berolahraga secara teratur, hal ini juga akan membuka peluang untuk bisa melibatkan orangtua dalam mendukung perubahan perilaku makan dan aktivitas pada anaknya. Kemudian, bisa lebih menerapkan teknik Tanya-Beri-Tanya (TBT) dalam memberikan informasi/ edukasi kepada anak/ remaja, misal Nakes bisa bertanya kepada anak tahukan makanan gizi seimbang itu seperti apa, lalu anak/remaja diberikan kesempatan menjawab, setelah itu Nakes bisa memberikan informasi tambahan atau mengelaborasikannya kembali sehingga anak / remaja akan memperoleh informasi yang lebih utuh dan valid, setelah itu Nakes bisa bertanya kembali tentang apakah remaja sudah merasa memenuhi makanan gizi seimbang itu dan jika belum, apa yang membuat belum bisa seperti itu. Terakhir, penting untuk membahas kemauan atau kemampuan anak/ remaja untuk berubah dan mengikuti rencana yang telah disusun.<br><br>Komentar secara keseluruhan: Sesi konseling yang diberikan Nakes masih bisa ditingkatkan lagi, Kekuatan konseling terletak pada fokus terhadap perubahan yang ingin dilakukan serta sudah melibatkan remaja secara aktif dalam menyusun rencana perubahan perilaku. Meski demikian, ada beberapa hal yang masih bisa ditingkatkan lagi dalam sesi konseling kedepannya, yaitu terkait dengan cara berkomunikasi dengan anak/ remaja bisa dibuat lebih playful, screening mengenai kondisi di rumah, sekolah, dan stress, penerapan teknik Tanya-Beri-Tanya (TBT), serta membahas tentang kemauan atau kemampuan remaja untuk mengikuti rencana yang sudah disusun. Nakes sudah berusaha membantu anak/ remaja selama dalam sesi untuk melakukan perubahan perilaku demi mengatasi obesitasnya.
7	B7R1	Kekuatan konseling: Berdasarkan hasil sesi konseling, nakes memiliki pengetahuan yang cukup baik terkait ilmu gizi dan kesehatan remaja. Pengetahuan tersebut membuat informasi yang diberikan kepada remaja

		menjadi bermanfaat untuk mendorong perubahan gaya hidup. Selain itu, nakes juga memiliki rapport yang baik dengan remaja sehingga remaja cukup terbuka untuk bercerita.<br><br>Ruang perbaikan: Sesi konseling terkesan kaku, seperti terpaku oleh daftar pertanyaan yang harus ditanyakan. Sebaiknya nakes lebih luwes dalam bertanya maupun merespon remaja, sehingga pertanyaan yang diberikan lebih sesuai dengan topik/konteks yang sedang dibahas.<br><br>Komentar: Secara umum, nakes belum melakukan teknik konseling MI maupun keterampilan komunikasi lainnya (seperti memberikan afirmasi, mendengar reflektif). Hal ini membuat proses konseling kurang melibatkan remaja, meskipun sesungguhnya remaja sudah bersemangat untuk mengikuti konseling. Namun, nakes sudah berusaha untuk mulai menyusun program untuk remaja, sehingga untuk sesi selanjutnya dapat mulai menggunakan teknik MI dalam mengevaluasi progress program yang telah disusun.
8	B8R1	-
9	B9R1	Kekuatan: Nakes sudah bisa mengaplikasikan skrining psikososial, memberikan afirmasi, melaksanakan konseling secara engaging, serta melibatkan orangtua dengan baik dalam hal dimensi tuntutan dan dukungan kepada remaja.<br><br>Ruang perbaikan: Ada baiknya saat konseling Nakes meminta agar remaja bisa berbicara sendiri, tanpa diinterupsi oleh orangtuanya. Penting juga menyatakan secara lisan mengenai kerahasiaan medis kepada remaja agar remaja bisa lebih nyaman dan terbuka lagi akan isu yang dihadapinya saat konseling berlangsung. Nakes juga sudah ada upaya melakukan evoking, namun perlu untuk lebih konsisten lagi dan mengarah pada perubahan perilaku. Setiap kali Nakes menyampaikan informasi dan membuat rangkuman, ada baiknya dikonfirmasi lagi kepada remaja.<br><br>Komentar secara keseluruhan: Nakes sudah bisa memberikan konseling remaja dengan baik dan masih bisa ditingkatkan lagi. Nakes menunjukkan rasa percaya diri dan menggunakan nada yang playful ketika berbicara dengan remaja, hal ini bisa menambah rasa nyaman dan membuat sesi konseling remaja menjadi semakin engaging.
10	B10R1	Kekuatan konseling: Nakes cukup baik dalam membangun rapport dengan orang tua, dan membuat orang tua lebih terbuka untuk menyampaikan informasi. Dari hasil sesi konseling, nakes juga memiliki pengetahuan yang baik dalam hal kesehatan remaja sehingga informasi yang diberikan kepada remaja dapat memotivasi remaja untuk mulai mengubah pola hidup (seperti mengurangi bermain ponsel).<br><br>Ruang perbaikan: Dari hasil sesi konseling, nakes masih belum menggunakan teknik MI sehingga remaja belum mengungkapkan motivasi dan rencana perubahan yang ingin dilakukan setelah menjalankan sesi. Selain itu, nakes juga kurang melibatkan remaja dan lebih berfokus pada komunikasi dengan orang

		tua, sehingga akan lebih baik jika remaja lebih dilibatkan atau diajak untuk berbicara sendiri pada sesi yang terpisah dari orang tua.<br><br>Komentar: Secara umum, sesi konseling masih terkesan service provider oriented, yaitu belum mengarahkan diskusi pada kekuatan dan keinginan dari remaja untuk mulai mengubah gaya hidupnya. Hal ini berdampak pada interaksi yang terkesan satu arah dan kurang terbuka akan pendapat dan pemikiran remaja atas rencana perubahan pola hidup yang direncanakan oleh nakes. Akan lebih baik jika nakes mulai menggunakan teknik MI agar sesi konseling berjalan lebih terbuka dan bersifat dua-arah.
11	B11R1	Kekuatan konseling: Nakes cukup baik dalam memilih pertanyaan untuk menggali jawaban dari remaja, sehingga remaja cukup nyaman untuk berbagi cerita. Nakes juga mampu mengidentifikasi kekuatan remaja dan memberikan afirmasi atas kebiasaan baik yang sudah dilakukan remaja.<br><br>Ruang perbaikan: Upaya evoking dan planning belum terlihat selama sesi, padahal ada beberapa waktu yang berpotensi dilakukan upaya evoking oleh nakes (seperti dengan bertanya bagaimana rasanya terhadap badan setelah aktif berolahraga). Hal ini berdampak pada remaja yang belum memiliki rencana atau action plan yang jelas dan konkret setelah sesi konseling selesai.<br><br>Komentar: Nakes secara umum sudah melakukan beberapa teknik dasar MI, sehingga konseling berjalan dengan lebih berfokus pada remaja dibandingkan berorientasi pada nakes. Namun, nakes belum melakukan teknik evoking dan planning yang sesungguhnya krusial dalam konseling MI.
12	B12R1	Kekuatan: Nakes sudah mengeksplorasi pola makan dan aktivitas remaja dengan cukup detail, pembicaraan yang dilakukan juga engaging dan tentunya bisa membuat remaja dan orangtua merasa nyaman.<br><br>Ruang untuk perbaikan: Aspek psikososial remaja belum banyak dieksplorasi. Walaupun ada sedikit bahasan tentang situasi di sekolah/ sekolah online, namun masih kurang didalami. Nakes bisa lebih mendalami aspek mengenai situasi di dalam keluarga, sekolah, dan stress yang mungkin berkaitan dengan obesitas atau pola makan serta aktivitas remaja, misalnya saja emotional eating sebagai respon stress yang bisa meningkatkan risiko obesitas. Selain itu, ada baiknya apabila Nakes meminta waktu agar remaja bisa berbicara lebih banyak dalam sesi, sehingga sesi konseling antara remaja dan orangtua tidak tercampur dan remaja bisa lebih banyak bercerita tentang kondisinya tanpa terlalu banyak diinterupsi orangtua.<br><br>Komentar secara keseluruhan: Konseling yang dilakukan sudah cukup baik dan masih bisa ditingkatkan lagi. Nakes sudah bisa mengeksplorasi dengan detail terkait pola makan dan aktivitas remaja, sudah bisa melibatkan orangtua, serta bisa membuat sesi nyaman baik bagi remaja dan orangtua yang terlibat.
13	B13R1	Kekuatan: Nakes terdengar ramah, sudah ada upaya focusing terhadap masalah yang dihadapi remaja, pertanyaan terbuka sudah banyak diberikan, ada bentuk afirmasi dan evoking yang playful dan sebenarnya bisa menarik anak untuk bisa

		bercerita lebikh lanjut. Sudah melibatkan orangtua dalam berdiskusi mengenai hal-hal yang perlu dipantai dari gizi ataupun pola makan remaja.<br><br>Ruang untuk perbaikan: Pertanyaan terbuka yang diberikan sudah baik, mungkin bisa ditingkatkan lagi probing, misalnya saja ketika bertanya kepada remaja apakah ia suka berolahraga, dan remaja menjawab tidak, Nakes bisa melakukan probing dengan menanyakan apa yang membuat remaja tidak suka berolahraga. Untuk planning/ perencanaan perubahan perilaku bisa lebih banyak melibatkan remaja, sehingga rencana perubahan perilaku tidak banyak datang dari Nakes tapi lebih banyak dari remajanya sendiri.<br><br>Komentar secara keseluruhan: Konseling yang dilakukan Nakes sudah cukup baik dan masih bisa ditingkatkan lagi. Nakes bisa melibatkan anak lebih banyak dengan meminta sesi berbicara dengan remaja atau meminta kepada orangtua bahwa pertanyaan yang diarahkan ke remaja, izinkanlah remaja menjawabnya sendiri. Selebihnya sudah baik dan terdengar nyaman.
14	B14R1	Kekuatan: Nakes sudah berusaha untuk mempraktekkan konseling dengan remaja. Nakes terdengar ramah dan sudah banyak pertanyaan terbuka yang diberikan. Ada upaya untuk menanyakan dulu mengenai informasi yang dibutuhkan, sebelum memberikan informasi tambahan.<br><br>Ruang untuk perbaikan: Ada baiknya apabila Nakes meminta sesi remaja untuk berbicara sendiri tanpa diinterupsi oleh orangtua, atau saat sesi Nakes boleh untuk meminta/ menjelaskan kepada orangtua bahwa remaja bisa menjawab sendiri, dan nanti akan ada sesi dimana Nakes akan berdiskusi dengan orangtua. Dengan demikian remaja akan bisa lebih terlibat aktif dalam konseling serta bisa terbuka lebih banyak akan isu-isu terkait yang mempengaruhi kelebihan berat badan atau obesitasnya. Pada saat screening, Nakes juga bisa memperkaya screening dengan menanyakan mengenai situasi remaja di rumah, sekolah, dan stress yang mungkin dialami, karena kondisi di rumah, sekolah, serta stress dapat berpengaruh terhadap pola maka (misal pada emotional eating, yaitu saat emosinya sedang tidak stabil dan tertekan, makan berlebih menjadi cara melampiaskan emosi). Untuk rencana perubahan perilaku pun Nakes bisa lebih banyak melibatkan remaja di dalam diskusi. Pada akhir sesi, Nakes bisa menutup sesi dengan memberikan rangkuman mengenai poin-poin penting yang dibahas selama sesi konseling dan konfirmasi kembali ke remaja, apakah ada hal yang ingin ditambahkannya. Hal ini untuk melihat apakah informasi yang Nakes tangkap dari remaja selama konseling berlangsung sudah tepat, dan apabila belum tepat, remaja sendiri yang akan mengoreksi maupun membetulkannya.<br><br>Komentar secara keseluruhan: Konseling yang dilakukan Nakes bisa ditingkatkan lagi. Misalnya saja Nakes bisa meminta waktu berbicara hanya dengan remaja, lebih melibatkan remaja dalam membuat rencana perubahan perilaku, dan memberikan kesimpulan/ rangkuman di akhir sesi.
15	B15R1	Kekuatan: Nakes terdengar ramah dan sebenarnya bisa sekali untuk engage dengan remaja, apalagi Nakes menggunakan nada yang lembut, temponya nyaman didengarkan, dan playful sehingga remaja pun bisa tertawa dalam sesi.

		Ruang untuk perbaikan: Berdasarkan konsultasi/ konseling yang diberikan oleh Nakes kepada remaja, belum terungkap dan terpetakan fokus permasalahan yang dialami oleh remaja, sehingga mengalami gizi berlebih. Hal ini bisa saja disebabkan oleh asesmen yang dilakukan kurang mendalam, karena asesmen lebih banyak fokus kepada pola makan dan aktivitas remaja saja, yang mana dalam kedua aspek itu tidak ditemukan permasalahan terkait berat badan remaja. Sehingga ada baiknya Nakes juga membahas mengenai situasi di rumah, sekolah (edukasi), serta stress yang mungkin dialami remaja, yang bisa berdampak kepada permasalahan kelebihan berat badan. Sebenarnya Nakes sudah cukup baik dengan menanyakan kepada remaja bahwa masalah peningkatan berat badannya dimulai di awal pandemi, namun sayang sekali hal ini tidak di probing lebih lanjut, sehingga fokus permasalahan dari sesi konsultasi/ konseling tidak banyak dibahas. Oleh karena fokus permasalahan tidak terbahas, hal ini berdampak pada kurangnya planning/ perencanaan yang bisa dilakukan remaja untuk melakukan perubahan dalam mengatasi masalah peningkatan berat badannya. Selain itu, dalam sesi konsultasi/ konseling dengan remaja, Nakes pun bisa lebih banyak menggunakan teknik Tanya-Beri-Tanya (TBT) untuk memberikan informasi/ edukasi kepada remaja, misalnya Nakes bisa menanyakan dahulu apakah remaja pernah mendengar atau tahu tentang gizi seimbang, nanti Nakes bisa meminta remaja bercerita mengenai apa yang remaja telah ketahui, kemudian Nakes bisa menambahkan informasi agar lebih lengkap. Hal ini akan membuat remaja merasa dihargai atas apa yang telah diketahuinya dan juga remaja bisa mendapatkan tambahan pengetahuan baru ketika mengikuti sesi konsultasi/ konseling dengan Nakes. Dengan demikian remaja akan lebih terbuka dan banyak bercerita kepada Nakes.<br>Komentar secara keseluruhan: Sesi konsultasi/ konseling yang dilakukan oleh Nakes masih dapat ditingkatkan lagi, bisa dengan cara melakukan asesmen yang lebih mendalam tidak hanya tentang pola makan dan aktivitas tetapi juga situasi di rumah, sekolah, dan stress yang mungkin berdampak kepada peningkatan berat badan dari remaja. Selain itu, memberikan kesempatan kepada remaja untuk bercerita lebih banyak tentang diri dan situasinya, terutama yang berkaitan dengan masalah peningkatan berat badannya, akan membuat Nakes lebih bisa melihat inti permasalahan dan bisa membantu remaja dengan lebih efektif.
16	B16R1	Kekuatan: Proses konseling bisa membuat remaja bisa bercerita lebih dalam terkait dirinya, screening psikososial yang dilaukan pun cukup baik karena bertanya tentang situasi di rumah dan sekolah serta tak hanya asesmen terhadap aktivitas ataupun pola makannya.<br><br>Ruang untuk perbaikan: Untuk perbaikannya, mungkin setelah memberikan refleksi, kesimpulan, maupun memberikan informasi, bisa dikonfirmasi atau ditanyakan lagi kepada remaja, bagaimana pendapatnya tentang informasi yang disampaikan oleh Nakes. Kemudian untuk rencana berubahan perilaku yang bisa dilanjutkan ataupun dilakukan oleh remaja untuk mengatasi kelebihan berat badannya, bisa dieksplorasi lagi hingga rencana perubahan tersebut lebih banyak muncul dari klien sendiri.

		Komentar secara keseluruhan: Konseling yang dialkukan Nakes sudah cukup baik dan bisa ditingkatkan lagi terutama terkait dengan pemberian refleksi, kesimpulan, dan informasi yang perlu dikonfirmasi lagi kepada remaja, serta dalam menyusun rencana perubahan perilaku masih bisa dieksplorasi lagi dan didiskusikan sehingga rencana perubahan perilaku lebih banyak muncul dari remaja itu sendiri.
17	B17R1	Kekuatan konseling: Nakes sudah baik dalam melakukan skrining secara menyeluruh untuk menggali area masalah yang dihadapi remaja. Nakes juga sudah mencoba melakukan evoking, untuk melihat keinginan remaja dalam mengubah berat badannya. Dari sesi konseling, nakes juga sudah melibatkan peran orang tua dan mengonfirmasi informasi dari remaja kepada orang tua.<br><br>Ruang perbaikan: Selama sesi konseling, akan lebih baik jika nakes lebih fokus untuk membangun dan mempertahankan motivasi remaja untuk menurunkan berat badan. Hal ini didasari karena remaja sesungguhnya sudah ada keinginan dan usaha untuk mengubah gaya hidup, hanya saja ia belum konsisten dalam mempertahankan usaha tersebut. Hal ini membuat seharusnya nuansa konseling lebih difokuskan untuk membahas bagaimana cara mempertahankan kebiasaan, atau bagaimana cara menghadapi kendala saat ingin berubah.<br><br>Komentar: Sesi konseling secara umum sudah berjalan dengan baik. Nakes mampu memetakan masalah-masalah yang dihadapi remaja, sehingga diharapkan remaja lebih termotivasi untuk mengubah gaya hidup. Akan lebih baik jika proses intro (awal konseling) dibuat lebih terstruktur dan lengkap (seperti bertanya kabar) agar kualitas konseling lebih baik lagi.
18	B18R1	Kekuatan konseling: Nakes mencoba untuk building rapport terlebih dahulu di awal konseling, agar remaja merasa lebih nyaman untuk bercerita. Selama sesi konseling, remaja juga cenderung irit untuk berbicara, namun nakes memiliki skill probing dan bertanya pertanyaan terbuka yang cukup baik agar remaja lebih tertarik untuk bercerita lebih banyak.<br><br>Ruang perbaikan: Dari proses komunikasi, sebaiknya building rapport diberikan alokasi waktu yang lebih lama agar remaja dapat lebih nyaman untuk bercerita. Nakes juga belum menggunakan teknik MI selama sesi konseling, sehingga pemahaman nakes terhadap makna 'konseling' masih berupa memberikan saran satu arah tanpa memeriksa pemahaman remaja terlebih dahulu.<br><br>Komentar: Sesi konseling secara umum terkesan berjalan searah, dan nakes terkesan kesulitan untuk menggali informasi dari remaja meskipun sudah mencoba building rapport singkat di awal. Jika nakes lebih aktif menggunakan teknik MI maupun teknik konseling seperti bertanya terbuka atau memberikan afirmasi, hal tersebut berpotensi semakin memicu remaja untuk lebih banyak bercerita.
19	B19R1	Kekuatan: Screening psikososial yang dilakukan dalam konseling sudah cukup lengkap, karena juga membahas memgenai situasi di rumah, sekolah, dan stress yang dialami anak/ remaja, tidak hanya tentang pola makan dan aktivitasnya.

		Ruang untuk perbaikan: Meskipun screening sudah cukup lengkap, akan tetapi Nakes masih terlalu banyak membahas hal-hal yang kurang berkaitan dengan isu berat badan berlebih atau obesitas, sehingga fokus dalam sesi pun tidak begitu jelas. Selain itu di dalam sesi, planning/ rencana perubahan perilaku juga belum begitu terbahas akibat belum ditentukannya fokus dari sesi konseling.<br><br>Komentar secara keseluruhan: Konseling yang dilakukan Nakes bisa ditingkatkan lagi dalam hal fokus permasalahan dan perencanaan perubahan perilaku yang bisa dilakukan anak/ remaja. Selebihnya, Nakes sudah bisa membuat anak/ remaja bercerita banyak tentang dirinya.
20	B20R1	Kekuatan konseling: Nakes mampu mengidentifikasi usaha-usaha yang sudah dilakukan oleh remaja, dan sudah mencoba melakukan evoking untuk membantu remaja menjadi lebih konsisten dalam mengelola pola makan dan gaya hidup.<br><br>Ruang perbaikan: Selama sesi konseling, nakes belum memaksimalkan peran orang tua dalam mendukung maupun memberikan tuntutan terhadap perubahan pola hidup remaja untuk mengelola obesitas. Orang tua khususnya ibu dapat dilibatkan dengan berdiskusi lebih lanjut mengenai penyusunan menu makanan, atau mendukung remaja agar lebih aktif dalam berolahraga.<br><br>Komentar: Dari hasil sesi, secara umum nakes sudah mulai menggunakan teknik-teknik dasar dalam MI. Nakes dapat memaksimalkan kualitas sesi dengan berdikusi lebih lanjut dengan remaja tanpa orang tua, serta memberikan informasi dengan teknik TBT yang lebih bervariasi sesuai dengan konteks pembicaraan yang sedang berlangsung.
21	B21R1	Kekuatan konseling: Konseling dilakukan dengan singkat, padat, dan jelas oleh nakes. Selama sesi, nakes juga cukup fokus dalam menggali permasalahan gaya hidup terkait dengan pola makan dan kebiasaan berolahraga.<br><br>Ruang perbaikan: Sesi konseling yang berjalan singkat membuat area masalah yang dihadapi oleh remaja belum sepenuhnya tergali. Dari rekaman yang diberikan, kesan yang muncul dari sesi konseling ini cenderung terburu-buru dan seperti scripted (sudah direncanakan sebelumnya). Hal ini membuat sesi konseling menjadi kurang adekuat untuk membantu remaja mengelola obesitasnya. Sehingga, sebaiknya nakes lebih tenang dan menyiapkan waktu lebih agar sesi konseling tidak berjalan terlalu singkat.<br><br>Komentar: Secara umum, belum terlihat adanya penggunaan teknik MI maupun teknik konseling dasar yang digunakan selama sesi dilakukan. Kondisi ini membuat sesi terkesan juga terkesan tidak dilaksanakan secara maksimal.
22	B22R1	Kekuatan konseling: Meskipun baru sesi pertama, konseling sudah berjalan dengan baik dan nakes melakukan beberapa teknik MI dengan baik seperti engaging, focusing, dan evoking. Nakes juga secara teliti melakukan skrining HEADS pada seluruh aspek, sehingga dapat menggambarkan area masalah remaja secara lebih menyeluruh.

		Ruang perbaikan: Sebaiknya nakes lebih percaya diri dalam bertanya pertanyaan yang sensitif, seperti saat bertanya perihal kehidupan seksual saat skrining HEADS. Karena saat tidak percaya diri, nakes jadi cenderung tertawa saat bertanya yang membuat komunikasi jadi canggung dengan remaja.<br><br>Komentar: Nakes sudah mampu melakukan building rapport dengan baik, dan mampu memberikan pertanyaan terbuka serta probing untuk mendorong remaja lebih banyak berbicara tentang situasinya. Hal ini membuat sesi konseling berjalan dengan cukup lancar dan dapat menghimpun banyak informasi penting dari remaja sejak awal sesi.
23	B23R1	Kekuatan: Nakes sudah mengaplikasikan pertanyaan terbuka, probing, dan melakukan eksplorasi/ asesmen terhadap aspek psikososial remaja (situasi di rumah, sekolah, dan stress) dengan baik serta cukup lengkap.<br><br>Ruang perbaikan: Dalam memberikan informasi, Nakes bisa menggunakan teknik Tanya-Beri-Tanya, sehingga tidak langsung memberikan informasi kepada remaja. Selain itu, Nakes juga bisa lebih banyak memberikan afirmasi dan refleksi untuk membuat remaja bisa lebih bercerita tentang dirinya. Jika memungkinkan, ada baiknya juga saat sesi konseling berlangsung, orangtua bisa diminta menunggu di ruangan lain dulu dan barulah orangtua ikut disaat Nakes ingin berdiskusi dengan orangtua. Hal ini bisa membantu remaja untuk lebih terbuka tentang diri dan dunianya, karena bisa saja remaja jadi kurang terbuka akibat orangtuanya ada di sampingnya dan mendengarkan seluruh sesi konseling.<br><br>Komentar secara keseluruhan: Pada dasarnya Nakes sudah cukup baik dalam melakukan konseling dan masih bisa ditingkatkan lagi. Nakes sudah terdengar nyaman, penuh percaya diri, dan lancar dalam memberikan konseling kepada remaja.
24	B24R1	Kekuatan konseling: Nakes sangat informatif dalam memberikan edukasi terhadap remaja, dan mampu mendorong remaja untuk ingin bertanya lebih lanjut mengenai masalah obesitas yang sedang dihadapi.<br><br>Ruang perbaikan: Teknik konseling yang digunakan masih bersifat tradisional atau seperti konseling kesehatan pada umumnya, sehingga nakes tidak memberi ruang bagi remaja untuk menyampaikan perubahan, maupun mengonfirmasi pengetahuan remaja terhadap masalah yang sedang dihadapi.<br><br>Komentar: Berdasarkan hasil sesi, secara umum sesi konseling belum terkesan seperti sesi konseling menggunakan teknik-teknik MI. Nakes sebaiknya lebih bersikap terbuka, dan memposisikan diri layaknya rekan suportif terhadap remaja dalam mencapai perubahan gaya hidup, dan bukan seperti ahli.
25	B25R1	Kekuatan konseling: Nakes melakukan skrining HEADS dengan baik dan lengkap, sehingga area masalah yang dialami remaja berkaitan denga pola makan dan aktivitas menjadi lebih tergambarkan. Nakes juga melaksanakan sesi konseling secara terstruktur, sehingga mudah untuk dipahami oleh remaja maupun orang tua.

		Ruang perbaikan: Nakes masih terlalu fokus pada memberikan saran untuk remaja, dan tidak mengonfirmasi pemahaman maupun pengetahuan remaja dan orang tua terhadap informasi dan rancangan program yang diberikan oleh nakes.<br><br>Komentar: Jika melihat secara keseluruhan, nakes sudah cukup baik dalam melakukan sesi konseling meskipun belum sepenuhnya mengaplikasikan teknik MI. Terdengar bahwa remaja yang sebelumnya tertutup menjadi lebih terbuka dan nyaman untuk berbicara, namun akan lebih baik jika nakes juga mengaplikasikan teknik MI seperti evoking dan planning agar rancangan perubahan perilaku diungkapkan oleh remaja.
26	B26R1	Kekuatan konseling: Nakes mampu memberikan pertanyaan terbuka dengan baik sehingga remaja terdorong untuk menjawab secara lebih elaboratif. Nakes juga mampu melihat kekuatan dan konsistensi dalam dir remaja sehingga secara aktif memberikan afirmasi terhadap paparan remaja mengenai usaha yang telah dilakukan.<br><br>Ruang perbaikan: Di awal sesi, nakes cenderung kaku dan terkesan terpaku oleh daftar pertanyaan, meskipun remaja sudah cukup ramah dan banyak berbicara sejak awal. Nakes juga kurang menggali lebih lanjut mengenai masalah atau kendala yang mungkin dialami di rumah, atau dengan orang tua/keluarga.<br><br>Komentar: Secara keseluruhan, nakes sudah mampu melakukan teknik MI dengan baik. Nakes memberikan pertanyaan yang cukup bervariasi dan sesuai dengan tujuan dari pelaksanaan sesi. Hanya saja, Nakes kurang aktif dalam menggunakan teknik reflektif sehingga di awal, sesi terkesan interogatif.
27	B27R1	Kekuatan konseling: Nakes mampu memetakan masalah remaja dengan baik melalui skrining terhadap situasi di rumah maupun kampus yang berkontribusi terhadap berat badan remaja.<br><br>Ruang perbaikan: Nakes belum melakukan teknik-teknik MI dengan tepat, karena masih berkomunikasi secara satu arah (service provider oriented). Sebaiknya nakes lebih banyak melakukan teknik-teknik MI untuk memicu remaja menemukan solusi dan membangun rencana atas masalahnya sendiri.<br><br>Komentar: Secara umum, nakes dalam berkomunikasi masih cenderung tertutup dan hanya berfokus pada memberikan saran, sehingga kurang melibatkan peran remaja maupun orang tua dalam membangun perubahan pola hidup untuk remaja tersebut.<br><br>Tambahan kekuatan konseling: Dari hasil konseling, nakes juga cukup proaktif dalam memberikan pertanyaan terbuka maupun pertanyaan tertutup secara seimbang, untuk menggali informasi dari remaja. Remaja sesungguhnya cukup canggung dalam berbicara, namun nakes mampu melakukan probing agar remaja lebih banyak bercerita.
28	B28R1	Kekuatan: Nakes terdengar ramah dan sebenarnya sangat bisa apabila engage serta dekat dengan anak dan remaja. Informasi yang disampaikan dengan jelas sehingga terdengar orangtua paham tentang gizi seimbang.

		Ruang untuk perbaikan: Nakes bisa meminta sesi tersendiri untuk berbicara dengan anak/ remaja. Hal ini bertujuan agar remaja bisa lebih bercerita tentang diri dan kondisinya. Asesmen yang dilakukan pun tidak hanya screening saja, tetapi juga pemetaan masalah yang dialami oleh anak/ remaja terkait kelebihan berat badan atau obesitas. Dalam hal ini Nakes bisa mendiskusikan/ bertanya mengenai situasi anak di rumah, sekolah, dan stress yang dialaminya, yang mungkin membuat pola makan anak/ remaja menjadi tidak teratur seperti melakukan emotional eating (makan untuk meredakan atau menenangkan emosi) serta hambatan-hambatan yang dialami anak/ remaja untuk beraktivitas fisik/ berolah raga. Selain itu, dalam penyampaian informasi, Nakes bisa lebih menggunakan teknik Tanya-Beri-Tanya (TBT), sehingga ada diskusi/ orbolan yang mengalir maupun interaksi dua arah antara Nakes dengan anak/ remaja. Nakes pun bisa memperbanyak memberikan pertanyaan yang sifatnya terbuka kepada anak/ remaja, sehingga akan memacu remaja untuk lebih banyak bercerita tentang dirinya, terkait masalah kelebihan berat badan atau obesitas.<br><br>Komentar secara keseluruhan: Konseling yang dilakukan Nakes bisa ditingkatkan lagi dengan meminta sesi konseling untuk berbicara dengan remaja, kemudian memperbanyak pertanyaan terbuka, melakukan asesmen mengenai kondisi/ situasi rumah, sekolah, dan stress yang dialami, serta menyampaikan informasi dengan teknik Tanya-Beri-Tanya (TBT). Dengan meningkatkan hal tersebut, ditambah dengan Nakes yang memang sudah ramah, dan mampu memberikan informasi dengan tepat serta mudah dipahami, maka sesi konseling akan menjadi lebih efektif dan juga memberikan lebih banyak manfaat pada anak/ remaja dan orangtuanya.
29	B29R1	Kekuatan konseling: Selama sesi konseling, nakes melibatkan orang tua dan bertanya lebih lanjut mengenai peran orang tua dalam memberikan tuntutan terhadap remaja agar dapat hidup lebih sehat.<br><br>Ruang perbaikan: Sesi secara umum berjalan seperti wawancara, tanpa ada usaha yang berarti untuk membangun motivasi perubahan pola hidup pada remaja, maupun untuk membangun engagement dan fokus masalah selama sesi berlangsung. Sebaiknya, nakes mulai menggunakan teknik MI agar remaja dapat mengungkapkan motivasinya untuk melakukan perubahan.<br><br>Komentar: Secara umum, sesi yang berjalan singkat membuat rapport antara nakes dengan remaja dan orang tua remaja belum terbangun dengan baik. Hal ini membuat diskusi berjalan tidak optimal, meskipun orang tua dan remaja sudah cukup antusias untuk berkomunikasi dengan nakes.<br><br>Tambahan kekuatan konseling: Selama sesi konseling, orang tua cenderung mendominasi jalannya sesi. Namun, dengan kondisi tersebut nakes tetap mencoba mengonfirmasi informasi yang diterima dari orang tua kepada remaja. Adanya konfirmasi ini membuat nakes tetap mementingkan pendapat remaja, dan memeriksa kebenaran informasi yang diberikan orang tua agar sesuai dengan kondisi remaja.

30	B30R1	Kekuatan: Nakes menyediakan ilustrasi/ gambar dalam menjelaskan gizi seimbang sehingga lebih mudah dipahami oleh anak/ remaja dan orangtuanya.<br><br>Ruang untuk perbaikan: Nakes lebih banyak memberikan informasi kepada anak/ remaja dan orangtua sehingga belum menggunakan teknik konseling Tanya-Beri-Tanya (TBT) secara efektif, hal ini membuat sesi seolah terfokus pada psikoedukasi atau pemberian informasi saja, tanpa banyak membahas mengenai konsisi anak/ remaja di rumah, sekolah, dan stress yang mempengaruhi permasalahan berat badan atau obesitasnya. Selain itu, pada sesi Nakes lebih banyak berdiskusi dengan orangtua/ ibu daripada langsung menanyakan kepada anak/ remaja yang bersangkutan.<br><br>Komentar secara keseluruhan: Konseling yang dilakukan Nakes bisa ditingkatkan lagi, terutama dengan meminta sesi berbicara dengan anak/ remaja dan menerapkan teknik Tanya-Beri-Tanya (TBT) dalam menyampaikan informasi. Di luar hal tersebut, dari pembawaan Nakes yang tenang dan tidak terburu-buru dalam menyampaikan sesuatu, sebenarnya memiliki potensi untuk bisa engage dengan anak/ remaja, hanya saja memang perlu ada sesi berbicara dengan anak/ remaja.
31	B31R1	Kekuatan: Nakes terdengar ramah, dan sepertinya cukup baik dalam membuka awalan sesi.<br><br>Ruang untuk perbaikan: Sepertinya rekaman yang diberikan Nakes terpotong, karena hanya 2 menit dan itupun hanya awalan sesi saja. Terkait awasan sesi, sebaiknya Nakes meminta sesi untuk berbicara dengan remaja tanpa ditemani orangrua, atau meminta agar ornagtua tidak ikut mengulang pertanyaan Nakes kepada remaja. Pada awal sesi juga penting bagi Nakes untuk mengungkapkan secara lisan mengenai pernyataan kerahasiaan medis agar remaja merasa lebih nyaman untuk terbuka pada sesi konseling.<br><br>Komentar secara keseluruhan: Konseling yang dilakukan Nakes bisa ditingkatkan lagi. Mungkin sebaiknya dipastikan rekaman audionya masih menyala sepanjang sesi, karena sepertinya rekaman yang di-summit oleh Nakes terpotong di awal sesi.
32	B32R1	Kekuatan: Nakes sudah memberikan skrining dengan baik terkait kondisi psikososial remaja, seperti kondisi di rumah, Pondok Pesantren (pendidikan), dan stress yang mungkin dialami remaja, yang mana hal ini bisa mempengaruhi kelebihan berat badannya. Selain itu Nakes juga sudah mengaplikasikan dengan terkait penggunaan pertanyaan terbuka, probing, memberikan rangkuman dan mengkonfirmasi kembali isi rangkuman ke remaja, evoking, perencanaan yang diupayakan datang dari remajanya sendiri, serta penggunaan teknik Tanya-Beri-Tanya dengan lengkap, sehingga Nakes tidak terkesan langsung memberikan informasi.<br><br>Ruang perbaikan: Nakes masih perlu secara lisan mengungkapkan mengenai kerahasiaan data kepada remaja, serta Nakes bisa meminta sesi dimana remaja bebricara sendiri dulu, untuk meminimalkan interupsi dari orangtua. Apabila memungkinkan orangtua bisa diminta menunggu di ruangan lain atau di luar,

		agar remaja bisa lebih terbuka, karena di usia remaja, biasanya sebagai individu sudah mulai memiliki rasa privasi, bahwa ada hal-hal yang bisa di share secara terbuka kepada orangtuanya dan ada juga hal-hal yang remaja jaga agar orangtuanya jangan sampai tahu dulu, selama hal itu tak berbahaya bagi remaja dan remaja juga tidak membahayakan orang lain.<br><br>Komentar secara keseluruhan: Nakes sudah melaksanakan sesi konseling remaja dengan baik dan masih ada peluang untuk ditingkatkan lagi. Nakes sudah bisa mengaplikasikan teknik-teknik MI, meskipun di satu titik, Nakes masih merasa ragu atau kehabisan pertanyaan, namun disini Nakes tetap berusaha melanjutkan sesinya hingga selesai dan ini adalah hal yang baik. Nakes pun berusaha membuat agar remaja lebih banyak bercerita dengan mengarahkan pertanyaan langsung ke remaja disaat orangtua menginterupsi atau menjawab pertanyaan untuk remaja. Hal ini berhasil membuat remaja terbuka dan bercerita tentang kondisinya, menurut perspektifnya sendiri.

A 2.

DAFTAR KOMENTAR KELOMPOK A - Unggah Audio Kasus 2 - Konseling ke-1

NO	NAMA DEPAN	KOMENTAR
1	[redacted]	Kekuatan<br>1. Nakes terdengar antusias dan hangat; dapat membangun rapport dgn baik, bisa menyesuaikan dgn cara berkomunikasi pasien remaja maupun ortu.<br>2. Nakes menjelaskan cara pengelolaan badan dengan mudah dipahami oleh pasien remaja.<br>3. Sudah ada pertanyaan terbuka yg dilanjutkan dgn probing untuk mendapatkan gambaran pola makan/ aktivitas pasien.<br>4. Ada upaya evoking dengan menanyakan apakah ada keinginan untuk menurunkan BB dan melakukan probing mengapa sulit menurunkan badan, namun belum diprobing dimana letak kesulitan pasien untuk menurunkan BB. Jika diprobing dan ditemukan letak kesulitannya, mungkin bisa menjadi sarana untuk memetakan rencana perubahan yg bs dilakukan terkait pengelolaan BB.<br><br>Ruang Perbaikan<br>1. Sebaiknya sesi dibuka dengan menanyakan kabar, menanyakan keluhan, atau menjelaskan maksud/tujuan/ kerahasiaan sesi dibandingkan langsung menanyakan kepada pasien, "apakah kamu ada yg ingin ditanyakan ttg BB?"<br>2. Pada saat konseling dgn pasien remaja, remaja bisa lebih dilibatkan untuk mengidentifikasi letak masalah yang menyebabkan BB berlebih dan pola makan/hidup yg bermasalah. Pasien remaja cukup talkative sehingga mungkin bisa ditanya pendapatnya, kira - kira menurut pasien perubahan apa yg bisa direncanakan untuk mengelola BB.<br><br>Komentar Umum<br>Nakes membangun rapport dgn cukup baik, namun bisa lebih ditingkatkan prinsip2 MI, seperti engaging - focusing - evoking - planning dalam percakapan.
2	[redacted]	Kekuatan: 1) Melakukan skrining kondisi dengan lengkap 2) Memberikan informsi dampak negatif yang mendukung perubahan<br>Aspek yang perlu diperbaiki: Belum banyak menggali motivasi pasien untuk berubah dan melibatkan untuk membuat rencana perubahan<br>Komentar keseluruhan: Menggali skrining dengan baik namun perubahan masih diarahkan dan belum banyak membuat pasien remaja menceritakan kondisi, kesulitan dan motivasi untuk berubah.
3	[redacted]	Kelebihan:<br>- Menggali skrining dengan detail untuk memahami kondisi remaja<br>- Melakukan reflective dengan baik<br><br>Kekurangan:<br>-Suara rekaman sulit didengar terlalu banyak noise<br>- Lebih banyak bertanya dengan orang tua dan cenderung menggali dengan format tertutup<br><br>Komentar umum:<br>Proses MI masih lebih banyak melibatkan orang tua dibanding remaja

4	[redacted]	Rekaman berdurasi kurang dari 1 menit dan berisi monolog pasien (?) yang menjelaskan identitas diri (nama, usia, kelas, TB/BB) serta pola makan, menu, dan aktivitas hariannya. Informasi dasar yang diberikan oleh pasien sudah cukup lengkap, namun tidak terdapat interaksi dua arah antara tenaga kesehatan dan pasien.
5	[redacted]	Kekuatan: 1) Menggali kondisi asupan dan aktivitas dengan lengkap 2) Melakukan evoking dengan baik dengan menjelaskan urgensi 3) Berusaha menanyakan remaja tentang rencana perubahan<br>Aspek yang bisa diperbaiki: 1) Lebih banyak memberikan pertanyaan terbuka untuk menanyakan kesulitan dan motivasi remaja untuk berubah<br>Komentar keseluruhan: Proses dilakukan dengan lengkap dan sistematis
6	[redacted]	Kekuatan<br>1. Meski pasien remaja berusia 10 tahun dan tidak terlalu aktif menjawab, namun nakes berusaha untuk engage dengan pasien remaja, misalnya dengan memanggil dengan sebutan "Kakak" dan menanyakan kepada pasien lebih dulu (tidak langsung kepada ibu) untuk data2 yg sekiranya pasien bisa jawab (co: usia, lahir tahun baru, kegiatan sehari - hari, dll).<br>2. Nakes sudah banyak menggunakan pertanyaan terbuka dan melakukan probing lanjutan. Nakes cukup sabar untuk melakukan probing; probing yang dilakukan cukup detil dan sesekali memasukkan ilustrasi sederhana agar dapat dipahami oleh pasien remaja.<br>3. Saat membuat perencanaan perubahan perilaku, nakes berusaha untuk melakukan evoking alasan perubahan ("Ingin nggak berubah menjadi lebih sehat?") dan menanyakan rencana apa yang dimiliki oleh orangtua untuk mengelola BB pasien. Ketika rencana keluar dari pihak orangtua/ pasien, rencana perubahan juga mengandung data mengenai kesulitan yang dialami untuk anak melakukan aktivitas fisik lebih.<br><br>Ruang Perbaikan:<br>1. Setelah membuat rangkuman atas data yang diberikan oleh pasien/ortu, sebaiknya dikonfirmasi apakah pemahaman nakes sejauh ini sudah tepat.<br>2. Nakes dapat memperjelas poin - poin dukungan dan tuntutan yg dapat dilakukan oleh orrang tua untuk pengelolaan BB pasien.<br><br>Komentar Umum<br>Nakes sangat sabar dalam melakukan konseling. Meski pasien remaja tidak selalu bisa menjawab pertanyaan dan ortu pasien cenderung menjawab untuk anaknya, namun nakes selalu berusaha untuk mengajukan pertanyaan kepada anak terlebih dahulu. Nakes juga berusaha menyederhanakan pertanyaan/ memberikan ilustrasi agar pertanyaan lebih mudah dipahami bagi pasien remaja. Nakes sudah banyak melakukan prinsip2 MI di dalam sesi konselingnya.
7	[redacted]	Kekuatan: 1) Melakukan skrining dengan lengkap 2) Memberikan informasi dampak negatif kondisi yang mendukung perubahan<br>Aspek yang perlu diperbaiki: 1) Proses untuk perubahan dan membuat rencana cenderung diarahkan langsung oleh peserta tanpa banyak melibatkan remaja 2) Belum dapat merespon kekhawatiran remaja jika gagal dengan refleksi<br>Komentar keseluruhan: Proses menggali informasi detail namun untuk perubahan masih terlalu diarahkan oleh peserta

8	[redacted]	-
9		Kekuatan:<br>- Mencoba melakukan evoking dengan mempertimbangkan hal yang penting bagi remaja<br>- Memberikan informasi yang dibutuhkan<br><br>Kekurangan:<br>- Cenderung satu arah, lebih banyak memberikan saran<br>- Banyak pertanyaan tertutup yang membuat remaja sulit bercerita<br>- Di awal banyak info yang cenderung tidak relevan dan tidak mempertimbangkan kebutuhan info tersebut di remaja<br><br>Komentar umum:<br>Proses cenderung satu arah lebih banyak memberikan saran dan nasihat
10	[redacted]	Kekuatan: 1) Skirining dilakukan detail 2) Melakukan focusing denngan baik yang dapat mendukung perubahan<br>Aspek yang bisa diperbaiki: Lebih banyak menggali kesulitan pasien dan mengukur motivasinya untuk berubah melalui scaling dan pertanyaan terbuka<br>Komentar: Dilakukan dengan detail dan prosesnya sistematis dan lancar
11	[redacted]	Kekuatan:<br>1. Menggali kondisi pasien remaja dengan detail mulai dari apa yang dikonsumsi, aktivitas fisik, dll 2. Prosesnya sistematis mulai dari menggali kondisi, apa yang mau diubah, dan bagaimana mengubahnya<br>Aspek yang dapat diperbaiki 1) Belum banyak memberikan afirmasi 2) Planning masih diarahkan dan belum dilakukan bersama remaja 3) Ketika bertanya terlihat seperti menggali kesalahan pasien yang dapat membuat tidak nyaman<br>Komentar keseluruhan: Proses dilakukan dengan detail dan sistematis namun untuk perubahan masih cenderung diarahkan dan belum banyak melibatkan remaja
12	[redacted]	Kekuatan<br>1. Melakukan screening HEAADSSS secara lengkap,<br>2. Menormalisasi dan menjelaskan dengan straightforward mengenai topik - topik dalam screening yg agak sensitif, seperti bullying, pelecehan seksual, aktivitas seksual, konsumsi obat - obatan,<br>3. Informasi yang diberikan cukup jelas dan sederhana menyesuaikan dgn kapasitas anak usia 11 tahun.<br>4. Pada sesi konseling kedua (follow up) sudah menggunakan pertanyaan terbuka yg dilanjutkan dgn probing. Nakes juga sudah melakukan 1x afirmasi (saat memuji kegiatan fisik pasien).<br><br>Ruang Perbaikan<br>1. Pemberian informasi dapat diberikan dengan teknik TBT (Tanya - Beri - Tanya) untuk mengetahui informasi apa yg sudah dimiliki oleh pasien dan nakes menambahkan atau mengklarifikasi bila ada yg salah.<br>2. Focusing masalah dan planning perubahan perilaku sebaiknya datang dari pasien (i.e. menanyakan kepada pasien apa yg menurutnya menjadi penyebab masalah, menanyakan apa yg kira - kira bisa dilakukan untuk mengelola BB, bagaimana kemampuan pasien untuk menjalankan rencana perubahan).

		3. Agar pasien lebih termotivasi menjalankan perubahan, maka ada baiknya digali apa yg menjadi alasan bagi pasien untuk berubah, konsekuensi positif apa yg ingin didapatkan jika berubah, dll (Tahap evoking).<br><br>Komentar Umum<br>Selama konseling terlihat upaya untuk memberikan penjelasan yg mudah dipahami bagi pasien usia 11 tahun, namun konseling belum telihat 2 arah karena lebih berfokus pada pemberian informasi kepada pasien dan kurang menggali alasan pasien untuk berubah, pendapat pasien ttg sumber masalah BB, dan rencana yg kira - kira dapat ia lakukan untuk mengelola BBnya. Pertanyaan yg diajukan sudah cukup terbuka dan ada sejumlah probing serta afirmasi atas hal yg sudah baik dilakukan oleh pasien. Konseling sesi 1 dan sesi 2 sepertinya dilakukan oleh nakes yg berbeda.
13	[redacted]	Kekuatan<br>1. Nakes membuka konseling dengan menanyakan apakah pasien sudah mulai melaksanakan pengaturan pola makan sejak sesi pertama.<br>2. Nakes berusaha menjelaskan menu makan dan pola aktivitas fisik secara detil, sederhana, dan menyesuaikan kondisi pasien.<br><br>Ruang Perbaikan<br>1. Informasi dapat diberikan menggunakan prinsip TBT (Tanya - Beri - Tanya), misalnya didahului dengan menanyakan "menurut pasien seperti apa porsi dan menu makan yang sehat?" sebelum memberikan informasi mengenai menu dan diakhiri dengan menanyakan kembali apa pendapat pasien ttg informasi menu makan yang diberikan, apakah bisa dilakukan.<br><br>Komentar Umum:<br>Sesi ini merupakan konseling sesi kedua, nakes sudah berusaha memberikan informasi secara jelas dan detil mengenai menu dan pola makan yang sehat. Namun demikian, sesi masih bersifat 1 arah belum terlalu banyak menerapkan prinsip Motivational Interview.
14	[redacted]	Kekuatan: 1) Menjelaskan tentang tujuan dan manfaat sesi 2) Menjelaskan rencana sesi<br>Aspek yang perlu diperbaiki: Belum melibatkan remaja dan melakukan proses MI<br>Komentar keseluruhan: Proses baru menjelaskan tentang manfaat dan tujuan sesi namun belum ada proses yang menggunakan teknik MI
15	[redacted]	Kekuatan<br>1. Nakes mengajukan pertanyaan yang membantu untuk memetakan faktor bermasalah yang menyebabkan kelebihan BB (i.e. banyak jajan manis2)<br>2. Nakes berusaha untuk memberikan penjelasan yg mudah dipahami mengenai alasan mengapa pasien perlu berubah (co: pasien usia 1 tahun 66 kg, bgmn kalau usia 20an tahun?; pasien dengan ortu beda BB hanya sekitar 3 - 4 kg; jika BB kelebihan paru2 tidak kuat untuk jalan/ mendaki).<br><br>Ruang Perbaikan:<br>1. Nakes dapat lebih banyak memberikan pertanyaan terbuka dan memberikan kesempatan kepada pasien untuk menjawab secara mandiri; mungkin dapat dipertimbangkan untuk mengadakan sesi terpisah dari ortu agar pasien bisa lebih

		banyak bicara,<br>2. Nakes bisa lebih banyak menggunakan prinsip TBT saat memberikan informasi, yaitu dengan menanyakan dulu kepada pasien misalnya apa yg menurut pasien menyebabkan BB nya berlebih, apa yang diketahui ttg pola makan yang baik, apa yg menurut pasien bisa dilakukan untuk mengelola BB nya. Pada sesi ini, lebih banyak nakes yang memberikan informasi ttg masalah pasien, apa yang pasien bisa lakukan, dll sehingga lebih terkesan 1 arah (kurang kolaboratif).<br><br>Komentar Umum<br>Sesi konseling dilakukan dengan menggunakan Bahasa Daerah sehingga pada beberapa bagian kurang tertangkap apakah nakes sudah menerapkan teknik MI dan konseling masih lebih banyak bersifat 1 arah.
16	[redacted]	Kekuatan<br>1. Menjelaskan tujuan sesi konseling,<br>2. Menggali informasi mengenai BB orang tua dan BB saat lahir untuk mendapatkan gambaran faktor yg berkontribusi terhadap BB,<br>3. Memperhatikan kenyamanan pasien untuk ditanya mengenai isu BB di saat pasien merupakan individu dengan overweight,<br>4. Sudah berusaha melakukan "evoking" dengan mengaitkan BB ke pandangan diri, komentar teman, pacar.<br>5. Setelah memberikan informasi ttg rencana perubahan, nakes menanyakan kembali apa yg pasien tangkap ttg seperti apa rencana yg ingin dilakukan.<br><br>Ruang Perbaikan<br>1. Sudah cukup banyak menggunakan pertanyaan terbuka dan ada sejumlah probing yang dilakukan. Namun bisa dilakukan probing lebih dalam mengenai kondisi kehidupan pasien (stress, kondisi rumah, dll). Nakes juga bisa lebih menggali usaha yang sudah dilakukan oleh pasien untuk meregulasi BB dan memberikan afirmasi apabila sudah ada yg dilakukan dgn baik,<br>2. Ada baiknya apabila alasan berubah dan rencana datangnya dari pasien terlebih dahulu, baru nakes yg menambahkan,<br>3. Lebih banyak menggunakan sistem TBT dalam memberikan penjelasan,<br>4. Mengingat klien sudah remaja akhir, mungkin bisa dilakukan sesi terpisah dengan pasien dan ortu,<br>5. Meski ortu ada bersama dengan pasien, namun belum melibatkan ortu dalam rencana perubahan (e.g. memberi info ttg keterlibatan spesifik yg bs dilakukan ortu seperti mengawasi makan, menyediakan makan sehat, dll).<br><br>Komentar Umum<br>Nakes terdengar santai, berusaha untuk engage dengan hal - hal yang relate dengan remaja, sudah ada upaya melakukan evoking dan membangun komunikasi 2 arah dengan pasien.
17	[redacted]	Kekuatan: 1) Menggali skrining kondisi pasien dengan lengkap 2) memotivasi klien untuk berubah dengan menekankan dampak negatif dari kondisi<br>Aspek yang perlu diperbaiki: Proses perubahan cenderung dipaksa dan diarahkan, lebih banyak memberikan pertanyaan terbuka tentang pendapat remaja tentang kondisinya<br>Komentar keseluruhan: Proses dilakukan dengan sistematis dan berusaha untuk

		memberikan informasi untuk mengarahkan pada perubahan
18	[redacted]	Kekuatan: 1) Detail dan sistematis 2)Memberikan informasi yang dibutuhkan<br>Aspek yang perlu diperbaiki: 1) Proses cenderung satu arah, dan tidak mengalir 2)Belum banyak menggali tentang kesulitan dan motivasi pasien untuk mengubah kondisinya<br>Komentar keseluruhan: Berjalan dengan sistematis namun terkesan satu arah
19	[redacted]	Kekuatan: 1) Menanyakan penghayatan pasien tentang kondisinya yang mendukung perubahan 2) melakukan skrining dengan lengkap<br>Area yang bisa diperbaiki: 1) Melakukan probing dengan pertanyaan terbuka untuk menggali kondisi dan kesulitan pasien remaja 2) Belum melibatkan remaja untuk membuat rencana perubahan<br>Komentar keseluruhan: Sudah dilakukan dengan sistematis, mencoba menggali kondisi dengan detail namun untuk perubahan masih cenderung diarahkan
20	[redacted]	Kekuatan:<br>1. Sudah menanyakan nama pasien terlebih dahulu baru dilanjutkan dengan orang tua,<br>2. Memperkenalkan diri dan gambaran sesi konseling<br>3. Sudah menanyakan terlebih dahulu apakah pasien mengetahui tentang obesitas, sebelum memberikan informasi apa itu obesitas. TBT<br><br>Ruang Perbaikan:<br>Nakes dapat meningkatkan engagement dengan pasien. Misalnya, saat memberikan informasi mengenai obesitas, kecepatan bicara dapat diperlambat dan penjelasan dipotong menjadi beberapa bagian sembari dikonfirmasi kepada pasien mengenai pemahamannya. Setelah proses pemberian informasi, dapat ditanyakan pula bagaimana pendapat pasien ttg info tersebut. Selain itu, saat menanyakan mengenai makanan kesukaan dan aktivitas fisik, dapat diprobing lebih lanjut dengan pertanyaan terbuka (co: bagaimana gambaran pola makan dan kebiasaan aktivitas harian), sehingga nakes bisa mendapatkan gambaran yang lebih utuh sebelum berpindah ke pertanyaan lainnya.<br><br>Komentar Keseluruhan:<br>Konseling masih belum menerapkan prinsip MI, pertanyaan yang diajukan belum menunjukkan engagement dengan pasien dan belum memperoleh gambaran yang utuh mengenai kondisi pasien. Selain itu, konseling masih di tahap screening saja sehingga belum sampai pada tahap perencanaan perubahan perilaku
21	[redacted]	*Belum diberi komentar oleh psikolog*
22	[redacted]	Kekuatan<br>1. Nakes sudah menggunakan sejumlah pertanyaan terbuka yang dilanjutkan dengan probing untuk mendapatkan gambaran yang lebih utuh mengenai pola makan dan pola hidup remaja. Saat mengajukan pertanyaan, nakes juga memberikan respon reflektif ("Oh iyaa", "Oh suka yaa..") sehingga percakapan menjadi lebih natural tidak seperti interogasi.<br>2. Setelah memberikan gambaran rencana perubahan, nakes mengonfirmasi kepada remaja mengenai pendapat remaja tentang rencana tersebut.<br><br>Ruang Perbaikan

		1. Pada bagian evoking nakes dapat lebih menggali alasan perubahan/ konsekuensi positif dari berubah/ konsekuensi negatif dari tidak berubah. Alasan – alasan ini bisa dikaitkan dengan keluhan yang dirasakan pasien terkait BB – nya.<br>2. Hal yang sama juga dapat diterapkan pada bagian planning, nakes dapat menanyakan kepada pasien, menurut pendapat pasien apa yang dapat dilakukan untuk membantu mengatur BB nya dari segi pola makan/ gaya hidup/ dll. Dengan demikian rencana perubahan lebih menggambarkan hasil kolaborasi pasien - nakes.<br>3. Di akhir bagian planning, setelah pemberian informasi mengenai pola makan/ hidup yang dapat diubah, nakes sudah mengonfirmasi kepada pasien apakah sudah cukup jelas atau belum. Namun akan lebih baik apabila nakes juga menanyakan pendapat pasien secara umum, kesanggupan pasien dalam menjalankan rencana, hambatan untuk menjalankan, dll.<br>4. Pemberian afirmasi bisa ditambahkan di dalam sesi konseling, misalnya karena pasien sudah memiliki kesadaran untuk mengelola BB dengan datang ke poli gizi.<br><br>Komentar Umum<br>Nakes sudah berusaha menerapkan prinsip MI dan melibatkan remaja di dalam sesi konseling. Sejumlah teknik MI dapat ditingkatkan penerapannya, khususnya bagian evoking dan planning. Pada kedua bagian tsb alasan berubah dan rencana perubahan sebaiknya dibantu untuk dimunculkan dari pasien agar lebih mengarah pada perubahan perilaku.
23	[redacted]	Kekuatan<br>1. Nakes berusaha untuk melakukan engaging dan evoking alasan perubahan untuk mengelola BB dengan cara menanyakan mengenai pandangan terhadap diri, body image, pentingnya memperhatikan penampilan untuk melengkapi kemampuan pasien yg sudah baik, dll.<br>2. Nakes terdengar antusias, tertarik, dan banyak mengetahui update tentang berbagai kegiatan pasien sehingga membantu untuk engage di dalam konseling.<br><br>Ruang Perbaikan<br>1. Sudah ada usaha untuk menggunakan pertanyaan terbuka, namun masih banyak penggunaan pertanyaan tertutup yg cenderung leading (co: "Tapi kamu di sekolah merasa nyaman kan ya?", "Di sekolah nggak pernah ada kekerasan apa - apa kan ya?"). Untungnya pasien dapat mengemukakan pendapatnya jika pertanyaan tertutup kurang sesuai dengan kondisinya.<br>2. Pada saat mengidentifikasi dan membuat perencanaan terkait perubahan pola makan/aktivitas, ada baiknya jika dimulai dengan menanyakan kepada pasien kira - kira aspek hidup apa yang menjadi masalah dan perubahan apa yg perlu dilakukan untuk mengelola BB. Proses penyusunan rencana juga bisa ditutup dengan menanyakan kepada pasien bagaimana kemampuannya untuk melaksanakan perubahan, apa saja hambatan yg mungkin muncul, dan bagaimana mengatasinya.<br><br>Komentar Umum<br>Penilaian diberikan untuk rekaman konseling yang dilakukan dengan pasien Ria. Nakes sudah banyak menerapkan prinsip2 MI di dalam konselingnya.
24	[redacted]	Kelebihan:<br>- Menggali kondisi dengan detail

		- Menunjukkan active listening dengan baik, menunggu jawaban remaja dengan sabar dan mendorong remaja untuk terlibat dan terbuka<br>- Melakukan evoking dengan baik sesuai dengan kondisi remaja (concern dengan fisik)<br><br>Area yang perlu dimprove:<br>Beberapa pertanyaan yang menanyakan kondisi di awal-awal agak kurang relevan dan cenderung diberikan dalam format pertanyaan tertutup<br><br>Komentar umum: Proses MI dilakukan dengan baik khususnya pada proses focusing dan evoking karena mempertimbangkan apa yang dianggap penting bagi remaja
25	[redacted]	Kekuatan:<br>1. Nakes banyak memberikan pertanyaan terbuka yang dilanjutkan dengan probing, sehingga membantu untuk memperoleh gambaran detil mengenai pola makan dan aktivitas fisik pasien,<br>2. Nakes sudah banyak memasukkan teknik mendengar reflektif, khususnya reflective content. Selain itu, di dalam memberikan refleksi, nakes juga sudah memberikan afirmasi pada hal - hal yang sudah baik dari pola makan/ aktivitas pasien.<br><br>Ruang Perbaikan<br>1. Nakes dapat memulai sesi dengan menanyakan kabar dan meminta pasien menceritakan keluhan subjektif yang ia rasakan terkait BBnya. Mungkin hal ini dapat membantu agar pasien lebih engage, sekaligus melakukan evoking alasan perubahan.<br>2. Pada sesi berikutnya dapat dipertimbangkan untuk mengadakan sesi terpisah antara pasien remaja dan ortu, sehingga pasien bisa lebih terbuka menceritakan masalah dari perspektifnya sekaligus lebih bisa dipancing opini pasien ttg perubahan apa yang dapat ia lakukan untuk mengelola BBnya.<br>3. Planning perubahan yang perlu dilakukan datangnya masih dari nakes; mungkin bisa ditanyakan kepada pasien apa yang menurutnya perlu diubah, bagaimana pandangannya terhadap perubahan yg ia sebutkan/ disarankan nakes, dan seberapa mampu ia melakukan perubahan tsb.<br><br>Komentar Umum<br>Nakes sudah menggunakan pertanyaan terbuka, probing, dan afirmasi. Namun sejumlah teknik MI yang perlu diperbanyak, misalnya engaging, evoking, dan memancing rencana perubahan yang datangnya dari pasien.
26	[redacted]	Kekuatan<br>1. Nakes terdengar ramah dan banyak mengingat fakta atau kebiasaan mengenai pasien, sehingga sesi terasa lebih personal dan membuat pasien merasa lebih diperhatikan,<br>2. Nakes berusaha memberikan penjelasan yang sederhana/ mudah dipahami mengenai pola makan/ porsi/ jenis makanan, aktivitas fisik, dll (co: dengan menggunakan analogi lampu hijau/kuning/merah),<br>3. Nakes cepat tanggap untuk membuat pasien remaja merasa tidak malu dan merasa tetap didengarkan, misalnya ketika ortu membahas mengenai ukuran

		dalaman pasien yang sudah tidak muat lagi, atau ketika ortu pasien terdengar kesal ketika ortu mengatakan bahwa ia marah - marah jika hp nya diambil > komentar nakes yg membantu: "oke nanti Mas Ryan sendiri ya yg atur hp kapan simpan HP"<br>4. Nakes berusaha untuk mengedukasi mengenai pentingnya proses, bahwa yang paling utama di tahap awal adalah perubahan pola perilaku, bukan BB.<br><br>Ruang Perbaikan:<br>1. Pada sesi berikutnya mungkin dapat dipertimbangkan untuk melaksanakan sesi konseling terpisah antara pasien remaja dan ortu, apalagi mengingat ortu pasien seringkali menjawab untuk anaknya dan pasien remaja sepertinya sudah bisa menjawab secara mandiri. Hal ini akan membantu pasien remaja untuk bisa berpendapat dan lebih engage dalam usahanya untuk berubah.<br>2. Nakes bisa lebih memperbanyak teknik MI di dalam sesi konseling, misalnya berusaha untuk mengajak pasien melihat dimana letak masalah sehingga BB kelebihan, apa keuntungan jika berubah (sehingga membantu pasien menemukan motivasi internal untuk berubah), apa yang dapat diubah, dan bagaimana kesiapan klien jika akan berubah.<br><br>Komentar Umum<br>Selama sesi nakes terdengar hangat, tanggap terhadap kondisi pasien, dan berusaha untuk memberikan penjelasan yang mudah dipahami. Namun, belum banyak teknik MI yang diterapkan dalam membuat rencana perubahan.
27	[redacted]	Kekuatan<br>1. Nakes terdengar ramah selama sesi,<br>2. Nakes sudah banyak melakukan pertanyaan terbuka yang dilanjutkan dengan probing, sudah melakukan refleksi konten sehingga beberapa kali berhasil mengonfirmasi kesalahpahaman, seperti pada saat membahas snack dan gorengan, serta gorengan sebagai pengganti makan siang.<br>3. Nakes melakukan rangkuman atas informasi yang diberikan oleh pasien (frekuensi makan dan aktivitas fisik) dan memberikan afirmasi atas hal - hal yang sudah baik dilakukan oleh pasien.<br>4. Saat membuat rencana perubahan, nakes tidak pernah lupa untuk menanyakan kemampuan pasien untuk mengerjakan rencana perubahan tsb.<br><br>Ruang Perbaikan:<br>1. Evoking: Nakes bisa menanyakan kepada pasien mengenai hal - hal apa yang menjadi alasan pasien untuk mengubah pola makan dan aktivitas fisiknya,<br>2. Planning: Nakes sudah cukup baik untuk menjelaskan rencan aperubahan dan mengonfirmasi kemampuan pasien untuk melakukan rencana, namun ada baiknya apabila rencana perubahan datangnya dari pasien terlebih dahulu sementara nakes lebih ke arah mendukung melalui pemberian informasi.<br><br>Komentar Umum<br>Konseling sudah dilakukan 2 arah, sudah banyak menerapkan prisnip MI.
28	[redacted]	Kelebihan:<br>- Melakukan skrining dan memberikan informasi yang dibutuhkan sesuai konteks<br>- Mencoba membuat planning perubahan

		Area yang perlu diimprove:<br>- Belum membangun rapport, langsung melakukan skrining dengan pertanyaan tertutup<br>- Banyak pertanyaan dengan format guessing (menebak dengan pertanyaan tertutup)<br><br>Komentar umum:<br>Proses sedikit loncat belum ada proses membangun rapport dengan perkenalan, menjelaskan proses apa yang sedang dilakukan, dan pertanyaan singkat terkait diri dan sehari-hari
29	[redacted]	Kekuatan<br>1. Ada upaya engaging dengan menanyakan kepada pasien mengenai persepsi pasien ttg BB nya<br>2. Ada upaya evoking alasan perubahan dengan memaparkan dampak dari BB berlebih di kehidupan sehari - hari (larinya lambat, badan terasa capek, dll), lalu menanyakan kepada pasien apakah ia merasakan hal tsb sebagai dampak dari BB berlebih.<br>3. Ada upaya untuk mendengar reflektif yang dilanjutkan dengan probing untuk memperdalam masalah,<br>4. Pemberian informasi sudah banyak menggunakan teknik TBT, khususnya saat dilakukan konseling terpisah dengan Ibu (co: Ibu sudah pernah dengar cara hitung TB/BB ideal? menurut Ibu apa yang berpengaruh terhadap BB nya?)<br>Ruang Perbaikan<br>1. Mungkin bisa dipertimbangkan untuk melakukan sesi terpisah dengan remaja agar remaja lebih banyak mendapat kesempatan berbicara sendiri,<br>2. Terkait dengan dampak dari BB berlebih mungkin bisa diarahkan agar datangnya dari pasien bukan dari nakes yang langsung memberikan. Hal ini bisa dilakukan sekaligus pada saat nakes menanyakan apa keluhan yg dirasakan sebagai akibat BB berlebih.<br>3. Pada saat memberikan informasi dengan teknik TBT, di akhir bisa dikonfirmasi kembali bagaimana pemahaman pasien/ ortu ttg informasi tsb.<br>4. Pada rekaman sesi ini belum terdapat upaya untuk membuat rencana konkrit dari segi pola makan/aktivitas/dll untuk menurunkan BB.<br>5. Pertanyaan sudah diajukan dgn prinsip pertanyaan terbuka dan ada probing lanjutan, namun belum terlalu menyentuh aspek HEAADSSS lainnya, seperti stress, kondisi rumah, dan sekolah.<br>Komentar Umum<br>Sudah ada beberapa prinsip MI yang diterapkan di dalam sesi, khususnya teknik engaging dan evoking. Upaya focusing juga sudah terlihat, meskipun baru muncul saat sesi dengan ortu saja ("menurut ibu apa yg berpengaruh terhadap kenaikan BB nya?"). Pada sesi ini belum terlihat adanya upaya planning perubahan pola makan/aktivitas yg konkrit.
30	[redacted]	Kekuatan Konseling:<br>1. Sudah menyapa orang tua dan anak,<br>2. Sudah menanyakan kepada anak apa yang menjadi masalah,<br>3. Informasi yang diberikan mengenai pola makan cukup jelas untuk diikuti oleh orang tua,

		Ruang Perbaikan:<br>Nakes perlu untuk melibatkan anak lebih banyak dalam interaksi 2 arah khususnya untuk mengetahui gambaran pola makan, aktivitas, dan kehidupan anak (poin - poin di dalam HEAADSSS), membantu anak mengidentifikasi faktor - faktor yang menyebabkan masalahnya, dan dalam merencanakan perubahan perilaku. Untuk membangun interaksi yang lebih bersifat 2 arah, nakes dapat memasukkan sejumlah teknik seperti memperbanyak probing, refleksi, afirmasi, dan menanyakan opini anak terkait topik/ rencana yang dibuat.<br><br>Komentar Keseluruhan:<br>Konseling masih bersifat 1 arah dan belum mengaplikasikan prinsip - prinsip MI, oleh karena itu masih dapat ditingkatkan lagi.
31	[redacted]	Kekuatan: 1) Melakukan planning dengan lebih spesifik 2) Menggali kondisi dengan cukup lengkap<br>Aspek yang perlu diperbaiki: Belum melakukan focusing, lebih mencoba menggali motivasi pasien untuk berubah, menggali kesulitan dengan pertanyaan terbuka<br>Komentar: Peserta melakukan keseluruhan sesi dengan lengkap menjelaskan informasi yang dibutuhkan, masih berubaha mengarahkan perubahan dan belum terlalu menggali motivasi dan melibatkan peserta untuk membuat rencana perubahan
32	[redacted]	Kekuatan<br>1. Nakes sudah menggunakan sejumlah pertanyaan terbuka yang dilanjutkan dengan probing untuk mendapatkan gambaran yang lebih utuh mengenai pola makan dan pola hidup remaja. Saat mengajukan pertanyaan, nakes juga memberikan respon reflektif dan afirmasi/ pujian untuk hal - hal yang sudah dilakukan dengan baik oleh remaja.<br>2. Dalam menyampaikan rangkuman, nakes tidak hanya menyampaikan hal - hal yang kurang, namun juga menyampaikan hal - hal yang sudah baik dan menyisipkan kata - kata afirmasi di dalamnya.<br>3. Saat memberikan penjelasan mengenai rencana perubahan perilaku, penjelasan sederhana, banyak memberikan contoh, dan dikaitkan dgn kondisi sehari2 pasien sehingga mudah dipahami.<br><br>Ruang Perbaikan<br>1. Pada bagian evoking nakes dapat lebih menggali alasan perubahan/ konsekuensi positif dari berubah/ konsekuensi negatif dari tidak berubah, sehingga alasan perubahan datangnya lebih banyak dari pasien bukan nakes.<br>2. Hal yang sama juga dapat diterapkan pada bagian planning, nakes dapat menanyakan kepada pasien, menurut pendapat pasien apa yang dapat dilakukan untuk membantu mengatur BB nya dari segi pola makan/ gaya hidup/ dll. Dengan demikian rencana perubahan lebih menggambarkan hasil kolaborasi. Di akhir bagian perencanaan bisa ditanyakan, bagaimana pendapat/ kesanggupan pasien dan ortu utk menjalankan rencana yang dibuat<br>3. Pemberian informasi dapat lebih banyak menggunakan prinsip TBT dengan menanyakan dulu mengenai hal apa yang diketahui oleh pasien/ ortu pasien dan di akhir menanyakan bagaimana pendapat pasien ttg informasi yang diberikan.<br>4. Mungkin pada sesi selanjutnya dapat dipertimbangkan untuk mengajak pasien remaja berbicara sendiri tanpa didampingi orang tua agar pasien bisa lebih

		terbuka.<br><br>Komentar Umum<br>Nakes sudah menerapkan prinsip MI dengan cukup baik dan berusaha melibatkan remaja di dalam sesi konseling. Nakes terdengar antusias, ramah, dan berusaha memberikan pujian untuk hal - hal yang sudah baik dilakukan oleh pasien maupun orang tua, sehingga hal ini juga dapat memotivasi pasien. Sejumlah teknik MI dapat ditingkatkan penerapannya, khususnya bagian evoking dan planning.
33	[redacted]	Kekuatan:<br>1. Nakes sudah berusaha melibatkan anak meskipun pasien masih berusia 10 tahun dan terdengar sedang asik bermain dengan gadgetnya. Salah satunya bentuk pelibatan anak adalah dengan mendahulukan bertanya kepada anak dan mengonfirmasi informasi kepada orang tua,<br>2. Nakes sudah memberikan sejumlah pertanyaan terbuka dan probin yang cukup detil untuk memperoleh gambaran mengenai pola makan, menu, dan aktivitas anak.<br><br>Ruang Perbaikan:<br>Pada saat memberikan informasi mengenai pola makan dan aktivitas, mungkin dapat ditanyakan lebih dahulu usaha - usaha apa yang telah dilakukan untuk membantu menurunkan BB, baru setelah itu diberi masukan. Selain itu, saat membuat planning bisa ditanyakan ide/ masukan dari pasien/ ortu ttg apa yang bisa direncanakan dan bagaimana tanggapan pasien/ ortu terkait rencan ayang diajukan nakes (co: apakah memungkinkan menjalankan rencana, apa hambatannya, dll).<br><br>Komentar Umum:<br>Penerapan prinsip MI di dalam konseling dapat lebih ditingkatkan, khususnya di bagian engaging, focusing, evoking. Namun demikian, mengingat pasien yang usianya masih sangat muda dan sedang bermain game selama sesi, nakes sudah menunjukkan usaha cukup baik untuk tetap melibatkan pasien.

B

Pasca pelatihan

DAFTAR KOMENTAR KELOMPOK B - Unggah Audio Kasus 1 - Pasca Pelatihan

No	Nama Depan	Komentar
1	[redacted]	kekuatan: sudah sangat baik dalam menerapkan teknik-teknik MI, teknik komunikasi juga dilakukan lancar dengan menggunakan pertanyaan terbuka, afirmasi yang cukup dan mendengarkan reflektif<br>ruang perbaikan: dilatih terus dan diperlancar saja sehingga terbiasa dan komunikasi berjalan dengan mengalir, libatkan juga orang tua untuk mendukung progres anak<br>keseluruhan: konseling berjalan sangat baik, mengalir dan fokus pada masalah, pasien dilibatkan penuh sehingga komunikasi berjalan dua arah dan saling mendukung
2	[redacted]	Kekuatan:<br>- Sudah cukup baik dalam memberikan pertanyaan-pertanyaan terbuka yang dilanjut dengan probing sehingga membantu pasien remaja lebih banyak bercerita<br>- Sudah baik dalam memberikan afirmasi pada upaya yang dilakukan oleh pasien remaja<br>- Sudah menerapkan proses MI dalam konseling dengan baik, melibatkan pasien remaja, membantu pasien fokus pada perubahan perilaku spesifik, dan mengajak remaja membuat perencanaan sendiri<br><br>Ruang perbaikan:<br>- Perlu meminta sesi remaja untuk berbicara sendiri agar lebih leluasa dan nyaman menceritakan kondisinya<br>- Bisa lebih menggali motivasi internal pasien remaja untuk menurunkan BB-nya<br>- Bisa lebih menggali informasi terkait kondisi pasien (screening HEAADSSS) secara lebih lengkap agar membantu remaja mengidentifikasi hambatan dan pendukungnya untuk mengubah perilaku/pola hidupnya<br><br>Komentar keseluruhan:<br>Secara umum sudah melakukan konseling dengan cukup baik, melibatkan pasien remaja dan memberikan afirmasi positif pada upaya/rencana yang dibuat oleh pasien remaja, namun karena tidak meminta sesi remaja sendiri sehingga orang tua juga lebih banyak mendominasi jawaban pasien
3	[redacted]	kekuatan: teknik MI sudah dilakukan dengan baik dan konsisten, meskipun pasien tidak merasa terganggu dengan berat badannya namun konselor tetap konsisten untuk menyadarkan masalahnya, teknik komunikasi seperti pertanyaan terbuka, mendengar reflektif dan memberi afirmasi juga sudah diberikan sesuai porsinya<br>ruang perbaikan: bisa pancing kesadaran pasien dengan memberikan informasi terkait dampak dari berat badan yang tidak ideal<br>keseluruhan: konseling berjalan dengan sangat mengalir dan mampu membuat pasien nyaman untuk bercerita, konselot sangat fokus menggali masalah dan membantu pasien
4	[redacted]	Kekuatan:<br>- Sudah melakukan teknik-teknik konseling dengan baik dengan menggunakan pertanyaan terbuka, mendengar reflektif, dan memberikan afirmasi yang sesuai pada kondisi pasien remaja

		- Sudah menggali dan memberi afirmasi pada motivasi yang disampaikan oleh pasien remaja untuk mengubah perilaku pola makannya<br>- Sudah baik dalam mengajak pasien remaja untuk menyusun rencana pola makan dan aktivitas fisik yang sesuai dan bisa dilakukan oleh pasien sendiri<br><br>Ruang perbaikan:<br>- Bisa lebih menggali kondisi pasien remaja melalui screening HEAADSSS agar lebih mendapat gambaran keadaan pasien dan membantu pasien mengidentifikasi area masalah<br><br>Komentar keseluruhan:<br>Secara umum sudah melakukan konseling dengan proses MI dengan baik, melibatkan penuh pasien remaja dalam berdiskusi secara dua arah terkait perubahan perilakunya, terutama dalam menyusun rencana pola makan dan aktivitas yang bisa dilakukan sendiri oleh remaja. Nakes juga membantu pasien remaja untuk mengidentifikasi hambatan dan pendukung dalam mengubah perilakunya.
5	[redacted]	Kekuatan:<br>- Sudah melibatkan pasien remaja dalam sesinya untuk berbicara sendiri dan menceritakan kondisinya<br>- Sudah berupaya menerapkan proses MI dalam konseling<br><br>Ruang perbaikan:<br>- Bisa lebih banyak memberikan pertanyaan yg terbuka dibandingkan yg hanya memberikan kesempatan jawaban ya/tidak<br>- Bisa lebih menggali motivasi internal dari pasien remaja untuk mengubah pola makan dan aktivitasnya<br>- Bisa lebih melibatkan pasien remaja untuk membuat perencanaannya sendiri agar tidak hanya mengikuti arahan dari nakes<br><br>Komentar keseluruhan:<br>Secara umum nakes sudah berupaya melibatkan pasien remaja dalam proses konseling menggunakan MI dengan cukup baik, namun masih lebih banyak diarahkan oleh nakes dan masih sering menggunakan pertanyaan tertutup atau leading.
6	[redacted]	Kekuatan:<br>- Sudah memberikan afirmasi atas perubahan perilaku pola makan dan olahraga yang dilakukan oleh pasien<br>- Sudah mendiskusikan dukungan yang bisa diberikan orangtua untuk mengawasi perubahan yang dilakukan oleh pasien remaja<br><br>Ruang perbaikan:<br>- Sudah memberikan pertanyaan terbuka, tapi masih lebih banyak memberikan pertanyaan tertutup ya/tidak<br>- Masih perlu lebih banyak melibatkan pasien dalam membuat perencanaan perubahan ke depannya, dan menggali motivasi diri pasien yang mendorongnya untuk berubah

2

		Komentar keseluruhan:<br>Secara umum rekaman/sesi cukup singkat dan lebih bersifat follow up perkembangan pasien sejak sesi sebelumnya. Dalam sesi sudah berupaya memberikan afirmasi yang sesuai atas perkembangan baik dan upaya dari pasien. Namun masih bisa lebih banyak menggali kondisi/kendala yang dialami pasien dalam mengubah pola makan dan aktivitasnya, serta motivasi diri pasien untuk berubah.
7	[redacted]	kekuatan: sudah menggunakan cukup banyak pertanyaan terbuka, memberikan afirmasi dan mendengarkan reflektif<br>ruang perbaikan: konseling terdengar lebih banyak melakukan follow up saja sehingga tidak terlihat teknik MI dilakukan, kedepannya dapat terus melatih penggunaan teknik MI dengan lebih lancar<br>Keseluruhan: konselor dapat membuat pasien nyaman bercerita dan menyampaikan planningnya
8	[redacted]	gugur
9	[redacted]	Kekuatan:<br>- Sudah melakukan teknik-teknik konseling dengan cukup baik, menggunakan pertanyaan-pertanyaan terbuka dan merefleksikan konten pembicaraan pasien remaja<br>- Melibatkan pasien remaja dalam diskusi 2 arah terkait permasalahan yang dialami, dukungan, serta hambatan untuk mengubah perilaku pola makannya<br>- Sudah melibatkan remaja dalam menyusun perencanaan perubahan pola makan<br><br>Ruang perbaikan:<br>- Perlu meminta sesi remaja berbicara sendiri agar remaja lebih leluasa dan nyaman menceritakan kondisi dan kesulitannya<br>- Bisa lebih menggali motivasi internal pasien remaja untuk menurunkan berat badan, serta memberikan afirmasi positif yang relevan untuk menguatkan keinginannya mengubah perilaku pola makan dan aktivitas fisik<br><br>Komentar keseluruhan:<br>Secara umum sudah menunjukkan upaya menerapkan teknik-teknik konseling, serta menerapkan proses MI seperti engaging dan planning, namun masih perlu melakukan evoking dan focusing lebih dalam agar remaja termotivasi kuat dari dalam dirinya dan mampu memilah perubahan perilaku yang ingin dilakukan
10	Yunita	kekuatan: sudah cukup banyak pertanyaan terbuka yang diajukan, banyak mendengar reflektif dan sudah banyak upaya yang dilakukan untuk menggali dan menyadarkan pasien akan masalahnya evoking<br>ruang perbaikan: bisa lebih banyak menggali apa yang menjadi hambatan pasien, perbanyak afirmasi terhadap hal-hal yang sudah baik, lebih banyak memunculkan planning dari pasien sendiri dan memancing pasien untuk lebih banyak bercerita<br>keseluruhan: konseling berjalan mengalir, konselor sudah cukup banyak memancing agar pasien menyadari kondisi dan masalah pada dirinya
11	[redacted]	kekuatan: pertanyaan terbuka dilakukan dengan baik dan dilanjutkan probing, sudah cukup banyak memberikan afirmasi dan mendengarkan reflektif, mampu memancing pasien untuk bercerita dan terbuka dengan kondisinya, teknik MI

		seperti engaging dan focusing sudah dilakukan dengan baik<br>ruang perbaikan: latih lagi kemampuan planning dalam teknik MI, upayakan lebih banyak memancing planning dari kesanggupan pasien dan mengurangi saran-saran yang sifatnya mengarahkan<br>keseluruhan: konseling berjalan dengan sangat mengalir, terlihat pasien nyaman dan dapat terbuka dengan konselor, konselor juga sangat fokus dan memperhatikan pasien
12	[redacted]	Kekuatan:<br>- Banyak memberikan pertanyaan terbuka dan mendengar reflektif sehingga pasien bisa leluasa menceritakan masalah/kendalanya dalam menurunkan berat badan<br>- Sudah menerapkan MI dalam konseling dengan baik, melibatkan pasien, menggali motivasi internal pasien, dan mengajak pasien berdiskusi dalam membuat perencanaan<br><br>Ruang perbaikan:<br>- Masih perlu menggali lebih banyak change talk (omongan dari pasien tentang keinginannya berubah) ketika pasien menyatakan kesulitan untuk mengubah kebiasaan ngemil<br><br>Komentar keseluruhan:<br>Secara umum sudah baik dalam menerapkan MI dan memberikan konseling pada remaja, memberikan pertanyaan terbuka dan mendengar reflektif sehingga pasien nyaman bercerita tentang kendalanya.
13	[redacted]	Kekuatan:<br>- Sudah baik dalam melibatkan pasien remaja selama proses konseling dengan menggunakan pertanyaan terbuka dan menggali motivasi internal pasien untuk menurunkan BB<br>- Sudah melakukan proses MI dalam konseling dengan cukup baik, terutama dalam aspek engaging dan planning<br><br>Ruang perbaikan:<br>- Bisa lebih banyak menggali kondisi pasien remaja untuk membantu remaja mengidentifikasi pendukung dan hambatan dalam mengubah perilakunya<br>- Bisa lebih banyak memberikan afirmasi pada upaya yang sudah dilakukan oleh pasien untuk mendorong motivasi pasien mempertahankan perubahan perilakunya<br><br>Komentar keseluruhan:<br>Secara umum sudah melakukan konseling dengan teknik-teknik MI dengan baik, melibatkan pasien remaja dalam diskusi secara 2 arah, dan memberi informasi yang relevan untuk membantu pasien melakukan perubahan perilaku
14	[redacted]	Kekuatan:<br>- Sudah meminta sesi remaja sendiri sehingga membuat pasien remaja nyaman dan leluasa menceritakan kondisinya<br>- Sudah baik dalam melakukan konseling dengan menggunakan pertanyaan-pertanyaan terbuka, mendengar reflektif, memberi afirmasi dan memotivasi dengan sesuai

		- Sudah menerapkan proses MI dengan cukup baik, terutama membantu pasien remaja fokus pada area perilaku yang ingin diubah (aktivitas fisik) dan mengajak remaja untuk menyusun rencana dengan diskusi 2 arah<br><br>Ruang perbaikan:<br>- Bisa lebih banyak menggali kondisi pasien remaja untuk mendapatkan gambaran kondisi psikososialnya, dan membantu remaja mengidentifikasi hambatan dan pendukungnya dalam mengubah perilakunya<br><br>Komentar keseluruhan:<br>Secara umum sudah melakukan konseling perubahan perilaku menggunakan MI dengan baik, sudah membina rapport dengan pasien remaja dengan baik dan menggunakan pertanyaan terbuka dan afirmasi yang sesuai sehingga membantu pasien remaja leluasa menceritakan kondisinya. Nakes juga sudah memberikan informasi dengan teknik TBT lengkap dan relevan untuk membantu pasien mengubah perilakunya.
15	[redacted]	Kekuatan:<br>- Sudah cukup baik dalam menggali informasi terkait pola makan dan aktivitas pasien<br>- Sudah ada upaya melakukan proses MI, namun masih perlu menggali motivasi diri pasien untuk menurunkan BB dan meningkatkan lagi keterlibatan pasien dalam berdiskusi<br><br>Ruang perbaikan:<br>- Masih perlu meningkatkan keterlibatan pasien dalam berdiskusi<br>- Perlu menggali motivasi diri pasien untuk berubah/menurunkan BB<br><br>Komentar keseluruhan:<br>Secara umum sudah menunjukkan upaya melakukan proses MI, namun masih perlu meningkatkan keterlibatan pasien dalam diskusi, bisa dengan lebih banyak memberikan pertanyaan-pertanyaan terbuka dan juga afirmasi positif atas upaya yang dilakukan pasien untuk mengubah pola makan dan aktivitasnya
16	[redacted]	Kekuatan:<br>- Sudah cukup baik dalam melakukan konseling dengan menggunakan pertanyaan-pertanyaan terbuka, dan melakukan proses MI<br>- Sudah berupaya menggali motivasi internal pasien remaja untuk menurunkan BB dan melakukan perubahan pada pola hidup (aktivitas fisik)<br><br>Ruang perbaikan:<br>- Perlu memberikan sesi terpisah dengan pasien remaja untuk meningkatkan pelibatan pasien<br><br>Komentar keseluruhan:<br>Secara umum nakes sudah cukup baik dalam melakukan proses MI dan memberikan pertanyaan-pertanyaan terbuka pada pasien remaja, namun jawaban masih lebih banyak didominasi oleh orangtua pasien
17	[redacted]	kekuatan: teknik MI sudah cukup banyak dilakuakn dan konsisten, konselor sangat berusaha untuk fokus pada masalah dan memancing motivasi dan planning

		perubahan perilaku dari pasien, afirmasi dan mendengarkan reflektif juga sudah dilakukan sesuai porsinya<br>ruang perbaikan: pertanyaan terbuka sudah dilakukan namun seringkali ditutup kembali dengan pertanyaan tertutup sehingga pasien tidak terpancing untuk memberikan jawaban yang lebih banyak<br>keseluruhan: terlihat konselor sangat fokus pada penumbuhan kesadaran akan masalah yang dimiliki pasien, konseling berjalan dengan mengalir dan terus konsisten melibatkan pasien selama sesi konseling
18	[redacted]	kekuatan: sudah cukup banyak pertanyaan terbuka yang diberikan, upaya menggunakan teknik MI sudah baik, ada afirmasi dan mendengarkan reflektif selama konseling, memperhatikan berbagai aspek dari sisi pasien maupun orang tua pasien<br>ruang perbaikan: lebih banyak memberikan afirmasi dan dukungan terhadap hambatan yang dihadapi pasien, lebih banyak memancing planning dari pasien sendiri dan mengurangi arahan yang langsung, terus berlatih agar konseling dapat berjalan lebih mengalir<br>keseluruhan: secara umum sudah baik dalam melakukan MI, namun masih cukup banyak juga saran langsung yang diberikan sehingga terkesan seperti mengarahkan
19	[redacted]	Kekuatan:<br>- Sudah cukup baik dalam menggunakan teknik pertanyaan-pertanyaan terbuka untuk menggali motivasi pasien remaja untuk menurunkan BB<br>- Sudah cukup baik dalam melakukan MI dan melibatkan langsung pasien remaja dalam membuat rencana perubahan perilaku terkait aktivitas dan pola makan<br><br>Ruang perbaikan:<br>- Bisa menggali motivasi internal pasien untuk menurunkan BB secara lebih dalam, terlebih karena sudah mendapatkan info terkait pengalaman pasien dibully akibat BB nya<br>- Bisa lebih banyak memberikan afirmasi pada upaya atau perencanaan yang sudah dibuat oleh pasien untuk lebih mendorong motivasinya untuk berubah<br><br>Komentar keseluruhan:<br>Secara umum sudah cukup baik dalam memberikan konseling perubahan perilaku dengan teknik MI, dan melibatkan penuh pasien remaja dan peran orang tua
20	[redacted]	kekuatan: sudah baik dalam menerapkan teknik-teknik MI dan teknik komunikasi yang baik seperti memberi afirmasi, mendengarkan reflekrif, dan menggunakan pertanyaan terbuka<br>ruang perbaikan: dalam upaya planning usahakan lebih banyak muncul dari pasien sendiri dan libatkan orang tua dalam mendukung program diet anak<br>keseluruhan: konseling berjalan dengan mengalir, konselor dapat membuat pasien nyaman untuk bercerita dan dapat membantu pasien untuk fokus pada masalah serta menemukan solusinya
21	[redacted]	kekuatan: sudah banyak pertanyaan terbuka yang diajukan, teknik konseling dengan MI juga sudah cukup konsisten diterapkan, mendengar reflektif dan afirmasi juga sudah banyak dilakukan<br>Ruang perbaikan: percakapan terdengar agak kaku, bisa lakukan komunikasi yang lebih santai dan percaya diri sehingga percakapan pun akan terasa mengalir<br>keseluruhan: konseling berjalan dengan lancar, pasien bisa menceritakan dengan

		nyaman dan cukup terbuka
22	[redacted]	kekuatan: sudah menggunakan teknik MI dengan baik dan konsisten, teknik komunikasi seperti pertanyaan terbuka, mendengar reflektif dan memberi afirmasi juga sudah dilakukan sesuai porsinya<br>ruang perbaikan: terus dilatih dan sering menggunakan teknik MI sehingga semakin lancar dan terbiasa<br>keseluruhan: konseling berjalan dengan sangat mengalir dan mampu membuat pasien sadar akan kondisi dirinya, memunculkan motivasi dari dalam pasien sendiri dan memancing rencana perubahan perilaku dari pasien
23	[redacted]	Kekuatan:<br>- Sudah melakukan prosedur pembukaan konseling dengan lengkap dan baik<br>- Sudah berupaya melakukan teknik-teknik MI dalam melakukan konseling dengan memfokuskan pada pasien remaja selama sesi<br><br>Ruang perbaikan:<br>- Bisa lebih banyak memberikan pertanyaan terbuka untuk dijawab oleh pasien agar pasien leluasa menceritakan kondisinya<br>- Bisa lebih banyak memberikan afirmasi pada upaya yg sudah dilakukan oleh pasien agar lebih termotivasi untuk mengubah pola makan dan mendorong pasien lebih terbuka untuk bercerita<br>- Bisa lebih melibatkan pasien dalam membuat perencanaan perubahan perilaku agar tidak hanya mengarahkan pasien saja, tapi juga meminta ide dari pasien sendiri untuk membuat rencana sendiri<br><br>Komentar keseluruhan:<br>Secara umum sudah menunjukkan upaya untuk melakukan teknik-teknik konseling dengan proses MI, namun perlu berhati-hati dalam memberikan pertanyaan yang mengarahkan pada jawaban ya/tidak atau jawaban tertentu. Nakes juga sudah cukup baik dalam menggali motivasi pasien atas keinginannya untuk menurunkan BB, namun masih perlu mendorong pasien untuk menyadari motivasi internalnya sendiri untuk mengubah pola makan dan aktivitas fisiknya
24	[redacted]	kekuatan: pertanyaan terbuka sudah dilakukan dengan baik disertai dengan probing lanjutan, seluruh teknik MI juga sudah dilakukan secara konsisten, afirmasi dan mendengar reflektif diberikan sesuai porsinya<br>ruang perbaikan: dilatih lebih sering dalam menggunakan teknik MI sehingga percakapan pun dapat berjalan dengan lebih santai dan mengalir<br>keseluruhan: konseling dapat berjalan dengan lancar dan konselor dapat membuat pasien bercerita lebih dalam serta memancing pasien untuk terus memperbaiki pola hidupnya
25	[redacted]	kekuatan: sudah banyak menggunakan pertanyaan terbuka, memberi afirmasi yang sesuai dan mendengarkan reflektif, ada upaya untuk menggunakan teknik-teknik MI<br>ruang perbaikan: lebih fokus pada apa yang menjadi masalah bagi pasien, upayakan memunculkan motivasi dari diri pasien sendiri untuk menurunkan berat badan, kurangi pemberian saran yang mengarahkan<br>keseluruhan: konselor mampu membuat pasien nyaman dalam bercerita dan cukup banyak membantu kebingungan orang tua pasien serta memberikan afirmasi kepada pasien

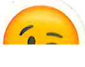

26	[redacted]	kekuatan: sudah cukup banyak menggunakan pertanyaan terbuka sehingga tergali pengetahuan dan masalah yang pasien miliki, sudah cukup banyak upaya-upaya yang dilakukan untuk menyadarkan masalah yang dimiliki dan upaya yang bisa dilakukan<br>ruang perbaikan: upayakan memberikan afirmasi pada hal-hal baik yang sudah pasien bisa dan ingin lakukan, lebih banyak memunculkan planning dari pasien sendiri dan lebih fokuskan pada masalah yang pasien rasa mengganggu bagi dirinya<br>keseluruhan: konseling berjalan cukup mengalir, konselor dapat membuat pasien nyaman bercerita dan membantu pasien untuk lebih sadar akan kondisi dirinya
27	[redacted]	kekuatan: sudah cukup banyak menggunakan pertanyaan terbuka sehingga tergali pengetahuan dan masalah yang pasien miliki, sudah cukup banyak upaya-upaya yang dilakukan untuk menyadarkan masalah yang dimiliki dan upaya yang bisa dilakukan<br>ruang perbaikan: upayakan memberikan afirmasi pada hal-hal baik yang sudah pasien bisa dan ingin lakukan, lebih banyak memunculkan planning dari pasien sendiri dan lebih fokuskan pada masalah yang pasien rasa mengganggu bagi dirinya<br>keseluruhan: konseling berjalan cukup mengalir, konselor dapat membuat pasien nyaman bercerita dan membantu pasien untuk lebih sadar akan kondisi dirinya
28	[redacted]	Kekuatan:<br>- Sudah menggali informasi terkait kondisi keseharian remaja dengan lengkap yang dapat mendukung atau menghambat perubahan perilakunya<br>- Sudah baik dalam melakukan teknik-teknik konseling seperti memberi pertanyaan terbuka dan mendengar reflektif sehingga membuat remaja terdengar cukup nyaman untuk bercerita lebih lanjut<br>- Sudah cukup baik dalam menerapkan proses MI dengan menggali motivasi diri pasien remaja untuk mengubah perilaku (pola makan dan aktivitas fisik)<br>Ruang perbaikan:<br>- Bisa lebih banyak memberikan pertanyaan terbuka dan membiarkan remaja menjawab dengan leluasa terlebih dahulu, saat ini sudah banyak upaya memberikan pertanyaan terbuka namun beberapa kali dilanjut dengan pertanyaan tertutup atau agak mengarah (leading)<br><br>- Bisa lebih fokus dalam berdiskusi dengan pasien remaja untuk memetakan area masalahnya (aspek focusing) dalam masalah BB berlebihnya, saat ini masih cenderung meluas pembahasan masalahnya<br>Komentar keseluruhan:<br>Secara umum sudah cukup baik dalam memberikan konseling dengan menerapkan teknik MI, namun masih perlu membantu remaja untuk fokus pada area paling bermasalah yang berkontribusi pada berat badannya, karena saat ini pertanyaan untuk diskusinya masih cenderung luas dan secara umum saja, belum spesifik pada apa yang menjadi hambatan atau dukungan remaja. Selain itu, masih perlu melibatkan remaja dalam diskusi 2 arah dalam menyusun perencanaan yang bisa dilakukan oleh remaja sendiri.
29	[redacted]	kekuatan: sudah menggunakan banyak pertanyaan terbuka dan dilanjutkan probing, sudah konsisten menggunakan teknik-teknik MI sehingga konseling berjalan dengan fokus, afirmasi sudah sesuai konteks dan membuat pasien

		nyaman, percakapan juga berjalan dengan mengalir<br>Ruang perbaikan: secara teknik MI sudah cukup baik, mungkin hanya dalam hal teknis saja seperti meminta remaja berbicara sendiri dan mendiskusikan peran orang tua dalam mendukung keberhasilan anaknya<br>keseluruhan: konseling berjalan dengan baik dan mengalir, pasien terlihat nyaman dan dapat bercerita dengan terbuka
30	[redacted]	Kekuatan:<br>- Sudah cukup baik dalam melakukan teknik-teknik konseling seperti menggunakan pertanyaan terbuka, mendengar reflektif, dan memberi afirmasi, namun sesi konseling lebih banyak didominasi oleh orang tua<br>- Sudah berupaya menerapkan teknik MI untuk menggali motivasi internal pasien, melibatkan pasien dalam memfokuskan masalahnya, dan membuat perencanaan perubahan perilaku<br>- Sudah memberikan informasi yang relevan dan lengkap yang dapat membantu pasien remaja untuk mengubah perilakunya<br><br>Ruang perbaikan:<br>- Sebaiknya meminta sesi remaja berbicara sendiri agar remaja lebih nyaman dan leluasa untuk menceritakan kondisinya<br>- Bisa lebih banyak memberikan afirmasi pada saat pasien remaja menjawab untuk dirinya sendiri agar lebih termotivasi untuk menjawab nakes<br><br>Komentar keseluruhan:<br>Secara umum nakes sudah berupaya untuk melibatkan pasien remaja dalam sesi konseling, namun karena tidak meminta sesi remaja berbicara sendiri sehingga orang tua lebih mendominasi dalam menjawab pertanyaan nakes. Nakes sendiri sudah berupaya untuk menerapkan proses MI dalam konseling dengan cukup baik, namun keterlibatan pasien remaja sendiri masih kurang.
31	[redacted]	Kekuatan:<br>- Melakukan sesi sendiri dengan remaja, menjamin kerahasiaan, dan memberikan pertanyaan-pertanyaan terbuka yang membuat remaja merasa lebih nyaman dan leluasa menceritakan masalahnya<br>- Menggali kondisi (screening HEAADSSS) remaja dengan cukup mendalam sehingga membantu memahami kondisi remaja<br>- Sudah baik dalam menerapkan teknik-teknik MI dalam konseling, terutama dalam proses engaging, melibatkan remaja dalam diskusi 2 arah, evoking dengan menggunakan skala dan menanyakan dampak negatif dari BB berlebih pasien remaja, dan membantu pasien fokus dalam area masalah yang ingin diubah<br>- Sudah baik dalam mengajak pasien remaja mendiskusikan secara 2 arah untuk menyusun perencanaan perubahan pola makan<br>Ruang perbaikan:<br>- Bisa melibatkan peran orangtua dalam sesi-sesi berikutnya, untuk menekankan dukungan yang bisa diberi orangtua dalam perubahan perilaku remaja untuk menurunkan BB<br>Komentar keseluruhan:<br>Nakes sudah baik dalam menerapkan teknik-teknik konseling seperti memberikan pertanyaan terbuka, mendengar reflektif, dan mengonfirmasi pada remaja, sehingga membuat remaja terdengar cukup terbuka dan nyaman menceritakan

		tentang dirinya. Nakes juga sudah baik dalam menerapkan teknik MI dalam keseluruhan proses konseling.
32	[redacted]	Kekuatan:<br>- Sudah berupaya untuk melibatkan pasien remaja dalam diskusi 2 arah menggunakan pertanyaan terbuka yang ditujukan pada remaja, dan berupaya menenangkan remaja agar nyaman bercerita<br>- Sudah berupaya melakukan proses MI dalam konseling seperti melibatkan remaja dalam diskusi, berusaha menggali motivasi diri remaja, memfokuskan area masalah, dan menyusun perencanaan<br>- Memberikan informasi yang lengkap terkait pola makan sehat yang dapat membantu pasien remaja menyusun rencana perubahan perilakunya<br>Ruang perbaikan:<br>- Perlu meminta sesi terpisah dengan remaja sendiri agar remaja lebih nyaman dan leluasa menceritakan masalahnya<br>- Perlu lebih melibatkan pasien dalam diskusi yang bersifat 2 arah, menggunakan lebih banyak pertanyaan-pertanyaan terbuka untuk menggali keinginan pasien remaja untuk mengubah perilakunya → evoking<br>- Perlu lebih melibatkan pasien untuk menyusun rencana perubahan perilakunya untuk mengurangi pola makan atau meningkatkan aktivitas fisiknya, sesuai dengan kondisi dan kemampuan remaja<br>Komentar keseluruhan:<br>Secara umum nakes sudah menunjukkan upaya yang cukup baik dalam melakukan konseling dengan teknik-teknik MI, namun karena tidak ada sesi terpisah dengan remaja sendiri, sesi terdengar cenderung didominasi oleh orangtua yang mendampingi.
