# Supplementary material for: Adolescent weight management counseling: The effectiveness of an online training program for primary healthcare professionals in Indonesia
Source: PLoS One. 2025 Feb 10;20(2):e0315770. doi: 10.1371/journal.pone.0315770 (PMC11809861; doi:10.1371/journal.pone.0315770)
Supplement: Attachment — Submitted filename: PONE-D-23-35751_reviewer.pdf [file pone.0315770.s003.pdf]

PLOS ONE

Adolescent weight management counseling: the effectiveness of an online training program for primary healthcare professionals in Indonesia

--Manuscript Draft--

Manuscript Number:	PONE-D-23-35751
Article Type:	Research Article
Full Title:	Adolescent weight management counseling: the effectiveness of an online training program for primary healthcare professionals in Indonesia
Short Title:	The effectiveness of an online training for health workforce on adolescent weight management counseling.
Corresponding Author:	Fransisca Handy Agung<br>Universitas Pelita Harapan Faculty of Medicine<br>Jakarta Barat, Jakarta INDONESIA
Keywords:	adolescents, behavior change, counseling skills, healthy lifestyle, internet-based training, obesity, weight management
Abstract:	Background: Overweight and obesity are growing public health concerns globally for which innovative prevention and care delivery efforts are required. We recently developed a web-based training program to improve the quality of health professionals' weight management counseling of adolescents in Indonesia. Having previously confirmed its acceptability, this study aimed to measure the effectiveness of the program through a randomized controlled trial.<br>Methods: We recruited 64 primary healthcare professionals from 17 provinces across Indonesia who were randomized to participate in a 4-week online training (intervention group [IG, n=32] or a waitlist control group [CG, n=32]. Using active learning approaches, the training program focused on adolescent development, psychosocial assessment, motivational interviewing (MI), and parent engagement. Participants in each arm were asked to record two counseling sessions with adolescents. These were objectively rated by trained psychologists using a validated tool, and also by qualitative assessments of counseling quality. In both groups, the first recorded counseling session occurred before the training. The second recording occurred after the training for IG participants, but not for CG participants.<br>Results: IG participants demonstrated significant improvements in knowledge and counseling skills ($p<0.001$, t-test). This included improvements in introductory remarks, quality of psychosocial assessment, and MI skills. There was no change in the extent of parental involvement. The MI training successfully oriented the counseling sessions towards a more collaborative and participatory conversation for supporting behavioral change.<br>Conclusion: This novel online training program improved the knowledge and counseling skills of Indonesian primary healthcare professionals. Greater emphasis on engaging parents and more guidance on conducting telehealth counseling may improve parental involvement in future iterations.
Order of Authors:	Fransisca Handy Agung
	Rini Sekartini, Prof, MD, PhD
	Nani Sudarsono, MD, PhD
	Aryono Hendarto, Prof, MD, PhD
	Retno Asti Werdhani, MD, PhD
	Meita Dhamayanti, Prof, MD, PhD
	Retno Pudjiati, PhD
	Lathifah Hanum
	Affan Naufal
	Susan Sawyer, Prof, MD, PhD

Powered by Editorial Manager® and ProduXion Manager® from Aries Systems Corporation

Additional Information:	
**Question**	**Response**
**Financial Disclosure**<br><br>Enter a financial disclosure statement that describes the sources of funding for the work included in this submission. Review the submission guidelines for detailed requirements. View published research articles from *PLOS ONE* for specific examples.<br><br>This statement is required for submission and **will appear in the published article** if the submission is accepted. Please make sure it is accurate.<br><br>**Funded studies**<br>Enter a statement with the following details:<br>• Initials of the authors who received each award<br>• Grant numbers awarded to each author<br>• The full name of each funder<br>• URL of each funder website<br>• Did the sponsors or funders play any role in the study design, data collection and analysis, decision to publish, or preparation of the manuscript?<br><br>Did you receive funding for this work?	Yes
Please add funding details.<br>as follow-up to "**Financial Disclosure**<br><br>Enter a financial disclosure statement that describes the sources of funding for the work included in this submission. Review the submission guidelines for detailed requirements. View published research articles from *PLOS ONE* for specific examples.<br><br>This statement is required for submission and **will appear in the published article** if the submission is accepted. Please make sure it is accurate.	This research was supported by Universitas Indonesia (Grant number NKB-NKB-1417/UN2.RST/HKP.05.00/2022)

**Funded studies**<br>Enter a statement with the following details:<br>• Initials of the authors who received each award<br>• Grant numbers awarded to each author<br>• The full name of each funder<br>• URL of each funder website<br>• Did the sponsors or funders play any role in the study design, data collection and analysis, decision to publish, or preparation of the manuscript?<br><br>Did you receive funding for this work?"	
Please select the country of your main research funder (please select carefully as in some cases this is used in fee calculation).<br>as follow-up to "**Financial Disclosure**<br><br>Enter a financial disclosure statement that describes the sources of funding for the work included in this submission. Review the submission guidelines for detailed requirements. View published research articles from *PLOS ONE* for specific examples.<br><br>This statement is required for submission and **will appear in the published article** if the submission is accepted. Please make sure it is accurate.<br><br>**Funded studies**<br>Enter a statement with the following details:<br>• Initials of the authors who received each award<br>• Grant numbers awarded to each author<br>• The full name of each funder<br>• URL of each funder website<br>• Did the sponsors or funders play any role in the study design, data collection and analysis, decision to publish, or preparation of the manuscript?<br><br>Did you receive funding for this work?"	INDONESIA - ID
**Competing Interests**	The authors have declared that no competing interests exist

Use the instructions below to enter a competing interest statement for this submission. On behalf of all authors, disclose any competing interests that could be perceived to bias this work—acknowledging all financial support and any other relevant financial or non-financial competing interests.<br><br>This statement is required for submission and **will appear in the published article** if the submission is accepted. Please make sure it is accurate and that any funding sources listed in your Funding Information later in the submission form are also declared in your Financial Disclosure statement.<br><br>View published research articles from *PLOS ONE* for specific examples.<br><br>**NO authors have competing interests**<br><br>Enter: *The authors have declared that no competing interests exist.*<br><br>**Authors with competing interests**<br><br>Enter competing interest details beginning with this statement:<br><br>*I have read the journal's policy and the authors of this manuscript have the following competing interests: [insert competing interests here]*<br><br>* typeset	
**Ethics Statement**<br><br>Enter an ethics statement for this submission. This statement is required if the study involved:<br><br>• Human participants<br>• Human specimens or tissue<br>• Vertebrate animals or cephalopods<br>• Vertebrate embryos or tissues	This study was conducted according to the guideline laid down in the Declaration of Helsinki and all procedures involving research study participants were approved by the Ethic Committee of Fakultas Kedokteran Universitas Indonesia, with approval number 829a/UN2.F1/ETIK/PPM.00.02/2021. Written consent was obtained from all subjects.

Powered by Editorial Manager® and ProduXion Manager® from Aries Systems Corporation

• Field research<br><br>Write "N/A" if the submission does not require an ethics statement.<br><br>General guidance is provided below. Consult the submission guidelines for detailed instructions. **Make sure that all information entered here is included in the Methods section of the manuscript.**	

Powered by Editorial Manager® and ProduXion Manager® from Aries Systems Corporation

**Format for specific study types**<br><br>**Human Subject Research (involving human participants and/or tissue)**<br>• Give the name of the institutional review board or ethics committee that approved the study<br>• Include the approval number and/or a statement indicating approval of this research<br>• Indicate the form of consent obtained (written/oral) or the reason that consent was not obtained (e.g. the data were analyzed anonymously)<br><br>**Animal Research (involving vertebrate animals, embryos or tissues)**<br>• Provide the name of the Institutional Animal Care and Use Committee (IACUC) or other relevant ethics board that reviewed the study protocol, and indicate whether they approved this research or granted a formal waiver of ethical approval<br>• Include an approval number if one was obtained<br>• If the study involved *non-human primates*, add *additional details* about animal welfare and steps taken to ameliorate suffering<br>• If anesthesia, euthanasia, or any kind of animal sacrifice is part of the study, include briefly which substances and/or methods were applied<br><br>**Field Research**<br><br>Include the following details if this study involves the collection of plant, animal, or other materials from a natural setting:<br>• Field permit number<br>• Name of the institution or relevant body that granted permission	
**Data Availability**<br><br>Authors are required to make all data underlying the findings described fully available, without restriction, and from the time of publication. PLOS allows rare exceptions to address legal and ethical concerns. See the PLOS Data Policy and FAQ for detailed information.	Yes - all data are fully available without restriction

A Data Availability Statement describing where the data can be found is required at submission. Your answers to this question constitute the Data Availability Statement and **will be published in the article**, if accepted.<br><br>**Important:** Stating 'data available on request from the author' is not sufficient. If your data are only available upon request, select 'No' for the first question and explain your exceptional situation in the text box.<br><br>Do the authors confirm that all data underlying the findings described in their manuscript are fully available without restriction?	
**Describe where the data may be found in full sentences. If you are copying our sample text, replace any instances of XXX with the appropriate details.**<br><br>• If the data are **held or will be held in a public repository**, include URLs, accession numbers or DOIs. If this information will only be available after acceptance, indicate this by ticking the box below. For example: *All XXX files are available from the XXX database (accession number(s) XXX, XXX.).*<br>• If the data are all contained **within the manuscript and/or Supporting Information files**, enter the following: *All relevant data are within the manuscript and its Supporting Information files.*<br>• If neither of these applies but you are able to provide **details of access elsewhere**, with or without limitations, please do so. For example:<br><br>*Data cannot be shared publicly because of [XXX]. Data are available from the XXX Institutional Data Access / Ethics Committee (contact via XXX) for researchers who meet the criteria for access to confidential data.*<br><br>*The data underlying the results presented in the study are available from (include the name of the third party*	All relevant data are within the manuscript

and contact information or URL).<br>• This text is appropriate if the data are owned by a third party and authors do not have permission to share the data.<br><br>* typeset	
Additional data availability information:	

Powered by Editorial Manager® and ProduXion Manager® from Aries Systems Corporation

Manuscript

Click here to access/download;Manuscript;Effectiveness paper_1.0 plos one.docx

Adolescent weight management counseling: the effectiveness of an online training program for primary healthcare professionals in Indonesia

Fransisca Handy Agung,[a,] Rini Sekartini,[b] Nani Sudarsono,[c] Aryono Hendarto,[b] Retno Asti Werdhani,[c] Meita Dhamayanti,[d] Retno Pudjiati,[e] Lathifah Hanum,[e] Affan Naufal,[f] Susan M Sawyer [g]

Affiliation

[a] Faculty of Medicine, Universitas Pelita Harapan

Jl. Jend. Sudirman No.20, Bencongan, Kelapa Dua, Tangerang, Banten, Indonesia 15810

[b] Department of Child Health, Faculty of Medicine Universitas Indonesia

Jl. Salemba Raya No.6 Jakarta Pusat, Jakarta, Indonesia 10430

[c] Department of Community Medicine, Faculty of Medicine Universitas Indonesia

Jl. Salemba Raya No.6 Jakarta Pusat, Jakarta, Indonesia 10430

[d] Department of Child Health, Faculty of Medicine Universitas Padjajaran,

Jl. Professor Eyckman No.38, Pasteur, Bandung, West Java, Indonesia

[e] Faculty of Psychology Universitas Indonesia,

Kampus UI, Depok, West Java, Indonesia

[f] Balaraja District Hospital

Jl. Rumah Sakit No. 88, Tangerang, Banten, Indonesia

[g] Centre for Adolescent Health, Royal Children’s Hospital; Murdoch Children’s Research

Institute; and Department of Paediatrics, University of Melbourne, 50 Flemington Road, Parkville VIC 3052, Australia

Email address and ORCID ID

Fransisca Handy Agung: Fransisca.agung@uph.edu ORCID ID 0000-0003-3516-6089

Rini Sekartini rsekartini@yahoo.com ORCID ID 0000-0001-9859-5468

Nani Cahyani Sudarsono nani.cahyani@ui.ac.id ORCID ID 0000-0001-6856-0694

Aryono Hendarto aryhendarto@yahoo.com ORCID ID 0000-0002-7714-8332

Retno Asti Werdhani retnoasti@yahoo.com ORCID ID 0000-0002-3280-4295

Retno Pudjiati, retno_pudjiati@yahoo.com ORCID 0000-0001-5231-7139

Meita Dhamayanti meita_d@yahoo.com ORCID ID 0000-0001-9661-0708

Lathifah Hanum, lathifah.hanum08@ui.ac.id ORCID ID 0000-0003-1771-335X

Affan Naufal, affanaufal@gmail.com ORCID ID 0000-0001-5701-9026

Susan Sawyer: susan.sawyer@rch.org.au ORCID ID 0000-0002-9095-358X

Corresponding author:

Fransisca Handy Agung

Fransisca.agung@uph.edu

Faculty of Medicine Universitas Pelita Harapan

Jl. Jendral Sudirman No 20, Bencongan, Tangerang, Indonesia 15810

ABSTRACT

Background: Overweight and obesity are growing public health concerns globally for which innovative prevention and care delivery efforts are required. We recently developed a web-based training program to improve the quality of health professionals' weight management counseling of adolescents in Indonesia. Having previously confirmed its acceptability, this study aimed to measure the effectiveness of the program through a randomized controlled trial.

Methods: We recruited 64 primary healthcare professionals from 17 provinces across Indonesia who were randomized to participate in a 4-week online training (intervention group [IG, n=32] or a waitlist control group [CG, n=32]. Using active learning approaches, the training program focused on adolescent development, psychosocial assessment, motivational interviewing (MI), and parent engagement. Participants in each arm were asked to record two counseling sessions with adolescents. These were objectively rated by trained psychologists using a validated tool, and also by qualitative assessments of counseling quality. In both groups, the first recorded counseling session occurred before the training. The second recording occurred after the training for IG participants, but not for CG participants.

Results: IG participants demonstrated significant improvements in knowledge and counseling skills ($p<0.001$, t-test). This included improvements in introductory remarks, quality of psychosocial assessment, and MI skills. There was no change in the extent of parental involvement. The MI training successfully oriented the counseling sessions towards a more collaborative and participatory conversation for supporting behavioral change.

Conclusion: This novel online training program improved the knowledge and counseling skills of Indonesian primary healthcare professionals. Greater emphasis on engaging parents and more guidance on conducting telehealth counseling may improve parental involvement in future iterations.

Keywords: adolescents, behavior change, counseling skills, healthy lifestyle, internet-based training, obesity, weight management

INTRODUCTION

Globally, the prevalence of obesity in children and adolescents has tripled in the last four decades. [1] Low- and middle-income countries have experienced a very rapid recent acceleration of obesity in children and adolescents. [2] This is exemplified by Indonesia, a country of 250 million people and over 35 million adolescents aged 10-18 years,[3] whose prevalence of overweight or obesity has increased ten times in the past decade,[4] even before the anticipated escalation following the COVID-19 pandemic.[5]

Overweight at a young age carries a high risk of living with overweight or obesity in adulthood [6], and brings forward the timing of associated ‘adult’ conditions such as cardiovascular disease and diabetes. [7, 8] The prevalence of these non-communicable diseases has also been increasing in adolescent populations.[9] This is particularly concerning given that already in Indonesia, cardiovascular-metabolic diseases such as stroke, heart disease, and diabetes are the leading causes of mortality and morbidity.[10].

Adolescence is a critical period for managing obesity due to the dynamic physiological and psychological changes inherent to this period of growth and development.[11, 12] While behavior change is central to clinical approaches to managing obesity at any age,[11, 13] adolescence heralds growing individual autonomy and capacity to regulate health-related behaviors, such as food choices and physical activity.[13] However, behavior change interventions to address behavior-related health problems are underutilized in healthcare settings and health professionals are poorly prepared to provide adequate counseling to adults over their healthiest weight, [7, 9] let alone adolescents.

Motivational Interviewing (MI) is a clinical approach for behavior change counseling [14, 15]. It is the most widely used counseling technique in the field of weight management, including for adolescents in primary care.[16-18]. MI is a patient-centered counseling technique for exploring patient motivation and strengths for change. It is a collaborative approach that aims

to help patients set and reach their own goals. [15, 19]. To increase the likelihood of behavior change, it is also important to consider the social and physical environments, including the availability and accessibility of resources that support more healthy lifestyles.[20] Further, as parents are critical influencers of social and physical home environments for adolescents, [21, 22] their involvement in adolescent weight management is widely recommended.[23]

In Indonesia, there is no specific teaching on behavioral counseling in medical [24] and nursing schools [25]. Furthermore, there is an absence of continuing medical education programs to help primary healthcare providers develop the necessary attitudes, knowledge and skills to address behavior change in adolescents who are over their healthiest weight. To provide accessible training across a huge archipelago like Indonesia, and in the context of COVID-19, we recently developed an internet-based training program to promote behavior change counseling for weight management in adolescents. Previously, the content and mode of delivery of the training pilot was rated highly by participants.[26] The aim of the current paper is to assess the effectiveness of this training program through a randomized controlled trial of primary healthcare professionals to understand its feasibility and identify areas of refinement.

METHOD

The study design was a two-arm randomized controlled trial that recruited 64 health professionals from primary healthcare centers in 17 of Indonesia’s 34 provinces. Following recruitment to the study, participants (primary healthcare doctors and nurses) were randomized to receive the training (intervention group [IG] or to a waitlist control group [CG]) based on their district. At baseline and post-intervention, a pre- and post-training knowledge test was administered and two audio-recorded counseling sessions with adolescents with overweight or obesity and their parents were submitted by participants for assessment. The primary outcome of the training was change in health professional knowledge of behavior change counseling,

while the secondary outcome was change in counseling skills, using objective ratings of each audio-recording that was made by trained professional observers (clinical psychologists) using a specially designed assessment measure. Following submission of their pre-training audio-recording, the IG participated in the 4-week training program, and then audio-recorded a second counseling session with the same two adolescents. The CG participants submitted their second audio-recording at the same time as the IG, prior to them undertaking the training (see Figure 1).

Recruitment of Study Participant (Health Professionals) and Adolescents

The sample size was determined using the mean-difference formula[27] from an RCT on motivational interviewing for obesity.[28] Based on this calculation, 60 adolescents were required for each group to assess counselling effectiveness.[28] We undertook a brief survey of health professionals who considered that they could recruit up to two adolescents each, suggesting that we needed at least 30 health professionals for each group (intervention group [IG], n=32) and waitlist control group [CG], n=32). Healthcare professionals who had routinely provided adolescent health services at a primary health care center for at least 6 months were recruited in February 2022. Participants were the person in-charge of~~for~~ adolescent friendly health service (AFHS) at the primary health care centers nominated by their provincial health offices. These were mostly midwives, nurses and general practitioners. Of these, we selected only the nurses and general practitioners as adolescent nutrition was one of their competencies, but not for midwives. These participants completed a baseline survey about their sociodemographic characteristics, personal nutritional status and eating habits, level of physical activities and their own parenting style (for those who were parents). Each participant was then required to identify two adolescents with BMI > 85th percentile (WHO, 2007). Participants were asked to explain the study to each adolescent and their parent/s and gain their verbal consent to participate in the study. Prior to the audio-recording of counseling sessions,

written informed consent was obtained from both adolescents and parent/s. All resources were available through a website that was specially developed for the study (ramahremaja.id®) which was also used to upload materials (i.e., signed consent forms) and assessments.

The training program

The training program was internet-based and consisted of synchronous and non-synchronous sessions (blended learning) that were completed over four weeks across March to April 2022. Following a needs assessment,[29] the training program was developed using constructive alignment theory to ensure the alignment between the intended learning outcomes, learning activities and assessment tasks.[30] It consisted of four main topics: adolescent growth and development, parenting adolescents, healthy eating and physical activity, and behavioral change counseling techniques for adolescent patients using MI approaches. Each topic consisted of individual learning tasks and an online meeting with all participants. Active learning principles underpinned the development of all learning activities, which utilized multifaceted materials including explanatory videos, audio recordings of exemplar consultations with adolescents, quizzes, virtual interactive case vignettes, and written assignments (e.g., develop a healthy meal plan, develop a schedule for physical activity, map local healthy lifestyle resources, develop a personal behavioral change plan). The agenda for each weekly online meeting consisted of group work, a group presentation and an interactive discussion lead by the facilitator. In the last online meeting, participants engaged in a formal role-play with adolescents in a small group setting. Constructive feedback was provided to each participant, with each member of the group learning from feedback to others.

Knowledge Assessment

The primary outcome was improved health professional knowledge of behavior-change counseling for weight management of adolescents. This was assessed using a 47-item questionnaire that was specifically developed for the study that consisted of questions

exploring knowledge of healthy eating, physical activity, screen time, basic communication skills with adolescent patients, psychosocial screening, motivational interviewing techniques and parent roles in adolescent behavior change. Content validity was determined qualitatively by nine experts (clinicians, academics, members of relevant professional organizations and representatives of the Ministry of Health) to ensure that the content was consistent with current national guidelines. Following this, face validity was undertaken by 11 Indonesian AFHS national trainers. Item analysis was done by calculating the Item Difficulty Index (IDI) using the average score of each item and Discrimination Index (DI) using Point of Biserial coefficient correlation in a sample of 67 health workers from 30 out of 34 provinces in Indonesia.[31] The item analysis procedure was followed by consideration of the learning objectives and materials and led to revision of some items. The final knowledge assessment tool had a Cronbach-α 0.786, indicating good internal consistency.

Counseling Skill Assessment

The secondary outcome was improved counseling skills for weight management of adolescent patients. Assessment of counseling skills was evaluated both quantitatively and qualitatively by trained professional raters, as well as by participant self-assessment.

Quantitative assessment

Assessment tools were specially developed for this study, based on the content of the training module. With the plan of using trained professional raters (psychologists), the assessment tool was modified from the Behavioral Change Counseling Index (BECCI), a commonly used tool for evaluating behavioral-change counseling quality in healthcare settings.[32, 33] Three further items were added which were specific for counseling sessions with adolescent patients, namely: greeting adolescents and their parents; inviting adolescents to talk on their own without their parents (including a confidentiality statement);[34] and, discussing the approach to parental involvement with the parents. The scoring for each item in the assessment tool was

also modified from the original BECCI. Instead of a 5-point Likert scale, our assessment tool used a 4-point Likert scale with slightly modified explanations of each item with the intention of improving its objectivity.[35, 36] A rating of 0 was the lowest (no statements heard) with 3 being the highest (all required statements heard). The assessment tool was called the Adolescent Behavioral Change-Counseling Assessment Tool (ABC-CAT).[37]

The ABC-CAT underwent a validation process. Firstly, content validity was undertaken by nine experts, followed by assessment of face validity by ten clinical psychologists. Construct validity was undertaken with corrected correlation coefficient analysis (n=125) using the first counseling session review from both groups (IG and CG). The inter-item corrected value was more than 0.3 for all items and Cronbach-α 0.839, which revealed good validity and good internal consistency.[37]

The final ABC-CAT[37] assessed four domains: 1) Introduction (a greeting and how to start the counseling session); 2) Psychosocial screening (this commenced by inviting the adolescent to talk without their parents, included a confidentiality statement prior to the psychosocial screening which focused on evaluating the social determinants of healthy weight); 3) MI basic communication skills and MI stages (engaging, focusing, evoking and planning); and 4) Parental involvement (this evaluated whether the participants discussed any of two parenting roles, namely around structuring and supporting behavior change in adolescents).

Eight trained clinical psychologists assessed the audio-taped counseling sessions. These psychologists underwent training for the assessment, after which they each scored 15 audio recordings from an earlier pilot for the study. The inter-rater reliability score (the inter-class correlation coefficient test using the simplified Winer and Walter formula) was 0.942 (CI 0.868 – 0.973), which showed strong agreement.[38]

Each participant uploaded two sets of two recorded counseling sessions onto the website, four weeks apart. IG participants uploaded an audio-recording of the same two adolescents before

and after the training. The CG participants also uploaded two audio-recordings on two occasions of the same two adolescents, but both were prior to training. Each recording was coded and assigned to a rater by an embedded system in the website. The system ensured that each of the four recordings submitted by each participant was reviewed by different raters, who reviewed them blindly.

Qualitative assessment

Qualitative assessment is a flexible, holistic and nonstatistical assessment method where participants can process what they learned from the experience by provision of feedback soon after the counseling session.[39, 40] For each audio-recording, the trained rater was asked to write a narrative description about the strengths and weaknesses of each counseling session and to provide an overall assessment of its quality. This narrative description was made available to each participant, confidentially, through their training portal in the website.

Data Analysis

A summary of baseline data of the participants was developed to show the baseline similarities of each group. To evaluate the effectiveness of the training, we compared changes in knowledge and skills between each group. Differences between pre- and post-intervention knowledge scores were assessed by group. Change in skills was assessed quantitatively using differences in the ABC-CAT assessment questionnaire and also qualitatively. Continuous variables were described by standard descriptive statistics containing mean and standard deviation. Comparison between groups was done using the unpaired t-test.

RESULTS

Sample characteristics

A CONSORT diagram that details recruitment and retention is shown in Figure 2. A total of 64 health professionals (52 females) were recruited, which comprised 35 nurses and 29 physicians. Fourteen (22%) reported some prior training in adolescent friendly health services

(AFHS). The adolescent patients who consented to be audiotaped were similar in age and sex by group. Each adolescent was required to be accompanied by one of their parents. The parents were predominantly mothers, although there were 17 fathers in each group. As show in Table 1, around two-thirds of health professionals and parents themselves experienced overweight or obesity. The predominant weight category for adolescents was obesity rather than overweight (IG, 92%: CG, 91%).

In both adult populations (health professionals and parents) a proportion of participants had unhealthy eating habits (low fruit consumption, deep fried food, sugary beverages, ultra-processed food and junk meal). Mostly, this was eating vegetables less frequently than recommended and reporting unhealthy snacking. A higher proportion of adolescents than parents reported low consumption of vegetables and high levels of unhealthy snacking. Adolescents also commonly reported a variety of other unhealthy eating behaviors. More than half of the adolescents had low levels of physical activity. For each of these lifestyle characteristics, there were no significant differences between adolescents by group (Table 1).

Knowledge Assessment

At baseline, there were no differences in knowledge scores ($p = 0.902$, unpaired t-test) between IG and CG participants. Four weeks later, the IG had significantly higher scores ($p < 0.001$, unpaired t-test) than the CG (Table 2).

Counseling skill assessment

Quantitative assessment (ABC-CAT)

As part of training and assessment, participants engaged in a counseling session with adolescents, which they audio-recorded and uploaded to the study website. Due to the COVID-19 pandemic, participants were free to conduct counseling sessions remotely by video (e.g., zoom, telephone) or face-to-face at the clinic. A total of 256 sessions were uploaded, 128 sessions from each group. Five of 64 adolescents did not return for the 2nd counselling session

(IG, n=1; CG, n=4); two did not respond to follow-up texts and phone calls, two had moved to another city, and one was hospitalized due to a traffic injury. Instead, audio recordings were made with other adolescents who met the study criteria. A total of 116 (45%) sessions were undertaken virtually, which were similarly distributed by group (IG 47%, CG 44%). At baseline, there were no differences in assessment by group (Table 3). Assessment of the 2nd audio-recording revealed significant differences ($p < 0.001$, unpaired t-test) in the counselling quality of participants in the IG compared to the CG, as measured by the ABC-CAT domains. Significant differences were seen in relation to both aspects of psychosocial screening, namely the provision of a confidentiality assessment and psychosocial screening itself. There was a highly significant difference between the two groups for all but one assessment item for MI (the elicit-provide-elicit skill just failed to reach significance, $p=0.061$). There were no differences between groups in the use of introductory greetings ($p=0.609$) or in the extent of parental involvement by group ($p= 0.455$).

Qualitative Assessment

At baseline, the narrative assessment of communication techniques by qualified psychologists (who were blinded to both the timing [pre/post] and group) suggested there were substantial opportunities for improvement by participants in both groups. The consultations were commonly stilted, with little evidence of a two-way conversation that actively engaged adolescents in the discussion, let alone showed evidence of goal setting or planning for change.

The counseling session felt interrogative, so the conversation went one way and tended to be monotonous. It would be better if the adolescent was more actively involved in determining their own changes in eating patterns and activities using the MI technique (Counseling 1, CG, Participant 25, Psychologist G).

In some sections of the counseling session, the health worker sounded confused about what else to ask the adolescent. To minimize their confusion, the health worker could reflect more

on the adolescent's statements and use this to lead to behavioral changes (Counseling 1, IG, Participant 5, Psychologist E).

After the training, assessor comments suggest there were differences between participants by group. IG participants used more communication techniques, especially more open questions and reflective listening. There was also good appreciation of the different MI stages, with the exception of evoking skills.

It was apparent that the adolescent patient was fully involved in a two-way discussion regarding changes in her behavior, especially in developing diet and activity plans that the adolescent could do on her own (Counseling 2, IG, Participant 4, Psychologist D).

[The participant] conducted an individual session with the adolescent, ensured medical confidentiality and asked open-ended questions that made the adolescent feel comfortable. The health worker applied MI techniques, especially around the engaging process, and involved the adolescent in a two-way discussion, evoking using scaling method and inviting the adolescent to plan for change, again using a two-way conversation (Counseling 2, IG, Participant 31, Psychologist A).

The preparatory steps required for a psychosocial assessment, such as providing the opportunity for adolescents to talk with the health professional alone and making a confidentiality statement, were less well done in comparison to the behavioral change counseling steps. Further, there was no significant difference ($p = 0.455$, unpaired t-test) in the involvement of parents by group. IG participants provided greater opportunities for adolescents to share their stories and to develop two-way participatory conversations in relation to developing a behavior-change plan.

DISCUSSION

To our knowledge, this is the first online training program that has been developed to enhance counseling skills for primary health professionals caring for adolescents with overweight and obesity in Indonesia, a highly populous low-income country from which health professionals could greatly benefit from effective training interventions. Using a randomized controlled trial that was supported by an online web platform, we demonstrated objective improvements in health professional knowledge of counseling approaches, the primary outcome of the study. Importantly, we also showed significant improvements in counselling skills, the secondary outcome, as shown by improved quality of audiotaped counseling sessions with adolescents. We have previously shown excellent acceptability of the training, with participants rating it highly for both the clarity of content and the enjoyment of training methods (manuscript under review, results not presented here).

This training was ambitious. In terms of content, up to four new concepts were introduced to participants across the four-week program. These related to normal adolescent growth and development and how patterns of overweight and obesity differ to this; approaches to engaging adolescents, communicating with adolescents and parents, and taking a confidential psychosocial history; normal patterns of adolescent nutrition and physical activity, consideration of meal plans for parents, and assisting families to map local resources; and motivational interviewing to address motivation, physical and social environments influencing adolescent lifestyle.[20, 29] While we intentionally recruited participants from AFHS in Indonesia with the expectation that they would have reasonable knowledge of at least the first two concepts, only a fifth of participants reported some form of training in adolescent health. It is interesting to reflect on how the results might have differed had the participants started with higher baseline knowledge and skills, such as understanding how to involve parents within consultations. Beyond complex content, novel approaches to delivery were also utilized. Firstly, this related to the nature of blended learning within the online training program which

included synchronous and asynchronous methods. In addition, as the training was conducted during the COVID-19 pandemic, around 45% of counseling session were done remotely, which many participants (and families) had little familiarity with. Notwithstanding the complexity of concepts and context, the extent of active learning approaches designed to enhance knowledge and skills is likely to have contributed to the positive findings from this study.

Despite recognition of the particular value of e-learning and blended learning for health professionals in LMIC, the lack of high quality studies from LMIC is also well recognized. In 2015, a systematic review by Al-Shorbaji et al. that examined global e-learning and blended learning methods and their effect on knowledge, skills, attitudes and satisfaction found only five studies had been conducted in LMIC.[41] Disappointingly, a more recent systematic evaluation of e-learning for medical education in LMIC from 2020 found that poor quality studies predominated[42]. For example, of 52 reviewed studies, only four (8%) were randomized controlled trials, and only ten (19%) studies assessed change in skills.

Notwithstanding the novelty of content and the complexity of the setting in the peak of the COVID-19 pandemic, there were significant improvements in intervention participants' knowledge and skills, including MI techniques. These findings are consistent with a study by Antognoli et al. who emphasized that clinician confidence in counseling is enhanced by building skills with the opportunity to practice, engage patients, and receive feedback to hone counseling skills,[43] as we also attempted.

We failed to show better parental involvement in developing the behavioral-change plan for their children. Beyond focusing on the individual with overweight, providing parents with the necessary understanding, skills and resources to model more healthy lifestyles for the whole family is a central part of adolescent weight management practices.[20, 29] We do not know the extent to which insignificant differences around engaging parents reflected insufficient focus on parenting within the training program, or whether other factors were responsible, such

as parent and professional lack of familiarity with online consultations which may have resulted in reduced engagement of parents during consultations.

As shown in other online training, [44] allowing time for participants to flexibly engage with the learning material is arguably a strength of this training. We also believe that the synchronous weekly meetings which provided a fun, interactive learning environment enabled participants to engage with the facilitators and other participants from across Indonesia, which is likely to have enhanced participant motivation and improved the effectiveness of the training.[45, 46] Health professionals with favorable personal health behaviors are more likely to counsel patient about healthy lifestyles than those with less favorable lifestyle habits and perceived low self-efficacy in nutrition care.[47] The lifestyle characteristics of the health professionals were representative of the Indonesian National Health Survey data on eating behavior and physical activity of the Indonesian population.[4] We hypothesize that the focus on health professionals in relation to their own lifestyles (a hands-on exercise was to develop a personal plan for a meal, physical activity and personal behavior change) may have promoted their self-efficacy in conducting a behavioral change counseling session for adolescents.

Consistent with a systematic review of online learning in LMIC,[42] a major challenge for some participants was poor internet connectivity, as well as low internet literacy in a minority of participants. Following our initial pilot study, efforts were made to reduce the bandwidth required, but other approaches such as gathering participants in a place with good internet connectivity to download the training material and upload assessment tasks may also be required. Offering the interactive skill building components of the four-week program in a more intensive manner, face-to-face, may also obviate this.

A strength of this study is the care that went into the development of the training program. A needs assessment[29] involving adolescent and their parents from different socio-demographic background was conducted to generate learning objectives and inform the training materials.

Using constructive alignment theory [30], careful attention was made to develop the approach to assessment. A nation-wide study informed the development of the knowledge assessment tool and the ABC-CAT was carefully developed and validated as a measure of skill assessment.[37] Objective rating by multiple trained raters of each counseling session was undertaken blindly, managed through the study website. There are also a number of limitations. The study was undertaken during the COVID-19 pandemic, with many health professionals, patients and parents alike being novice users of online consultations. Given the importance of parents in providing the social and physical environments for healthy lifestyle development at home,[20, 29] the lack of improvement in parent engagement within the counseling sessions suggests further study is required to understand whether this reflected the challenges of the pandemic for parents or perhaps a lack of emphasis within the training program. The many concepts and materials that were learnt in a short period raises questions about whether these practices are able to be embedded within daily consultations, something that also warrants further study. Developing a community of practice is one potential strategy to maintain the group's motivation and provide a space to 'trouble shoot' when participants face roadblocks.[48]

CONCLUSION

This carefully designed online training program shows promise in increasing the quality of primary care professionals' weight management counseling skills for adolescents. Future research is required to determine the impact of improved counseling skills on the actual behaviors of adolescents and their parents. However, this training program, and the website that it is based on, shows potential as a training strategy that warrants further exploration in Indonesia, given the extent of training needs in this highly populous country.

Acknowledgements

The authors thank: Aryo Sudiro from St. Patrick, the e-learning consultant who developed the website; the Ministry of Health of the Republic of Indonesia, especially the director and the staff of The Directorate of Nutrition, Child and Maternal Health; and the provincial health offices from the 17 provinces who assisted with the nationwide pilot. The authors also thank: the Indonesia national AFHS facilitators (Indira Dewi, Rinalco Franky, Ni Made Jendri, Ninin Anggreani, Dian Sharie, Dewi Nur, Risna Yanti, lka Maharani, lndah Ariestanti, Dena Restiana, Asep Zaenal); the clinical psychologist team (Dian Oriza, Annisa Rahmalia, Utari Krisnamurthi, Sri Wulandari, Fina Dwi Putri, Widya Gunawan, Diajeng Tri, Fadhilah Erynanda, Risky Adinda); and Tan Shot Yen, who significantly contributed to the preparation phase of the training material, during and after the training pilot. Lastly, the authors thank all of the nurses and physicians, adolescents and parents who participated in the pilot intervention

REFERENCES

1. World Health Organization. World Health Organization obesity and overweight fact sheet. 2016.
2. Dong Y, Jan C, Ma Y, Dong B, Zou Z, Yang Y, et al. Economic development and the nutritional status of Chinese school-aged children and adolescents from 1995 to 2014: an analysis of five successive national surveys. Lancet Diabetes Endocrinol. 2019;7(4):288-99.
3. Central Bureau of Statistics. Profil anak indonesia 2018. Jakarta: The Ministry of Women Empowerment and Child Protection (MoWEC). 2018.
4. The Indonesian Institute of Health Research and Development. Basic Health Survey 2018. Jakarta: Publishing Body of The Indonesian Institute of Health Research and Development. 2018
5. Stavridou A, Kapsali E, Panagouli E, Thirios A, Polychronis K, Bacopoulou F, et al. Obesity in Children and Adolescents during COVID-19 Pandemic. Children (Basel). 2021;8(2):135.
6. Singh AS, Mulder C, Twisk JW, Van Mechelen W, Chinapaw MJ. Tracking of childhood overweight into adulthood: a systematic review of the literature. Obes Rev. 2008;9(5):474-88.
7. Sommer A, Twig G. The impact of childhood and adolescent obesity on cardiovascular risk in adulthood: a systematic review. Curr Diab Rep. 2018;18:1-6.
8. Weihrauch-Blüher S, Schwarz P, Klusmann J-H. Childhood obesity: increased risk for cardiometabolic disease and cancer in adulthood. Metabolism. 2019;92:147-52.
9. Bendor CD, Bardugo A, Pinhas-Hamiel O, Afek A, Twig G. Cardiovascular morbidity, diabetes and cancer risk among children and adolescents with severe obesity. Cardiovasc Diabetol. 2020;19(1):1-14.

10. Marthias T, Anindya K, Ng N, McPake B, Atun R, Arfyanto H, et al. Impact of non-communicable disease multimorbidity on health service use, catastrophic health expenditure and productivity loss in Indonesia: a population-based panel data analysis study. BMJ Open. 2021;11(2):e041870.
11. Cardel MI, Jastreboff AM, Kelly AS. Treatment of adolescent obesity in 2020. JAMA. 2019;322(17):1707-8.
12. Norris SA, Frongillo EA, Black MM, Dong Y, Fall C, Lampl M, et al. Nutrition in adolescent growth and development. Lancet. 2021.
13. Teixeira PJ, Marques MM. Health behavior change for obesity management. Obes Facts. 2017;10(6):666-73.
14. Resnicow K, McMaster F, Bocian A, Harris D, Zhou Y, Snetselaar L, et al. Motivational interviewing and dietary counseling for obesity in primary care: an RCT. Pediatrics. 2015;135(4):649-57.
15. Rollnick S, Miller WR, Butler C. Motivational interviewing in health care: helping patients change behavior: Guilford press; 2008.
16. Barlow SE. Expert committee recommendations regarding the prevention, assessment, and treatment of child and adolescent overweight and obesity: summary report. Pediatrics. 2007;120(Supplement 4):S164-S92.
17. World Health Organization. Technical package for cardiovascular disease management in primary health care: healthy-lifestyle counselling. World Health Organization; 2018.
18. Vallabhan MK, Jimenez EY, Nash JL, Gonzales-Pacheco D, Coakley KE, Noe SR, et al. Motivational Interviewing to Treat Adolescents With Obesity: A Meta-analysis. Pediatrics. 2018;142(5).
19. Resnicow K, Davis R, Rollnick S. Motivational interviewing for pediatric obesity: conceptual issues and evidence review. J Am Diet Assoc. 2006;106(12):2024-33.
20. Neufeld LM, Andrade EB, Ballonoff Suleiman A, Barker M, Beal T, Blum LS, et al. Food choice in transition: adolescent autonomy, agency, and the food environment. Lancet. 2022;399(10320):185-97.
21. Patton GC, Sawyer SM, Santelli JS, Ross DA, Afifi R, Allen NB, et al. Our future: a Lancet commission on adolescent health and wellbeing. Lancet. 2016;387(10036):2423-78.
22. Wang J, Fielding-Singh P. How food rules at home influence independent adolescent food choices. J Adolesc Health. 2018;63(2):219-26.
23. Pollak KI, Coffman CJ, Tulsky JA, Alexander SC, Østbye T, Farrell D, et al. Teaching physicians motivational interviewing for discussing weight with overweight adolescents. J Adolesc Health. 2016;59(1):96-103.
24. Konsil Kedokteran Indonesia. Standar kompetensi dokter. Jakarta: Konsil Kedokteran Indonesia. 2006.
25. Persatuan Perawat Nasional Indonesia. Standar kompetensi perawat Indonesia. Bidang Organisasi PP-PPNI: Jakarta. 2005.
26. Agung FH S, b R, Sudarsono N, Hendarto A, Werdhani RA, Dhamayanti M, Pudjiati,e R, Hanum, L, Naufal A, Sawyer S. Development of an internet-based intervention to improve health professionals' counseling skills around adolescent weight management in Indonesia. Plos One. 2023;(Under Review).
27. Charan J, Biswas T. How to calculate sample size for different study designs in medical research? Indian journal of psychological medicine. 2013;35(2):121-6.
28. Pakpour AH, Gellert P, Dombrowski SU, Fridlund B. Motivational interviewing with parents for obesity: an RCT. Pediatrics. 2015;135(3):e644-52.
29. Agung FH, Sekartini R, Sudarsono NC, Hendarto A, Dhamayanti M, Werdhani RA, et al. The barriers of home environments for obesity prevention in Indonesian adolescents. BMC Public Health. 2022;22(1):1-10.
30. Biggs J, Tang C. Teaching for quality learning at university: McGraw-hill education (UK); 2011.
31. Quaigrain K, Arhin AK. Using reliability and item analysis to evaluate a teacher-developed test in educational measurement and evaluation. Cogent Education. 2017;4(1):1301013.

32. Lundahl B, Droubay BA, Burke B, Butters RP, Nelford K, Hardy C, et al. Motivational interviewing adherence tools: A scoping review investigating content validity. Patient Educ Couns. 2019;102(12):2145-55.
33. Hurlocker MC, Madson MB, Schumacher JA. Motivational interviewing quality assurance: A systematic review of assessment tools across research contexts. Clin Psychol Rev. 2020;82:101909.
34. Klein DA, Paradise SL, Landis CA. Screening and counseling adolescents and young adults: a framework for comprehensive care. Am Fam Physician. 2020;101(3):147-58.
35. Nemoto T, Beglar D, editors. Likert-scale questionnaires. JALT 2013 conference proceedings; 2014
36. Batterton KA, Hale KN. The Likert scale what it is and how to use it. Phalanx. 2017;50(2):32-9.
37. Agung FH SR, Sudarsono NC, Hendarto A, Werdhani RA, Pudjiati R, Hanum L, Naufal A, Sawyer SM Development and Validation of The Adolescent Behavioral Change Counseling Assessment Tool. BMC Heatlh Serv Res (Under Review).
38. Bujang MA, Baharum N. A simplified guide to determination of sample size requirements for estimating the value of intraclass correlation coefficient: a review. Arch Orofac Sci. 2017;12(1).
39. Juhnke GA. Mental Health Counseling Assessment: Broadening One's Understanding of the Client and the Clients Presenting Concerns. ERIC Digest. 1995.
40. Kamper SJ, Maher CG, Mackay G. Global rating of change scales: a review of strengths and weaknesses and considerations for design. J Man Manip Ther. 2009;17(3):163-70.
41. Al Shorbaji N, Atun R, Car J, Majeed A, Wheeler EL, Beck D, et al. eLearning for undergraduate health professional education: a systematic review informing a radical transformation of health workforce development: World Health Organization; 2015.
42. Barteit S, Guzek D, Jahn A, Bärnighausen T, Jorge MM, Neuhann F. Evaluation of e-learning for medical education in low-and middle-income countries: A systematic review. Comput Educ. 2020;145:103726.
43. Antognoli EL, Seeholzer EL, Gullett H, Jackson B, Smith S, Flocke SA. Primary care resident training for obesity, nutrition, and physical activity counseling: a mixed-methods study. Health Promot Pract. 2017;18(5):672-80.
44. Richmond H, Copsey B, Hall AM, Davies D, Lamb SE. A systematic review and meta-analysis of online versus alternative methods for training licensed health care professionals to deliver clinical interventions. BMC Med Educ. 2017;17(1):1-14.
45. Wandera S. Continuing the conversation about face-to-face, online, and blended learning a meta-analysis of empirical literature 2006-2017: Wilmington University (Delaware); 2017.
46. Liu Q, Peng W, Zhang F, Hu R, Li Y, Yan W. The effectiveness of blended learning in health professions: systematic review and meta-analysis. J Medical Internet Res. 2016;18(1):e4807.
47. Crowley J, Ball L, Hiddink GJ. Nutrition in medical education: a systematic review. Lancet Planet Health. 2019;3(9):e379-e89.
48. Driska AP, Gould DR. Evaluating a problem-based group learning strategy for online, graduate-level coach education. Kinesiol Rev. 2014;3(4):227-34.

Figure 1. Study design

Figure 2. CONSORT diagram

Table 1. Nutritional status and lifestyles of health professionals, parents and adolescents

.	Health Professionals		Parents		Adolescents	
	Intervention Group n = 31 (%)	Control Group n = 32 (%)	Intervention Group n = 46 (%)	Control Group n=68(%)	Intervention Group n=65(%)	Control Group n=68(%)
**Weight status**						
Normal weight	11 (35.5)	13 (40.6)	17 (37)	29 (42.6)	0 (0)	0 (0)
Overweight	15 (48.3)	8 (25)	14 (30.4)	22 (32.3)	5 (7.7)	6 (8.8)
Obese	5 (16.2)	11 (34.4)	15 (32.6)	15 (22.1)	60 (92.3)	62 (91.2)
**Diet**[1]						
Diversity of staple foods [2]						
Yes	3(9.7)	3 (9.4)	14 (30.4)	34 (50)	21 (32.3)	24 (35.3)
No	28(90.3)	29 (90.6)	32 (69.6)	34 (50)	44 (67.7)	44 (64.7)
Healthy animal-based food [3]						
Yes	3 (9.7)	3 (9.4)	3 (6.5)	7 (10.3)	5 (7.8)	9 (13.2)
No	28 (90.3)	28 (90.6)	43 (93.5)	61 (89.7)	60 (92.2)	59 (86.8)
Healthy plant-based food[3]						
Yes	5 (16.1)	5 (15.6)	3 (6.5)	7 (10.3)	5 (7.7)	5 (7.4)
No	26 (83.9)	27 (84.4)	43 (93.5)	61 (89.7)	60 (92.3)	63 (92.6)
Vegetables[4]						
Yes	17 (54.8)	21 (65.6)	26 (56.5)	39 (57.3)	9 (13.8)	7 (10.3)
No	14 (45.2)	11 (34.4)	20 (43.5)	29 (42.7)	56 (86.2)	61 (89.7)
Fruit [4]						
Yes	11 (35.5)	8 (25)	11 (23.9)	18 (26.5)	13 (20)	11 (16.1)
No	20 (64.5)	24 (75)	35 (76.1)	50 (73.5)	52 (80)	57 (83.9)
Healthy snacks [5]						
Yes	3 (9.7)	0 (0)	3 (6.5)	4 (5.9)	0 (0)	0 (0)
No	28 (90.3)	32 (100)	43 (93.5)	64 (94.1)	65 (100)	68 (100)
Fast food [6]						
No	17 (54.8)	20 (62.5)	26 (56.5)	40 (58.8)	17 (26.1)	29 (42.6)
Yes	14 (45.2)	12 (37.5)	18 (43.5)	28 (41.2)	48 (73.9)	39 (57.4)
Instant food [7]						
No	20 (64.5)	21 (65.6)	27 (58.7)	27 (39.7)	20 (30.8)	13 (19.1)
Yes	12 (35.5)	10 (34.4)	19 (41.3)	41 (60.3)	45 (69.2)	55 (80.9)
Sweet drink [8]						
No	13 (41.9)	12 (37.5)	17 (36.9)	18 (26.5)	11 (16.9)	8 (11.8)
Yes	18 (58.1)	20 (62.5)	29 (63.1)	50 (73.5)	54 (83.1)	60 (88.2)
Cooking routine at home [9]						
Yes	22 (71)	20 (62.5)	42 (91.3)	54 (79.4)	42 (64.6)	47 (69.1)
No	9 (29)	12 (37.5)	4 (8.7)	14 (20.6)	23 (35.4)	21 (30.9)
**Level of Physical activity**[10]						
Low	22 (71)	20 (62.5)	22 (47.8)	28 (40)	36 (55.3)	37 (54.4)
Moderate	9 (29)	12 (37.5)	21 (45.7)	36 (52)	25 (38.5)	23 (33.8)

High	7 (22)	8 (25)	3 (6.5)	4 (8)	4 (6.2)	8 (11.8)

[1]Using the Food Frequency Questionnaire in the last week; [2]There is staple food native to Indonesia other than rice > 1x per week; [2]Various animal and vegetable side dishes (including fish and red meat, tofu, tempeh / beans) and fried side dishes no more than once per week; [3]Large portions and frequency of fruits and vegetables according to balanced nutrition recommendations, [4]Healthy snacks: no packaged snacks / high sugar / salt more than 1x per week, [5]Fast food no more than 1x/week; [6]Instant noodles or other instant food no more than 1x/week; [7]Sugary drinks no more than 1x per week; [8]Cook 5x or more in last week; [9]Physical activity level based on the short version of the International Physical Activity Questionnaire.* Despite multiple efforts, only 46 of the 64 parents in the IG completed the questionnaire.

Table 2. Comparison of the first and second knowledge tests, by group.

Knowledge value	Control Group $\bar{x} \pm SD$	Intervention Group $\bar{x} \pm SD$	*p* value (t-test, unpaired group)
First test (pre-training)	63.7 ± 11.3	64.0 ± 9.9	0.902
Second test (post-training for the intervention group)	67.2 ± 9.9	79.1±12.3	< 0.001

Table 3. Assessment of 1st and 2nd audio-recordings using the ABC-CAT, by group

	Scoring Item	Pre-Training / 1st Counseling			Post-Training / 2nd Counseling		
		Control Group	Intervention Group	*p- value**	Control Group	Intervention Group	*p-value**
Introduction							
1	Greets the adolescent and parent/s	1.77 ± 1.11	1.85 ±0.88	0.641	1.79 ± 1.06	1.69 ±0,95	0.609
2	Asks 1) the issue / chief complaint and 2) a brief history of complaints (1-3 questions) AND 3) provides introduction to the counseling session	1.28 ± 0,88	1.55 ±0.62	0.470	1.66 ± 0.95	2.29 ±0.64	< 0.001
Psychosocial Screening							
3	Asks the adolescent to talk alone without their parent, accompanied by a confidentiality statement	0.98 ±1.23	1.32 ±0.96	0.098	0.64 ± 0.86	1.15 ±1.07	0.005
4	Undertakes psychosocial screening	1.36 ±1.23	1.55 ±0.11	0.373	1.75 ± 1.11	2.19 ± 0.72	0.010
Basic communication skill based on MI							
5	Open-ended questions	1.98 ± 0.95	2.12 ±0.86	0.420	2.13 ± 1.01	2.63 ± 0.61	0.001
6	Affirmation	1.19 ± 1.18	1.35 ±1.19	0.447	1.87 ± 1.15	2.44 ± 0.84	0.002
7	Reflective listening	1.52 ± 0.99	1.77 ±0.85	0.134	1.92 ± 1.05	2.53 ± 0.69	< 0.001
8	Summary	0.72 ± 092	0.90 ±0.91	0.273	1.46 ± 1.16	2.18 ± 0.91	< 0.001
MI stages							
9	Engaging	0.920 ± 0.92	0.930 ±0.99	0.089	1.64 ± 1.11	2.37 ± 0.83	< 0.001
10	Focusing	1.3 ± 1.00	1.52 ± 1.00	0.090	1.43 ± 1.12	2.34 ± 0.78	< 0.001
11	Evoking	0.92 ± 1.12	1.05 ± 1.15	0.531	1.07 ± 1.03	2.15 ± 0.88	< 0.001
12	Planning	1.00 ± 0.89	1.00 ± 0.97	1	1.20 ± 1.12	2.31 ± 0.80	< 0.001
13	Provides information with elicit-provide-elicit technique	1.17 ± 0.52	1.33 ± 0.75	0.165	1.38 ± 0.93	1.71 ± 1.01	0.061
Parental Involvement							
14	Parental involvement	0.98 ± 0.81	1.05 ± 0.85	0.660	0.95 ± 0.81	0.84 ± 0.85	0.455
**Mean**		1.25 ± 0.54	1.43 ± 0.55	**0.074**	1.49 ± 0.70	2.05 ± 0.42	**< 0.001**

* *t-test, unpaired group*

Manuscript ver 2.0 Click here to access/download;Manuscript;Effectiveness paper_2.0 plos one.docx

Adolescent weight management counseling: the effectiveness of an online training program for primary healthcare professionals in Indonesia

Fransisca Handy Agung,[a,] Rini Sekartini,[b] Nani Sudarsono,[c] Aryono Hendarto,[b] Retno Asti Werdhani,[c] Meita Dhamayanti,[d] Retno Pudjiati,[e] Lathifah Hanum,[e] Affan Naufal,[f] Susan M Sawyer [g]

Affiliation

[a] Faculty of Medicine, Universitas Pelita Harapan

Jl. Jend. Sudirman No.20, Bencongan, Kelapa Dua, Tangerang, Banten, Indonesia 15810

[b] Department of Child Health, Faculty of Medicine Universitas Indonesia

Jl. Salemba Raya No.6 Jakarta Pusat, Jakarta, Indonesia 10430

[c] Department of Community Medicine, Faculty of Medicine Universitas Indonesia

Jl. Salemba Raya No.6 Jakarta Pusat, Jakarta, Indonesia 10430

[d] Department of Child Health, Faculty of Medicine Universitas Padjajaran,

Jl. Professor Eyckman No.38, Pasteur, Bandung, West Java, Indonesia

[e] Faculty of Psychology Universitas Indonesia,

Kampus UI, Depok, West Java, Indonesia

[f] Balaraja District Hospital

Jl. Rumah Sakit No. 88, Tangerang, Banten, Indonesia

[g] Centre for Adolescent Health, Royal Children's Hospital; Murdoch Children's Research

Institute; and Department of Paediatrics, University of Melbourne, 50 Flemington Road, Parkville VIC 3052, Australia

Email address and ORCID ID

Fransisca Handy Agung: Fransisca.agung@uph.edu ORCID ID 0000-0003-3516-6089

Rini Sekartini rsekartini@yahoo.com ORCID ID 0000-0001-9859-5468

Nani Cahyani Sudarsono nani.cahyani@ui.ac.id ORCID ID 0000-0001-6856-0694

Aryono Hendarto aryhendarto@yahoo.com ORCID ID 0000-0002-7714-8332

Retno Asti Werdhani retnoasti@yahoo.com ORCID ID 0000-0002-3280-4295

Retno Pudjiati, retno_pudjiati@yahoo.com ORCID 0000-0001-5231-7139

Meita Dhamayanti meita_d@yahoo.com ORCID ID 0000-0001-9661-0708

Lathifah Hanum, lathifah.hanum08@ui.ac.id ORCID ID 0000-0003-1771-335X

Affan Naufal, affanaufal@gmail.com ORCID ID 0000-0001-5701-9026

Susan Sawyer: susan.sawyer@rch.org.au ORCID ID 0000-0002-9095-358X

Corresponding author:

Fransisca Handy Agung

Fransisca.agung@uph.edu

Faculty of Medicine Universitas Pelita Harapan

Jl. Jendral Sudirman No 20, Bencongan, Tangerang, Indonesia 15810

ABSTRACT

Background: Overweight and obesity are growing public health concerns globally for which innovative prevention and care delivery efforts are required. We recently developed a web-based training program to improve the quality of health professionals' weight management counseling of adolescents in Indonesia. Having previously confirmed its acceptability, this study aimed to measure the effectiveness of the program through a randomized controlled trial.

Methods: We recruited 64 primary healthcare professionals from 17 provinces across Indonesia who were randomized to participate in a 4-week online training (intervention group [IG, n=32] or a waitlist control group [CG, n=32]. Using active learning approaches, the training program focused on adolescent development, psychosocial assessment, motivational interviewing (MI), and parent engagement. Participants in each arm were asked to record two counseling sessions with adolescents. These were objectively rated by trained psychologists using a validated tool, and also by qualitative assessments of counseling quality. In both groups, the first recorded counseling session occurred before the training. The second recording occurred after the training for IG participants, but not for CG participants.

Results: IG participants demonstrated significant improvements in knowledge and counseling skills ($p<0.001$, t-test). This included improvements in introductory remarks, quality of psychosocial assessment, and MI skills. There was no change in the extent of parental involvement. The MI training successfully oriented the counseling sessions towards a more collaborative and participatory conversation for supporting behavioral change.

Conclusion: This novel online training program improved the knowledge and counseling skills of Indonesian primary healthcare professionals. Greater emphasis on engaging parents and more guidance on conducting telehealth counseling may improve parental involvement in future iterations.

Keywords: adolescents, behavior change, counseling skills, healthy lifestyle, internet-based training, obesity, weight management

INTRODUCTION

Globally, the prevalence of obesity in children and adolescents has tripled in the last four decades. [1] Low- and middle-income countries have experienced a very rapid recent acceleration of obesity in children and adolescents. [2] This is exemplified by Indonesia, a country of 250 million people and over 35 million adolescents aged 10-18 years,[3] whose prevalence of overweight or obesity has increased ten times in the past decade,[4] even before the anticipated escalation following the COVID-19 pandemic.[5]

Overweight at a young age carries a high risk of living with overweight or obesity in adulthood [6], and brings forward the timing of associated ‘adult’ conditions such as cardiovascular disease and diabetes. [7, 8] The prevalence of these non-communicable diseases has also been increasing in adolescent populations.[9] This is particularly concerning given that already in Indonesia, cardiovascular-metabolic diseases such as stroke, heart disease, and diabetes are the leading causes of mortality and morbidity.[10].

Adolescence is a critical period for managing obesity due to the dynamic physiological and psychological changes inherent to this period of growth and development.[11, 12] While behavior change is central to clinical approaches to managing obesity at any age,[11, 13] adolescence heralds growing individual autonomy and capacity to regulate health-related behaviors, such as food choices and physical activity.[13] However, behavior change interventions to address behavior-related health problems are underutilized in healthcare settings and health professionals are poorly prepared to provide adequate counseling to adults over their healthiest weight, [7, 9] let alone adolescents.

Motivational Interviewing (MI) is a clinical approach for behavior change counseling [14, 15]. It is the most widely used counseling technique in the field of weight management, including for adolescents in primary care.[16-18]. MI is a patient-centered counseling technique for exploring patient motivation and strengths for change. It is a collaborative approach that aims

to help patients set and reach their own goals. [15, 19]. To increase the likelihood of behavior change, it is also important to consider the social and physical environments, including the availability and accessibility of resources that support more healthy lifestyles.[20] Further, as parents are critical influencers of social and physical home environments for adolescents, [21, 22] their involvement in adolescent weight management is widely recommended.[23]

In Indonesia, there is no specific teaching on behavioral counseling in medical [24] and nursing schools [25]. Furthermore, there is an absence of continuing medical education programs to help primary healthcare providers develop the necessary attitudes, knowledge and skills to address behavior change in adolescents who are over their healthiest weight. To provide accessible training across a huge archipelago like Indonesia, and in the context of COVID-19, we recently developed an internet-based training program to promote behavior change counseling for weight management in adolescents. Previously, the content and mode of delivery of the training pilot was rated highly by participants.[26] The aim of the current paper is to assess the effectiveness of this training program through a randomized controlled trial of primary healthcare professionals to understand its feasibility and identify areas of refinement.

METHODS

The study design was a two-arm randomized controlled trial that recruited 64 health professionals from primary healthcare centers in 17 of Indonesia's 34 provinces. Following recruitment to the study, participants (primary healthcare doctors and nurses) were randomized to receive the training (intervention group [IG] or to a waitlist control group [CG]) based on their district. At baseline and post-intervention, a pre- and post-training knowledge test was administered and two audio-recorded counseling sessions with adolescents with overweight or obesity and their parents were submitted by participants for assessment. The primary outcome of the training was change in health professional knowledge of behavior change counseling,

while the secondary outcome was change in counseling skills, using objective ratings of each audio-recording that was made by trained professional observers (clinical psychologists) using a specially designed assessment measure. Following submission of their pre-training audio-recording, the IG participated in the 4-week training program, and then audio-recorded a second counseling session with the same two adolescents. The CG participants submitted their second audio-recording at the same time as the IG, prior to them undertaking the training (see Figure 1).

[Insert Figure 1. Study design]

Recruitment of Study Participant (Health Professionals) and Adolescents

The sample size was determined using the mean-difference formula[27] from an RCT on motivational interviewing for obesity.[28] Based on this calculation, 60 adolescents were required for each group to assess counselling effectiveness.[28] We undertook a brief survey of health professionals who considered that they could recruit up to two adolescents each, suggesting that we needed at least 30 health professionals for each group (intervention group [IG], n=32) and waitlist control group [CG], n=32). Healthcare professionals who had routinely provided adolescent health services at a primary health care center for at least 6 months were recruited on February 1st – 15th 2022. Participants were the person in-charge of adolescent friendly health service (AFHS) at the primary health care centers nominated by their provincial health offices. These were mostly midwives, nurses and general practitioners. Of these, we selected only the nurses and general practitioners as adolescent nutrition was one of their competencies, but not for midwives. Participants were provided verbal informed consent by phone which followed by written informed consent in the website. These participants completed a baseline survey about their sociodemographic characteristics, personal nutritional status and eating habits, level of physical activities and their own parenting style (for those who

were parents). Each participant was then required to identify two adolescents with BMI > 85th percentile (WHO, 2007). Participants were asked to explain the study to each adolescent and their parent/s and gain their verbal consent to participate in the study. Prior to the audio-recording of counseling sessions, written informed consent was obtained from both adolescents and parent/s. All resources were available through a website that was specially developed for the study (ramahremaja.id®) which was also used to upload materials (i.e., signed consent forms) and assessments.

The training program

The training program was internet-based and consisted of synchronous and non-synchronous sessions (blended learning) that were completed over four weeks across March to April 2022. Following a needs assessment,[29] the training program was developed using constructive alignment theory to ensure the alignment between the intended learning outcomes, learning activities and assessment tasks.[30] It consisted of four main topics: adolescent growth and development, parenting adolescents, healthy eating and physical activity, and behavioral change counseling techniques for adolescent patients using MI approaches. Each topic consisted of individual learning tasks and an online meeting with all participants. Active learning principles underpinned the development of all learning activities, which utilized multifaceted materials including explanatory videos, audio recordings of exemplar consultations with adolescents, quizzes, virtual interactive case vignettes, and written assignments (e.g., develop a healthy meal plan, develop a schedule for physical activity, map local healthy lifestyle resources, develop a personal behavioral change plan). The agenda for each weekly online meeting consisted of group work, a group presentation and an interactive discussion lead by the facilitator. In the last online meeting, participants engaged in a formal role-play with adolescents in a small group setting. Constructive feedback was provided to each participant, with each member of the group learning from feedback to others.

Knowledge Assessment

The primary outcome was improved health professional knowledge of behavior-change counseling for weight management of adolescents. This was assessed using a 47-item questionnaire that was specifically developed for the study that consisted of questions exploring knowledge of healthy eating, physical activity, screen time, basic communication skills with adolescent patients, psychosocial screening, motivational interviewing techniques and parent roles in adolescent behavior change. Content validity was determined qualitatively by nine experts (clinicians, academics, members of relevant professional organizations and representatives of the Ministry of Health) to ensure that the content was consistent with current national guidelines. Following this, face validity was undertaken by 11 Indonesian AFHS national trainers. Item analysis was done by calculating the Item Difficulty Index (IDI) using the average score of each item and Discrimination Index (DI) using Point of Biserial coefficient correlation in a sample of 67 health workers from 30 out of 34 provinces in Indonesia.[31] The item analysis procedure was followed by consideration of the learning objectives and materials and led to revision of some items. The final knowledge assessment tool had a Cronbach-α 0.786, indicating good internal consistency.

Counseling Skill Assessment

The secondary outcome was improved counseling skills for weight management of adolescent patients. Assessment of counseling skills was evaluated both quantitatively and qualitatively by trained professional raters, as well as by participant self-assessment.

Quantitative assessment

Assessment tools were specially developed for this study, based on the content of the training module. With the plan of using trained professional raters (psychologists), the assessment tool was modified from the Behavioral Change Counseling Index (BECCI), a commonly used tool for evaluating behavioral-change counseling quality in healthcare settings.[32, 33] Three

further items were added which were specific for counseling sessions with adolescent patients, namely: greeting adolescents and their parents; inviting adolescents to talk on their own without their parents (including a confidentiality statement);[34] and, discussing the approach to parental involvement with the parents. The scoring for each item in the assessment tool was also modified from the original BECCI. Instead of a 5-point Likert scale, our assessment tool used a 4-point Likert scale with slightly modified explanations of each item with the intention of improving its objectivity.[35, 36] A rating of 0 was the lowest (no statements heard) with 3 being the highest (all required statements heard). The assessment tool was called the Adolescent Behavioral Change-Counseling Assessment Tool (ABC-CAT).[37]

The ABC-CAT underwent a validation process. Firstly, content validity was undertaken by nine experts, followed by assessment of face validity by ten clinical psychologists. Construct validity was undertaken with corrected correlation coefficient analysis (n=125) using the first counseling session review from both groups (IG and CG). The inter-item corrected value was more than 0.3 for all items and Cronbach-α 0.839, which revealed good validity and good internal consistency.[37]

The final ABC-CAT[37] assessed four domains: 1) Introduction (a greeting and how to start the counseling session); 2) Psychosocial screening (this commenced by inviting the adolescent to talk without their parents, included a confidentiality statement prior to the psychosocial screening which focused on evaluating the social determinants of healthy weight); 3) MI basic communication skills and MI stages (engaging, focusing, evoking and planning); and 4) Parental involvement (this evaluated whether the participants discussed any of two parenting roles, namely around structuring and supporting behavior change in adolescents).

Eight trained clinical psychologists assessed the audio-taped counseling sessions. These psychologists underwent training for the assessment, after which they each scored 15 audio recordings from an earlier pilot for the study. The inter-rater reliability score (the inter-class

correlation coefficient test using the simplified Winer and Walter formula) was 0.942 (CI 0.868 – 0.973), which showed strong agreement.[38]

Each participant uploaded two sets of two recorded counseling sessions onto the website, four weeks apart. IG participants uploaded an audio-recording of the same two adolescents before and after the training. The CG participants also uploaded two audio-recordings on two occasions of the same two adolescents, but both were prior to training. Each recording was coded and assigned to a rater by an embedded system in the website. The system ensured that each of the four recordings submitted by each participant was reviewed by different raters, who reviewed them blindly.

Qualitative assessment

Qualitative assessment is a flexible, holistic and nonstatistical assessment method where participants can process what they learned from the experience by provision of feedback soon after the counseling session.[39, 40] For each audio-recording, the trained rater was asked to write a narrative description about the strengths and weaknesses of each counseling session and to provide an overall assessment of its quality. This narrative description was made available to each participant, confidentially, through their training portal in the website.

Data Analysis

A summary of baseline data of the participants was developed to show the baseline similarities of each group. To evaluate the effectiveness of the training, we compared changes in knowledge and skills between each group. Differences between pre- and post-intervention knowledge scores were assessed by group. Change in skills was assessed quantitatively using differences in the ABC-CAT assessment questionnaire and also qualitatively. Continuous variables were described by standard descriptive statistics containing mean and standard deviation. Comparison between groups was done using the unpaired t-test.

RESULTS

Sample characteristics

A CONSORT diagram that details recruitment and retention is shown in Figure 2. A total of 64 health professionals (52 females) were recruited, which comprised 35 nurses and 29 physicians. Fourteen (22%) reported some prior training in adolescent friendly health services (AFHS). The adolescent patients who consented to be audiotaped were similar in age and sex by group. Each adolescent was required to be accompanied by one of their parents. The parents were predominantly mothers, although there were 17 fathers in each group. As show in Table 1, around two-thirds of health professionals and parents themselves experienced overweight or obesity. The predominant weight category for adolescents was obesity rather than overweight (IG, 92%: CG, 91%).

[Insert Figure 2. CONSORT diagram]

In both adult populations (health professionals and parents) a proportion of participants had unhealthy eating habits (low fruit consumption, deep fried food, sugary beverages, ultra-processed food and junk meal). Mostly, this was eating vegetables less frequently than recommended and reporting unhealthy snacking. A higher proportion of adolescents than parents reported low consumption of vegetables and high levels of unhealthy snacking. Adolescents also commonly reported a variety of other unhealthy eating behaviors. More than half of the adolescents had low levels of physical activity. For each of these lifestyle characteristics, there were no significant differences between adolescents by group (Table 1).

Table 1. Nutritional status and lifestyles of health professionals, parents and adolescents

.	Health Professionals		Parents		Adolescents	
	Intervention Group n = 31 (%)*	Control Group n = 32 (%)*	Intervention Group n = 46 (%)*	Control Group n=68(%)*	Intervention Group n=65(%)*	Control Group n=68(%)*
**Weight status**						
Normal weight	11 (35.5)	13 (40.6)	17 (37)	29 (42.6)	0 (0)	0 (0)
Overweight	15 (48.3)	8 (25)	14 (30.4)	22 (32.3)	5 (7.7)	6 (8.8)
Obese	5 (16.2)	11 (34.4)	15 (32.6)	15 (22.1)	60 (92.3)	62 (91.2)
**Diet**[1]						
Diversity of staple foods [2]						
Yes	3(9.7)	3 (9.4)	14 (30.4)	34 (50)	21 (32.3)	24 (35.3)
No	28(90.3)	29 (90.6)	32 (69.6)	34 (50)	44 (67.7)	44 (64.7)
Healthy animal-based food [3]						
Yes	3 (9.7)	3 (9.4)	3 (6.5)	7 (10.3)	5 (7.8)	9 (13.2)
No	28 (90.3)	28 (90.6)	43 (93.5)	61 (89.7)	60 (92.2)	59 (86.8)
Healthy plant-based food[3]						
Yes	5 (16.1)	5 (15.6)	3 (6.5)	7 (10.3)	5 (7.7)	5 (7.4)
No	26 (83.9)	27 (84.4)	43 (93.5)	61 (89.7)	60 (92.3)	63 (92.6)
Vegetables[4]						
Yes	17 (54.8)	21 (65.6)	26 (56.5)	39 (57.3)	9 (13.8)	7 (10.3)
No	14 (45.2)	11 (34.4)	20 (43.5)	29 (42.7)	56 (86.2)	61 (89.7)
Fruit [4]						
Yes	11 (35.5)	8 (25)	11 (23.9)	18 (26.5)	13 (20)	11 (16.1)
No	20 (64.5)	24 (75)	35 (76.1)	50 (73.5)	52 (80)	57 (83.9)
Healthy snacks [5]						
Yes	3 (9.7)	0 (0)	3 (6.5)	4 (5.9)	0 (0)	0 (0)
No	28 (90.3)	32 (100)	43 (93.5)	64 (94.1)	65 (100)	68 (100)
Fast food [6]						
No	17 (54.8)	20 (62.5)	26 (56.5)	40 (58.8)	17 (26.1)	29 (42.6)
Yes	14 (45.2)	12 (37.5)	18 (43.5)	28 (41.2)	48 (73.9)	39 (57.4)
Instant food [7]						
No	20 (64.5)	21 (65.6)	27 (58.7)	27 (39.7)	20 (30.8)	13 (19.1)
Yes	12 (35.5)	10 (34.4)	19 (41.3)	41 (60.3)	45 (69.2)	55 (80.9)
Sweet drink [8]						
No	13 (41.9)	12 (37.5)	17 (36.9)	18 (26.5)	11 (16.9)	8 (11.8)
Yes	18 (58.1)	20 (62.5)	29 (63.1)	50 (73.5)	54 (83.1)	60 (88.2)
Cooking routine at home [9]						
Yes	22 (71)	20 (62.5)	42 (91.3)	54 (79.4)	42 (64.6)	47 (69.1)
No	9 (29)	12 (37.5)	4 (8.7)	14 (20.6)	23 (35.4)	21 (30.9)
**Level of Physical activity**[10]						
Low	22 (71)	20 (62.5)	22 (47.8)	28 (40)	36 (55.3)	37 (54.4)
Moderate	9 (29)	12 (37.5)	21 (45.7)	36 (52)	25 (38.5)	23 (33.8)

High	7 (22)	8 (25)	3 (6.5)	4 (8)	4 (6.2)	8 (11.8)

[1]Using the Food Frequency Questionnaire in the last week; [2]There is staple food native to Indonesia other than rice > 1x per week; [2]Various animal and vegetable side dishes (including fish and red meat, tofu, tempeh / beans) and fried side dishes no more than once per week; [3]Large portions and frequency of fruits and vegetables according to balanced nutrition recommendations, [4]Healthy snacks: no packaged snacks / high sugar / salt more than 1x per week, [5]Fast food no more than 1x/week; [6]Instant noodles or other instant food no more than 1x/week; [7]Sugary drinks no more than 1x per week; [8]Cook 5x or more in last week; [9]Physical activity level based on the short version of the International Physical Activity Questionnaire.

*Despite multiple efforts, only 46 out of 64 parents in IG completed the questionnaire and 1 participant from the IG did not complete the questionnaire. There were participants (1 in IG and 4 in CG) who changed their adolescent patients for the post-training / second counseling and these patients were required to submit the questionnaire as well. Thus, there were 65 adolescents in IG and 68 adolescents in CG, instead of 64.

Knowledge Assessment

At baseline, there were no differences in knowledge scores (p = 0.902, unpaired t-test) between IG and CG participants. Four weeks later, the IG had significantly higher scores (p < 0.001, unpaired t-test) than the CG (Table 2).

Table 2. Comparison of the first and second knowledge tests, by group.

Knowledge value	Control Group $\bar{x}$ ± SD	Intervention Group $\bar{x}$ ± SD	*p* value (t-test, unpaired group)
First test (pre-training)	63.7 ± 11.3	64.0 ± 9.9	0.902
Second test (post-training for the intervention group)	67.2 ± 9.9	79.1±12.3	< 0.001

Counseling skill assessment

Quantitative assessment (ABC-CAT)

As part of training and assessment, participants engaged in a counseling session with adolescents, which they audio-recorded and uploaded to the study website. Due to the COVID-19 pandemic, participants were free to conduct counseling sessions remotely by video (e.g., zoom, telephone) or face-to-face at the clinic. A total of 256 sessions were uploaded, 128 sessions from each group. Five of 64 adolescents did not return for the 2nd counselling session (IG, n=1; CG, n=4); two did not respond to follow-up texts and phone calls, two had moved to

another city, and one was hospitalized due to a traffic injury. Instead, audio recordings were made with other adolescents who met the study criteria. A total of 116 (45%) sessions were undertaken virtually, which were similarly distributed by group (IG 47%, CG 44%). At baseline, there were no differences in assessment by group (Table 3). Assessment of the 2nd audio-recording revealed significant differences ($p <0.001$, unpaired t-test) in the counselling quality of participants in the IG compared to the CG, as measured by the ABC-CAT domains. Significant differences were seen in relation to both aspects of psychosocial screening, namely the provision of a confidentiality assessment and psychosocial screening itself. There was a highly significant difference between the two groups for all but one assessment item for MI (the elicit-provide-elicit skill just failed to reach significance, $p=0.061$). There were no differences between groups in the use of introductory greetings ($p=0.609$) or in the extent of parental involvement by group ($p= 0.455$).

Table 3. Assessment of 1st and 2nd audio-recordings using the ABC-CAT, by group

Scoring Item	Pre-Training / 1st Counseling			Post-Training / 2nd Counseling		
	Control Group	Intervention Group	*p- value**	Control Group	Intervention Group	*p-value**
Introduction						
1 Greets the adolescent and parent/s	1.77 ± 1.11	1.85 ±0.88	0.641	1.79 ± 1.06	1.69 ±0,95	0.609
2 Asks 1) the issue / chief complaint and 2) a brief history of complaints (1-3 questions) AND 3) provides introduction to the counseling session	1.28 ± 0,88	1.55 ±0.62	0.470	1.66 ± 0.95	2.29 ±0.64	< 0.001
Psychosocial Screening						
3 Asks the adolescent to talk alone without their parent, accompanied by a confidentiality statement	0.98 ±1.23	1.32 ±0.96	0.098	0.64 ± 0.86	1.15 ±1.07	0.005
4 Undertakes psychosocial screening	1.36 ±1.23	1.55 ±0.11	0.373	1.75 ± 1.11	2.19 ± 0.72	0.010
Basic communication skill based on MI						
5 Open-ended questions	1.98 ± 0.95	2.12 ±0.86	0.420	2.13 ± 1.01	2.63 ± 0.61	0.001
6 Affirmation	1.19 ± 1.18	1.35 ±1.19	0.447	1.87 ± 1.15	2.44 ± 0.84	0.002
7 Reflective listening	1.52 ± 0.99	1.77 ±0.85	0.134	1.92 ± 1.05	2.53 ± 0.69	< 0.001
8 Summary	0.72 ± 092	0.90 ±0.91	0.273	1.46 ± 1.16	2.18 ± 0.91	< 0.001
MI stages						
9 Engaging	0.920 ± 0.92	0.930 ±0.99	0.089	1.64 ± 1.11	2.37 ± 0.83	< 0.001
10 Focusing	1.3 ± 1.00	1.52 ± 1.00	0.090	1.43 ± 1.12	2.34 ± 0.78	< 0.001
11 Evoking	0.92 ± 1.12	1.05 ± 1.15	0.531	1.07 ± 1.03	2.15 ± 0.88	< 0.001
12 Planning	1.00 ± 0.89	1.00 ± 0.97	1	1.20 ± 1.12	2.31 ± 0.80	< 0.001
13 Provides information with elicit-provide-elicit technique	1.17 ± 0.52	1.33 ± 0.75	0.165	1.38 ± 0.93	1.71 ± 1.01	0.061
Parental Involvement						
14 Parental involvement	0.98 ± 0.81	1.05 ± 0.85	0.660	0.95 ± 0.81	0.84 ± 0.85	0.455
**Mean**	1.25 ± 0.54	1.43 ± 0.55	**0.074**	1.49 ± 0.70	2.05 ± 0.42	**< 0.001**

* *t-test, unpaired group*

Qualitative Assessment

At baseline, the narrative assessment of communication techniques by qualified psychologists (who were blinded to both the timing [pre/post] and group) suggested there were substantial opportunities for improvement by participants in both groups. The consultations were commonly stilted, with little evidence of a two-way conversation that actively engaged adolescents in the discussion, let alone showed evidence of goal setting or planning for change.

The counseling session felt interrogative, so the conversation went one way and tended to be monotonous. It would be better if the adolescent was more actively involved in determining their own changes in eating patterns and activities using the MI technique (Counseling 1, CG, Participant 25, Psychologist G).

In some sections of the counseling session, the health worker sounded confused about what else to ask the adolescent. To minimize their confusion, the health worker could reflect more on the adolescent's statements and use this to lead to behavioral changes (Counseling 1, IG, Participant 5, Psychologist E).

After the training, assessor comments suggest there were differences between participants by group. IG participants used more communication techniques, especially more open questions and reflective listening. There was also good appreciation of the different MI stages, with the exception of evoking skills.

It was apparent that the adolescent patient was fully involved in a two-way discussion regarding changes in her behavior, especially in developing diet and activity plans that the adolescent could do on her own (Counseling 2, IG, Participant 4, Psychologist D).

[The participant] conducted an individual session with the adolescent, ensured medical confidentiality and asked open-ended questions that made the adolescent feel comfortable. The health worker applied MI techniques, especially around the engaging process, and involved the adolescent in a two-way discussion, evoking using scaling method and inviting the adolescent to plan for change, again using a two-way conversation (Counseling 2, IG, Participant 31, Psychologist A).

The preparatory steps required for a psychosocial assessment, such as providing the opportunity for adolescents to talk with the health professional alone and making a confidentiality statement, were less well done in comparison to the behavioral change counseling steps. Further, there was no significant difference ($p = 0.455$, unpaired t-test) in the

involvement of parents by group. IG participants provided greater opportunities for adolescents to share their stories and to develop two-way participatory conversations in relation to developing a behavior-change plan.

DISCUSSION

To our knowledge, this is the first online training program that has been developed to enhance counseling skills for primary health professionals caring for adolescents with overweight and obesity in Indonesia, a highly populous low-income country from which health professionals could greatly benefit from effective training interventions. Using a randomized controlled trial that was supported by an online web platform, we demonstrated objective improvements in health professional knowledge of counseling approaches, the primary outcome of the study. Importantly, we also showed significant improvements in counselling skills, the secondary outcome, as shown by improved quality of audiotaped counseling sessions with adolescents. We have previously shown excellent acceptability of the training, with participants rating it highly for both the clarity of content and the enjoyment of training methods (manuscript under review, results not presented here).

This training was ambitious. In terms of content, up to four new concepts were introduced to participants across the four-week program. These related to normal adolescent growth and development and how patterns of overweight and obesity differ to this; approaches to engaging adolescents, communicating with adolescents and parents, and taking a confidential psychosocial history; normal patterns of adolescent nutrition and physical activity, consideration of meal plans for parents, and assisting families to map local resources; and motivational interviewing to address motivation, physical and social environments influencing adolescent lifestyle.[20, 29] While we intentionally recruited participants from AFHS in Indonesia with the expectation that they would have reasonable knowledge of at least the first

two concepts, only a fifth of participants reported some form of training in adolescent health. It is interesting to reflect on how the results might have differed had the participants started with higher baseline knowledge and skills, such as understanding how to involve parents within consultations. Beyond complex content, novel approaches to delivery were also utilized. Firstly, this related to the nature of blended learning within the online training program which included synchronous and asynchronous methods. In addition, as the training was conducted during the COVID-19 pandemic, around 45% of counseling session were done remotely, which many participants (and families) had little familiarity with. Notwithstanding the complexity of concepts and context, the extent of active learning approaches designed to enhance knowledge and skills is likely to have contributed to the positive findings from this study.

Despite recognition of the particular value of e-learning and blended learning for health professionals in LMIC, the lack of high-quality studies from LMIC is also well recognized. In 2015, a systematic review by Al-Shorbaji et al. that examined global e-learning and blended learning methods and their effect on knowledge, skills, attitudes and satisfaction found only five studies had been conducted in LMIC.[41] Disappointingly, a more recent systematic evaluation of e-learning for medical education in LMIC from 2020 found that poor quality studies predominated[42]. For example, of 52 reviewed studies, only four (8%) were randomized controlled trials, and only ten (19%) studies assessed change in skills.

Notwithstanding the novelty of content and the complexity of the setting in the peak of the COVID-19 pandemic, there were significant improvements in intervention participants' knowledge and skills, including MI techniques. These findings are consistent with a study by Antognoli et al. who emphasized that clinician confidence in counseling is enhanced by building skills with the opportunity to practice, engage patients, and receive feedback to hone counseling skills,[43] as we also attempted.

We failed to show better parental involvement in developing the behavioral-change plan for their children. Beyond focusing on the individual with overweight, providing parents with the necessary understanding, skills and resources to model more healthy lifestyles for the whole family is a central part of adolescent weight management practices.[20, 29] We do not know the extent to which insignificant differences around engaging parents reflected insufficient focus on parenting within the training program, or whether other factors were responsible, such as parent and professional lack of familiarity with online consultations which may have resulted in reduced engagement of parents during consultations.

As shown in other online training, [44] allowing time for participants to flexibly engage with the learning material is arguably a strength of this training. We also believe that the synchronous weekly meetings which provided a fun, interactive learning environment enabled participants to engage with the facilitators and other participants from across Indonesia, which is likely to have enhanced participant motivation and improved the effectiveness of the training.[45, 46] Health professionals with favorable personal health behaviors are more likely to counsel patient about healthy lifestyles than those with less favorable lifestyle habits and perceived low self-efficacy in nutrition care.[47] The lifestyle characteristics of the health professionals were representative of the Indonesian National Health Survey data on eating behavior and physical activity of the Indonesian population.[4] We hypothesize that the focus on health professionals in relation to their own lifestyles (a hands-on exercise was to develop a personal plan for a meal, physical activity and personal behavior change) may have promoted their self-efficacy in conducting a behavioral change counseling session for adolescents.

Consistent with a systematic review of online learning in LMIC,[42] a major challenge for some participants was poor internet connectivity, as well as low internet literacy in a minority of participants. Following our initial pilot study, efforts were made to reduce the bandwidth required, but other approaches such as gathering participants in a place with good internet

connectivity to download the training material and upload assessment tasks may also be required. Offering the interactive skill building components of the four-week program in a more intensive manner, face-to-face, may also obviate this.

A strength of this study is the care that went into the development of the training program. A needs assessment[29] involving adolescent and their parents from different socio-demographic background was conducted to generate learning objectives and inform the training materials. Using constructive alignment theory [30], careful attention was made to develop the approach to assessment. A nation-wide study informed the development of the knowledge assessment tool and the ABC-CAT was carefully developed and validated as a measure of skill assessment.[37] Objective rating by multiple trained raters of each counseling session was undertaken blindly, managed through the study website. There are also a number of limitations. The study was undertaken during the COVID-19 pandemic, with many health professionals, patients and parents alike being novice users of online consultations. Given the importance of parents in providing the social and physical environments for healthy lifestyle development at home,[20, 29] the lack of improvement in parent engagement within the counseling sessions suggests further study is required to understand whether this reflected the challenges of the pandemic for parents or perhaps a lack of emphasis within the training program. The many concepts and materials that were learnt in a short period raises questions about whether these practices are able to be embedded within daily consultations, something that also warrants further study. Developing a community of practice is one potential strategy to maintain the group's motivation and provide a space to 'trouble shoot' when participants face roadblocks.[48]

CONCLUSION

This carefully designed online training program shows promise in increasing the quality of primary care professionals' weight management counseling skills for adolescents. Future

research is required to determine the impact of improved counseling skills on the actual behaviors of adolescents and their parents. However, this training program, and the website that it is based on, shows potential as a training strategy that warrants further exploration in Indonesia, given the extent of training needs in this highly populous country.

Acknowledgements

The authors thank: Aryo Sudiro from St. Patrick, the e-learning consultant who developed the website; the Ministry of Health of the Republic of Indonesia, especially the director and the staff of The Directorate of Nutrition, Child and Maternal Health; and the provincial health offices from the 17 provinces who assisted with the nationwide pilot. The authors also thank: the Indonesia national AFHS facilitators (Indira Dewi, Rinalco Franky, Ni Made Jendri, Ninin Anggreani, Dian Sharie, Dewi Nur, Risna Yanti, lka Maharani, lndah Ariestanti, Dena Restiana, Asep Zaenal); the clinical psychologist team (Dian Oriza, Annisa Rahmalia, Utari Krisnamurthi, Sri Wulandari, Fina Dwi Putri, Widya Gunawan, Diajeng Tri, Fadhilah Erynanda, Risky Adinda); and Tan Shot Yen, who significantly contributed to the preparation phase of the training material, during and after the training pilot. Lastly, the authors thank all of the nurses and physicians, adolescents and parents who participated in the pilot intervention

REFERENCES

1. World Health Organization. World Health Organization obesity and overweight fact sheet. 2016.
2. Dong Y, Jan C, Ma Y, Dong B, Zou Z, Yang Y, et al. Economic development and the nutritional status of Chinese school-aged children and adolescents from 1995 to 2014: an analysis of five successive national surveys. Lancet Diabetes Endocrinol. 2019;7(4):288-99.
3. Central Bureau of Statistics. Profil anak indonesia 2018. Jakarta: The Ministry of Women Empowerment and Child Protection (MoWEC). 2018.
4. The Indonesian Institute of Health Research and Development. Basic Health Survey 2018. Jakarta: Publishing Body of The Indonesian Institute of Health Research and Development. 2018
5. Stavridou A, Kapsali E, Panagouli E, Thirios A, Polychronis K, Bacopoulou F, et al. Obesity in Children and Adolescents during COVID-19 Pandemic. Children (Basel). 2021;8(2):135.

6. Singh AS, Mulder C, Twisk JW, Van Mechelen W, Chinapaw MJ. Tracking of childhood overweight into adulthood: a systematic review of the literature. Obes Rev. 2008;9(5):474-88.
7. Sommer A, Twig G. The impact of childhood and adolescent obesity on cardiovascular risk in adulthood: a systematic review. Curr Diab Rep. 2018;18:1-6.
8. Weihrauch-Blüher S, Schwarz P, Klusmann J-H. Childhood obesity: increased risk for cardiometabolic disease and cancer in adulthood. Metabolism. 2019;92:147-52.
9. Bendor CD, Bardugo A, Pinhas-Hamiel O, Afek A, Twig G. Cardiovascular morbidity, diabetes and cancer risk among children and adolescents with severe obesity. Cardiovasc Diabetol. 2020;19(1):1-14.
10. Marthias T, Anindya K, Ng N, McPake B, Atun R, Arfyanto H, et al. Impact of non-communicable disease multimorbidity on health service use, catastrophic health expenditure and productivity loss in Indonesia: a population-based panel data analysis study. BMJ Open. 2021;11(2):e041870.
11. Cardel MI, Jastreboff AM, Kelly AS. Treatment of adolescent obesity in 2020. JAMA. 2019;322(17):1707-8.
12. Norris SA, Frongillo EA, Black MM, Dong Y, Fall C, Lampl M, et al. Nutrition in adolescent growth and development. Lancet. 2021.
13. Teixeira PJ, Marques MM. Health behavior change for obesity management. Obes Facts. 2017;10(6):666-73.
14. Resnicow K, McMaster F, Bocian A, Harris D, Zhou Y, Snetselaar L, et al. Motivational interviewing and dietary counseling for obesity in primary care: an RCT. Pediatrics. 2015;135(4):649-57.
15. Rollnick S, Miller WR, Butler C. Motivational interviewing in health care: helping patients change behavior: Guilford press; 2008.
16. Barlow SE. Expert committee recommendations regarding the prevention, assessment, and treatment of child and adolescent overweight and obesity: summary report. Pediatrics. 2007;120(Supplement 4):S164-S92.
17. World Health Organization. Technical package for cardiovascular disease management in primary health care: healthy-lifestyle counselling. World Health Organization; 2018.
18. Vallabhan MK, Jimenez EY, Nash JL, Gonzales-Pacheco D, Coakley KE, Noe SR, et al. Motivational Interviewing to Treat Adolescents With Obesity: A Meta-analysis. Pediatrics. 2018;142(5).
19. Resnicow K, Davis R, Rollnick S. Motivational interviewing for pediatric obesity: conceptual issues and evidence review. J Am Diet Assoc. 2006;106(12):2024-33.
20. Neufeld LM, Andrade EB, Ballonoff Suleiman A, Barker M, Beal T, Blum LS, et al. Food choice in transition: adolescent autonomy, agency, and the food environment. Lancet. 2022;399(10320):185-97.
21. Patton GC, Sawyer SM, Santelli JS, Ross DA, Afifi R, Allen NB, et al. Our future: a Lancet commission on adolescent health and wellbeing. Lancet. 2016;387(10036):2423-78.
22. Wang J, Fielding-Singh P. How food rules at home influence independent adolescent food choices. J Adolesc Health. 2018;63(2):219-26.
23. Pollak KI, Coffman CJ, Tulsky JA, Alexander SC, Østbye T, Farrell D, et al. Teaching physicians motivational interviewing for discussing weight with overweight adolescents. J Adolesc Health. 2016;59(1):96-103.
24. Konsil Kedokteran Indonesia. Standar kompetensi dokter. Jakarta: Konsil Kedokteran Indonesia. 2006.
25. Persatuan Perawat Nasional Indonesia. Standar kompetensi perawat Indonesia. Bidang Organisasi PP-PPNI: Jakarta. 2005.
26. Agung FH S, b R, Sudarsono N, Hendarto A, Werdhani RA, Dhamayanti M, Pudjiati,e R, Hanum, L, Naufal A, Sawyer S. Development of an internet-based intervention to improve health professionals' counseling skills around adolescent weight management in Indonesia. Plos One. 2023;(Under Review).
27. Charan J, Biswas T. How to calculate sample size for different study designs in medical research? Indian journal of psychological medicine. 2013;35(2):121-6.

28. Pakpour AH, Gellert P, Dombrowski SU, Fridlund B. Motivational interviewing with parents for obesity: an RCT. Pediatrics. 2015;135(3):e644-52.
29. Agung FH, Sekartini R, Sudarsono NC, Hendarto A, Dhamayanti M, Werdhani RA, et al. The barriers of home environments for obesity prevention in Indonesian adolescents. BMC Public Health. 2022;22(1):1-10.
30. Biggs J, Tang C. Teaching for quality learning at university: McGraw-hill education (UK); 2011.
31. Quaigrain K, Arhin AK. Using reliability and item analysis to evaluate a teacher-developed test in educational measurement and evaluation. Cogent Education. 2017;4(1):1301013.
32. Lundahl B, Droubay BA, Burke B, Butters RP, Nelford K, Hardy C, et al. Motivational interviewing adherence tools: A scoping review investigating content validity. Patient Educ Couns. 2019;102(12):2145-55.
33. Hurlocker MC, Madson MB, Schumacher JA. Motivational interviewing quality assurance: A systematic review of assessment tools across research contexts. Clin Psychol Rev. 2020;82:101909.
34. Klein DA, Paradise SL, Landis CA. Screening and counseling adolescents and young adults: a framework for comprehensive care. Am Fam Physician. 2020;101(3):147-58.
35. Nemoto T, Beglar D, editors. Likert-scale questionnaires. JALT 2013 conference proceedings; 2014
36. Batterton KA, Hale KN. The Likert scale what it is and how to use it. Phalanx. 2017;50(2):32-9.
37. Agung FH SR, Sudarsono NC, Hendarto A, Werdhani RA, Pudjiati R, Hanum L, Naufal A, Sawyer SM Development and Validation of The Adolescent Behavioral Change Counseling Assessment Tool. BMC Heatlh Serv Res (Under Review).
38. Bujang MA, Baharum N. A simplified guide to determination of sample size requirements for estimating the value of intraclass correlation coefficient: a review. Arch Orofac Sci. 2017;12(1).
39. Juhnke GA. Mental Health Counseling Assessment: Broadening One's Understanding of the Client and the Clients Presenting Concerns. ERIC Digest. 1995.
40. Kamper SJ, Maher CG, Mackay G. Global rating of change scales: a review of strengths and weaknesses and considerations for design. J Man Manip Ther. 2009;17(3):163-70.
41. Al Shorbaji N, Atun R, Car J, Majeed A, Wheeler EL, Beck D, et al. eLearning for undergraduate health professional education: a systematic review informing a radical transformation of health workforce development: World Health Organization; 2015.
42. Barteit S, Guzek D, Jahn A, Bärnighausen T, Jorge MM, Neuhann F. Evaluation of e-learning for medical education in low-and middle-income countries: A systematic review. Comput Educ. 2020;145:103726.
43. Antognoli EL, Seeholzer EL, Gullett H, Jackson B, Smith S, Flocke SA. Primary care resident training for obesity, nutrition, and physical activity counseling: a mixed-methods study. Health Promot Pract. 2017;18(5):672-80.
44. Richmond H, Copsey B, Hall AM, Davies D, Lamb SE. A systematic review and meta-analysis of online versus alternative methods for training licensed health care professionals to deliver clinical interventions. BMC Med Educ. 2017;17(1):1-14.
45. Wandera S. Continuing the conversation about face-to-face, online, and blended learning a meta-analysis of empirical literature 2006-2017: Wilmington University (Delaware); 2017.
46. Liu Q, Peng W, Zhang F, Hu R, Li Y, Yan W. The effectiveness of blended learning in health professions: systematic review and meta-analysis. J Medical Internet Res. 2016;18(1):e4807.
47. Crowley J, Ball L, Hiddink GJ. Nutrition in medical education: a systematic review. Lancet Planet Health. 2019;3(9):e379-e89.
48. Driska AP, Gould DR. Evaluating a problem-based group learning strategy for online, graduate-level coach education. Kinesiol Rev. 2014;3(4):227-34.

Figure
Click here to access/download;Figure;Figure 1.tif

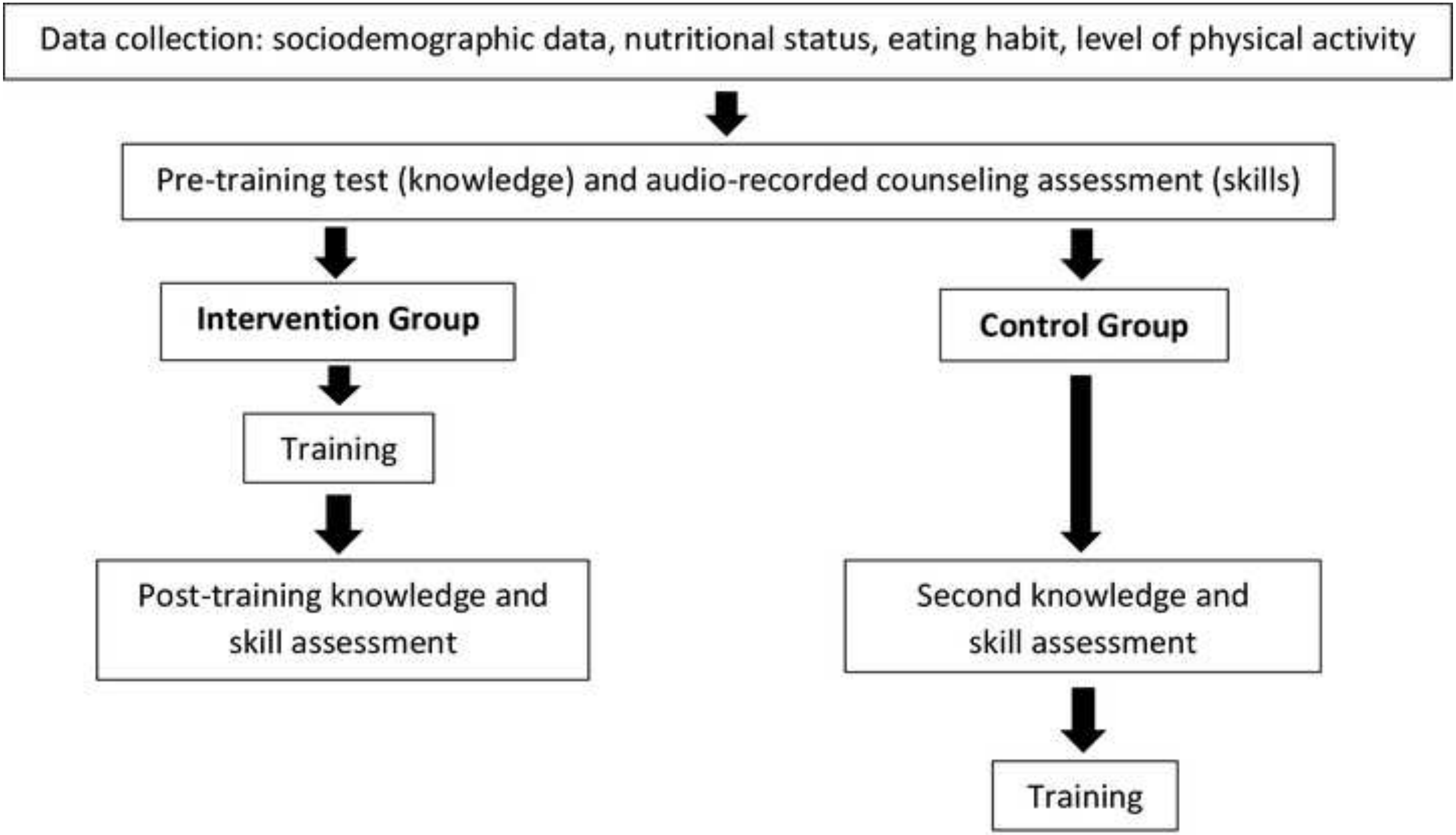

Figure 1. Study design

Click here to access/download;Figure;Figure 2.tif

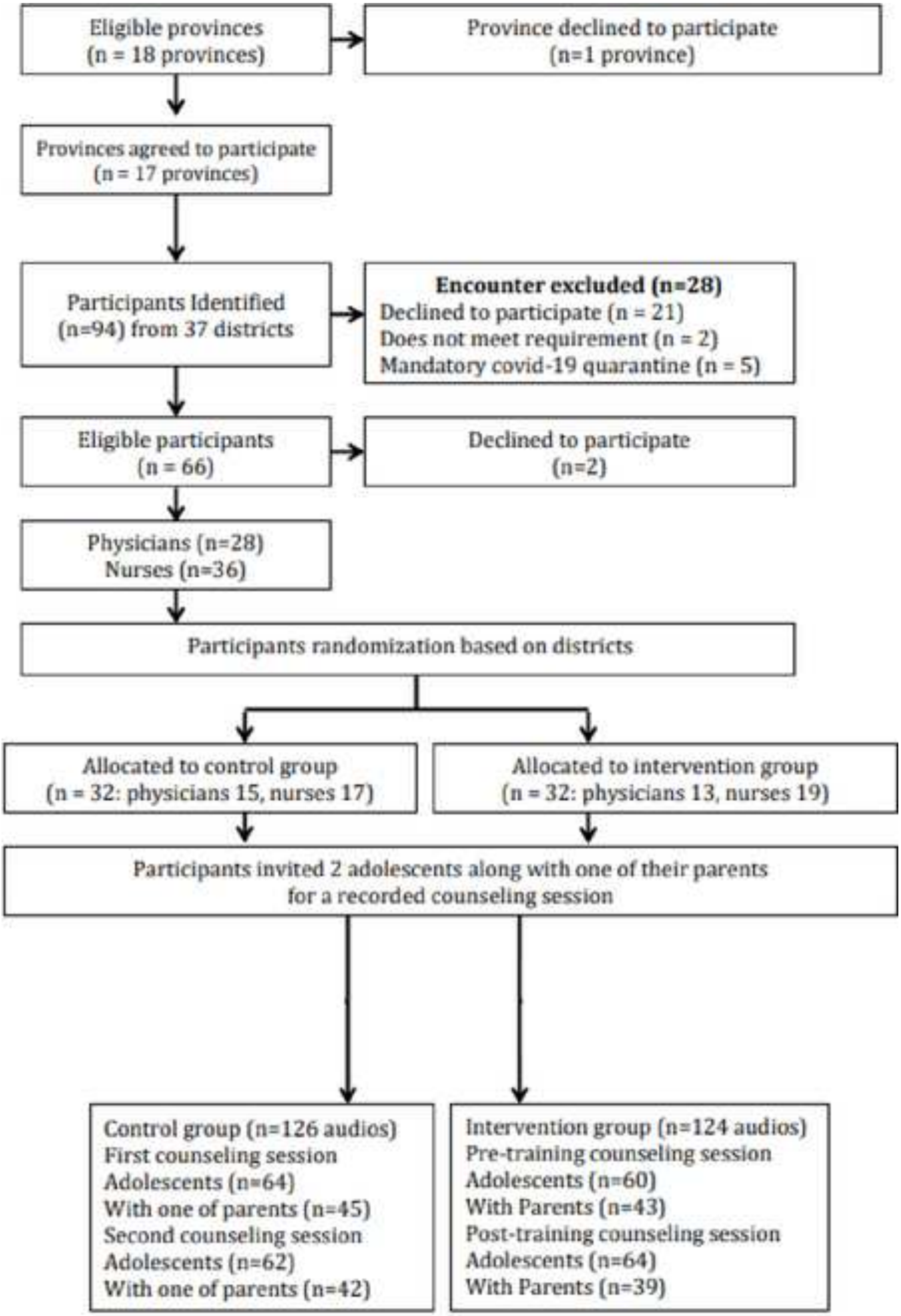

Figure 2. CONSORT diagram

Click here to access/download
Supporting Information
Ethical Approval Fransisca H Agung.pdf
