# Supplementary material for: Adolescent weight management counseling: The effectiveness of an online training program for primary healthcare professionals in Indonesia
Source: PLoS One. 2025 Feb 10;20(2):e0315770. doi: 10.1371/journal.pone.0315770 (PMC11809861; doi:10.1371/journal.pone.0315770)
Supplement: Attachment — Submitted filename: Response to Reviewers.docx [file pone.0315770.s004.docx]

Dear Editors

Thank you for your helpful review and the opportunity to revise the manuscript

We have responded below to each of the comments and questions and trust this is satisfactory.

Kind regards,

Dr Fransisca H Agung

**Response to Reviewers**

| **Journal Requirements** | **Response** | **Revision** | |
| --- | --- | --- | --- |
| 1. Please ensure that your manuscript meets PLOS ONE's style requirements, including those for file naming. | Thankyou | Revisions have been made throughout.  Uploaded files have been renamed. | |
| 2. Please include a complete copy of PLOS’ questionnaire on inclusivity in global research in your revised manuscript. | We have completed this questionnaire and will upload it as a supplementary file. | | - |
| 3. Thank you for stating the following financial disclosure:  “This research was supported by Universitas Indonesia (Grant number NKB-NKB-1417/UN2.RST/HKP.05.00/2022)”  Please state what role the funders took in the study.  If the funders had no role, please state: "The funders had no role in study design, data collection and analysis, decision to publish, or preparation of the manuscript."  If this statement is not correct you must amend it as needed.  Please include this amended Role of Funder statement in your cover letter; we will change the online submission form on your behalf. | The researcher undertook this study as part of her doctoral studies, which were funded by a scholarship from Universitas Indonesia. However, beyond usual research supervisory processes, the funder had no role in study design, data collection and analysis, decision to publish, or preparation of the manuscript. | | We have added the following statement to the cover letter  “This research was supported by Universitas Indonesia (Grant number NKB-NKB-1417/UN2.RST/HKP.05.00/2022). The funder had no role in study design, data collection and analysis, decision to publish, or preparation of the manuscript.” |
| 4. We note that your Data Availability Statement is currently as follows: [All relevant data are within the manuscript]  Please confirm at this time whether or not your submission contains all raw data required to replicate the results of your study. Authors must share the “minimal data set” for their submission. | We believe that the summary of the results provides sufficient information for the study to be replicated. We have added a supplementary file of the qualitative interview and the quantitative data as part of the minimal data set. | | See Supplementary file 2. |
| 5. Please include your full ethics statement in the ‘Methods’ section of your manuscript file. In your statement, please include the full name of the IRB or ethics committee who approved or waived your study, as well as whether or not you obtained informed written or verbal consent. If consent was waived for your study, please include this information in your statement as well. | Thank you. This has been expanded as requested. | | For ethical approval and study permission, see **page 6 line 126 – 131**  For informed consent statement, see **page 7 line 157-159** and page **8 line 167-170** |
| **Questions** | **Reviewer 1** | | **Reviewer 2** |
| 1. Is the manuscript technically sound, and do the data support the conclusions? | No | | Yes |
| **Author’s response** | We consider that our conclusion is well supported by the data presented in the Results section. Our main conclusion states “This carefully designed online training program shows promise in increasing the quality of primary care professionals’ weight management counseling skills for adolescents.” This conclusion is based on the result that the training improved the knowledge and counselling skills of the health professionals.  We did not set out to test if the training intervention made a difference to patient outcomes, which will be the focus of future research. | | Thank you |
| 2. Has the statistical analysis been performed appropriately and rigorously? | No | | Yes |
| **Author’s response** | We believe that our statistical analyses were appropriate, as mentioned on **page 12 line 260– 267.** | | Thank you |
| 3. Have the authors made all data underlying the findings in their manuscript fully available? | Yes | | Yes |
| **Author’s response** | Thank you | | Thank you |
| 4. Is the manuscript presented in an intelligible fashion and written in standard English? | Yes | | No |
| **Author’s response** | Thank you | | The paper has been carefully reviewed and a number of minor changes made to enhance the quality of the English. |

Reviewer Comments

| Reviewer 1 | Answer | Revision and page |
| --- | --- | --- |
| When you are studying weight management in teenagers, your target group should be exactly teenagers and health professionals should have been trained during the intervention to help you. The text and title do not match. If you wanted to write this article, you should have only focused on the experts. If it is on teenagers, for example, you should have reported how much weight they have lost | Our research aim was to study the effectiveness of a training program that set out to improve health professionals’ counselling skills for adolescents. The design is an RCT but it did not set out to measure adolescent weight change, but rather, to establish whether or not the training program was able to enhance the knowledge and counselling skills of health professionals (the participants of the training) in the intervention group compared to the control group.  The evaluation of counselling skills was based on the quality of the recorded sessions that was undertaken by health professionals for ‘real world’ counselling of their overweight adolescent patients.    As such, we consider that our title is consistent with the manuscript. | No revision |
| Reviewer 2 |  |  |
| Advice for an overall edit of written English. | We would be pleased for the reviewer’s specific suggestions of where improvements are needed. The senior author is a native English speaker, a professor of adolescent health who has written over 380 papers (H-index 91, 47,000 citations), and who has been fully involved in the manuscript from its earliest phase. | The manuscript has been carefully reviewed and a number of changes have been made. |
| Line141-142: the authors mention that the participants completed a baseline survey. Do explain the purpose of finding the nutritional status and eating habits of the participants and how it impacts the study. | Thank you. We have provided more explanation in the text.  In essence, counselling on weight management requires behavioral change counselling skills. As part of the learning method, participants were required to develop their own behavioral change plan (line 162). This strategy was based on the Walsh and McPhee theory of behavioral change in which personal experience of working on behavior change is expected to help create empathy and self-efficacy that facilitates change with others. We have previously described this in our earlier paper describing the details of the development of the training program, which was also published by Plos One, and which we cite <https://journals.plos.org/>  plosone/article?id=  10.1371/journal.pone.0294986 | Additional text added  **Page 8 Line 161-165**  The training was developed using the Walsh and McPhee System Model for clinical preventive care that describes the importance of self-efficacy of health professionals in their counselling on behavioural change. This model recognizes that self-efficacy to counsel patients around healthy habits is influenced by health professionals’ own health status and behaviors. |
| Line 147: good to mention who had permission to access the website with resources. | Thank you, we have added this to the manuscript. | See revision on  **Page 8 Line 172 - 173**  The resources within the website were accessible once participants had created an account through the website and had registered for the training. |
| line 176: state which national guideline you are referring to and what is in it. give a reference to the guideline. | Thank you for this suggestion. | We have added these references (**page 9 line 201**) and in the reference list **(numbers 32-34).** |
| In the results section it is worthwhile to describe how the CG faired with the interviews. Currently you describe how the IG group did pre and post training. | Both groups (the control and intervention groups) were evaluated for their knowledge and skills using the same method. This is described in the Results section on line 268-372 (page 12-18)  Again, the qualitative assessment, which was undertaken by trained professional raters was of both groups. See lines 345-348 for an example of the qualitative assessment for the control group (CG). |  |
